# Theoretical Studies on the Binding Mode and Reaction Mechanism of TLP Hydrolase *kp*HIUH

**DOI:** 10.3390/molecules26133884

**Published:** 2021-06-25

**Authors:** Xixi Wang, Jiankai Shan, Wei Liu, Jing Li, Hongwei Tan, Xichen Li, Guangju Chen

**Affiliations:** Key Laboratory of Theoretical and Computational Photochemistry, Ministry of Education, College of Chemistry, Beijing Normal University, Beijing 100875, China; chemxixi@outlook.com (X.W.); jks@mail.bnu.edu.cn (J.S.); 201731150027@mail.bnu.edu.cn (W.L.); 201821150059@mail.bnu.edu.cn (J.L.); gjchen@bnu.edu.cn (G.C.)

**Keywords:** 5-HIU hydrolase, binding mode, hydrolysis mechanism, DFT calculation

## Abstract

In this work, we have investigated the binding conformations of the substrate in the active site of 5-HIU hydrolase *kp*HIUH and its catalytic hydrolysis mechanism. Docking calculations revealed that the substrate adopts a conformation in the active site with its molecular plane laying parallel to the binding interface of the protein dimer of *kp*HIUH, in which His7 and His92 are located adjacent to the hydrolysis site C6 and have hydrogen bond interactions with the lytic water. Based on this binding conformation, density functional theory calculations indicated that the optimal catalytic mechanism consists of two stages: (1) the lytic water molecule is deprotonated by His92 and carries out nucleophilic attack on C6=O of 5-HIU, resulting in an oxyanion intermediate; (2) by accepting a proton transferred from His92, C6–N5 bond is cleaved to completes the catalytic cycle. The roles of His7, His92, Ser108 and Arg49 in the catalytic reaction were revealed and discussed in detail.

## 1. Introduction

**Scheme 1:**
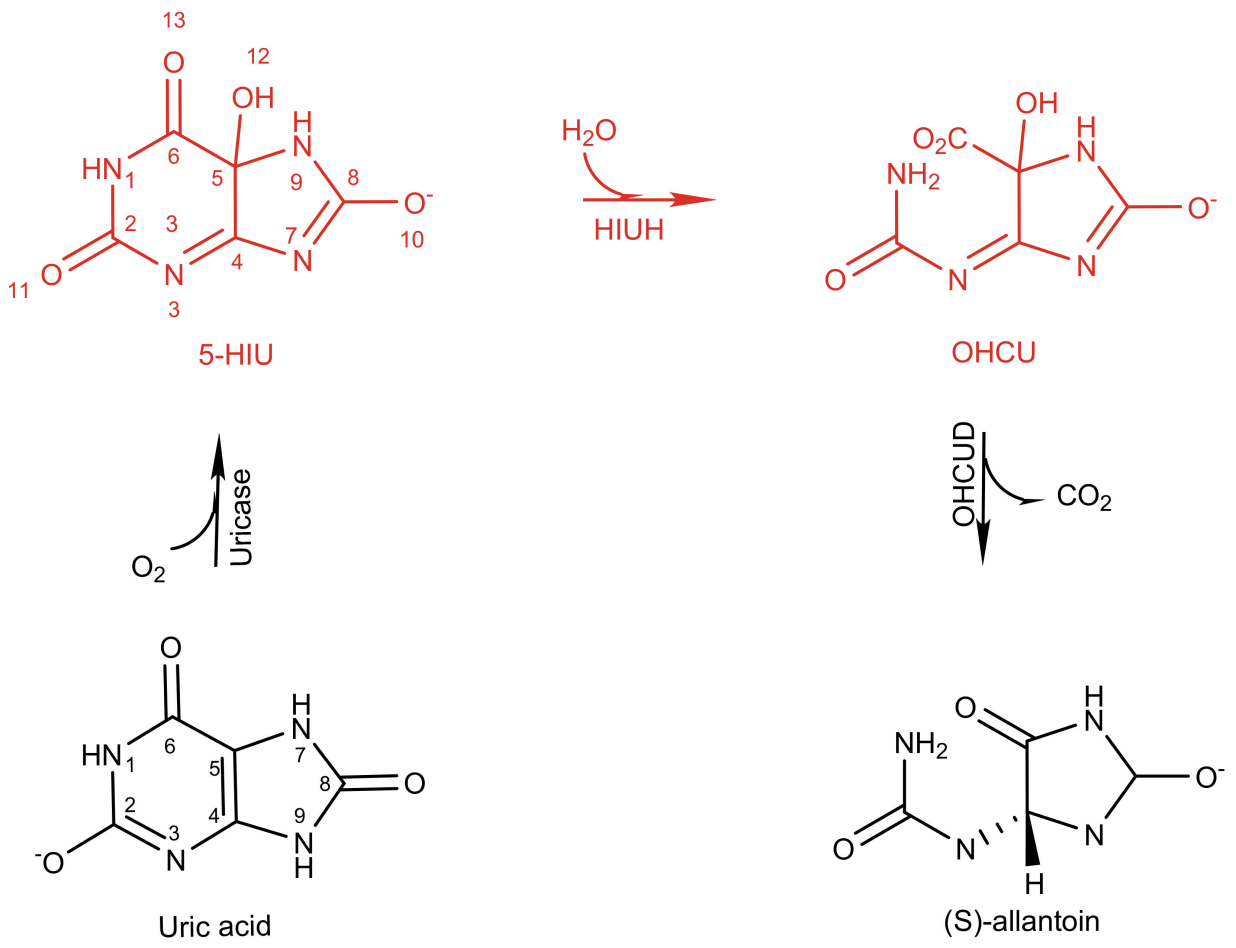
*Kp*HIUH catalyses hydrolysis of 5-hydroxyisourate (5-HIU) to generate 2-oxo-4-hydroxy-4-carboxy-5-ureidoimidazoline (OHCU) in the process of enzymatic conversion of uric acid to allantoin in *K. pneumoniae*.

Uric acid degradation is an important step of purine catabolism. Many plants and bacteria use it as a potential process to obtain nitrogen [1]. By converting uric acid into soluble allantonin, mammals reduce and prevent the formation of gout and urate kidney stones. Uric acid degradation follows a canonical three-step pathway (Scheme 1) in prokaryotes and eukaryotes [2]. Although it was believed that conversion from uric acid to allantonin is accomplished by a single enzyme urate oxidase [3], recent experiments revealed that two additional enzymes: 5-hydroxyisourate hydrolase (HIUH) and 2-oxo-4-hydroxy-4-carboxy-5-ureidoimidazoline decarboxylase (OHCUD), are also involved in the degradation process of uric acid [4,5]. In this scenario, HIUH hydrolyses 5-hydroxyisourate (5-HIU) to generate 2-oxo-4-hydroxy-4-carboxy-5-ureidoimidazoline (OHCU) (Scheme 1). OHCUD subsequently catalyzes the final step of uric acid degradation, leading to decarboxylation of OHCU on the C5 site to generate allantonin [6].

**Figure 1:**
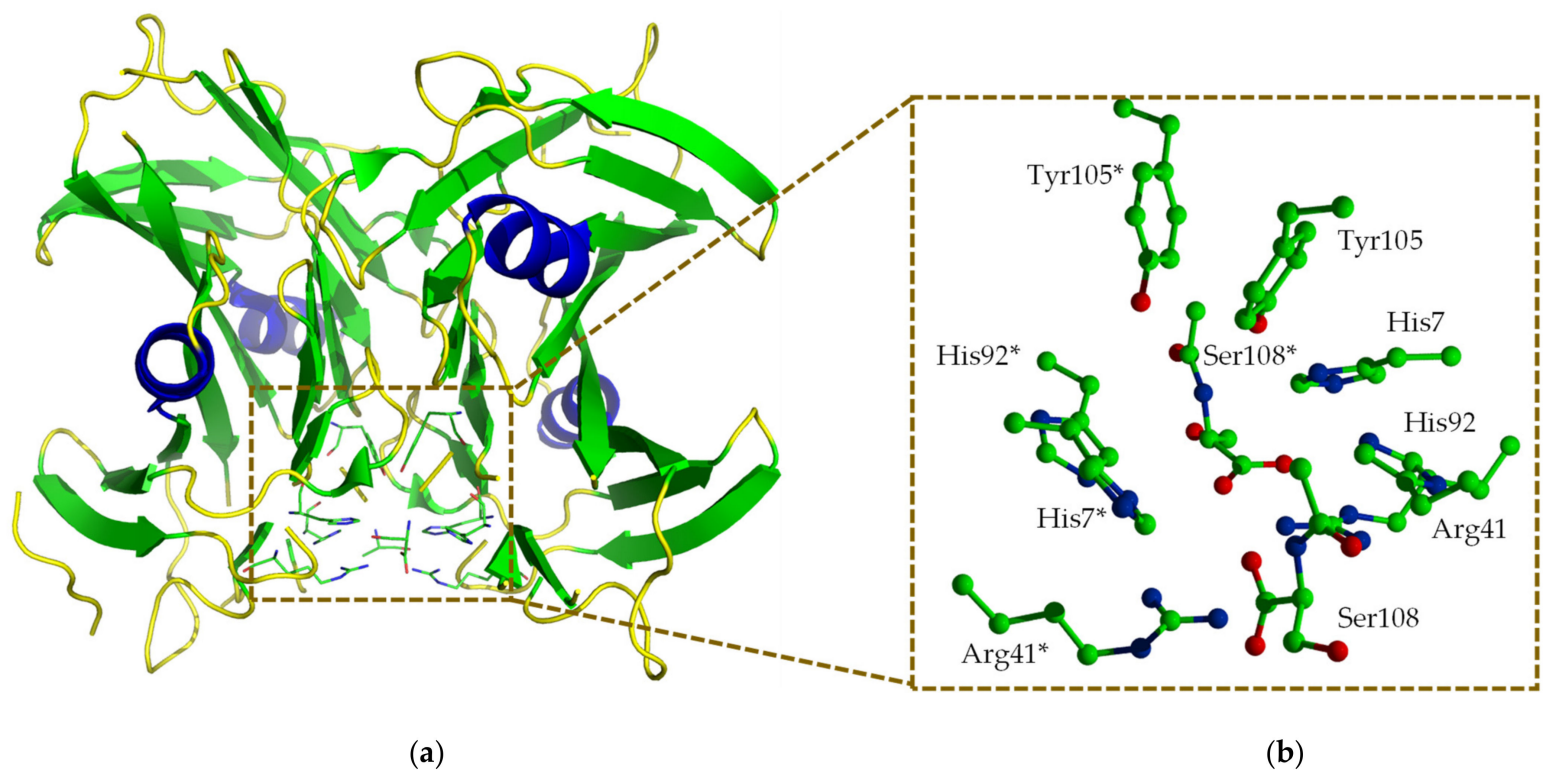
An overview of the X-ray structure of *Klebsiella pneumonia* 5-hydroxyisourate hydrolase (*kp*HIUH). One of the active center is located at the interface between two protein chains (**a**). The residues in the active center are arranged in a two-fold symmetry (**b**).

Interestingly, HIUHs from bacteria, fish and mammals were found to be highly similar to transthyretin (TTR) in the amino acid sequence. They were, therefore, designated as transthyretin-like proteins (TLPs) and have long been suspected to have a transport function. Later studies revealed that HIUH family is an enzyme progenitor of TTR, which losses enzyme function [7] but evolves to be a transport protein through mutation of critical residues for catalysis. The HIUH identified in *Klebsiella pneumonia* (*kp*HIUH) is a member of the TLPs. The X-ray structure of *kp*HIUH has been resolved recently, in which the *kp*HIUH protomer presents a β-sandwich structure which consists of nine β-strands and one α-helix (Figure 1a). In the crystal structure, four *kp*HIUH monomers of the enzyme form a homo-tetramer with 222 symmetry [1]. *kp*HIUH protomers assemble to dimer through interaction between the α-helices pairwise. Two dimers then form the tetramer with the extended β-sheets as the dimer–dimer interface. Two identical active sites of *kp*HIUH are formed at this β-sheet interface in the tetramer and situated near the surface of the proteins. In the active site of *kp*HIUH, the residues are arranged with a two-fold symmetry as shown in Figure S1 in the Supplement Material, which include the C-terminal tetrapeptide (Tyr105, Arg106, Gly107 and Ser108), two histidine (His7 and His92), a tyrosine (Tyr105) and an arginine (Arg41) from each of the two protein chains (Figure 1b).

**Figure 2:**
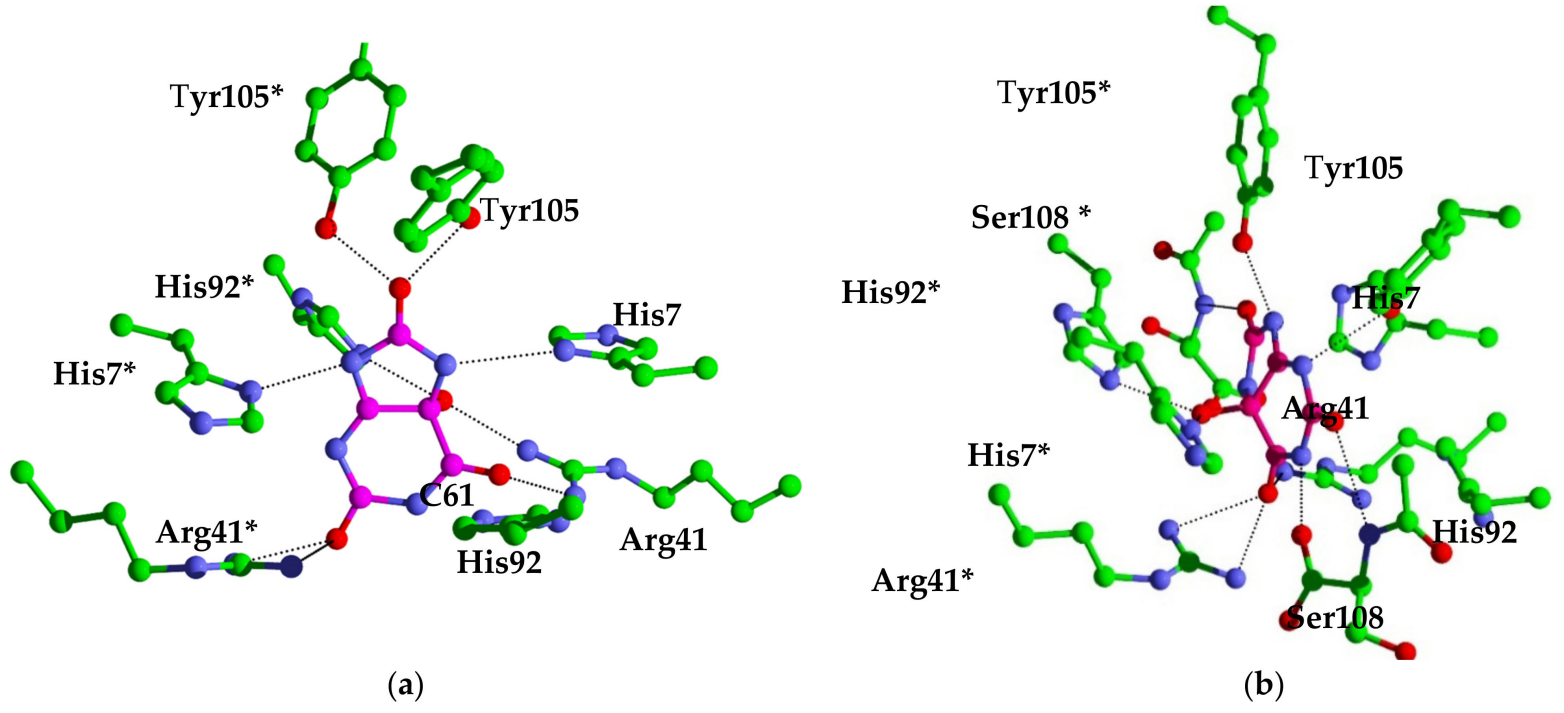
*Kp*HIUH active site. Residue names followed by an asterisk (*) are from the adjoining chain. carbon atoms from the protein are colored green, oxygen atoms are colored red, nitrogen atoms are colored blue, carbon atoms of 5-HIU are colored orange. (**a**) **complex 1** is the conformation proposed by French et al. (**b**) **complex 2** is docking result.

**Scheme 2:**
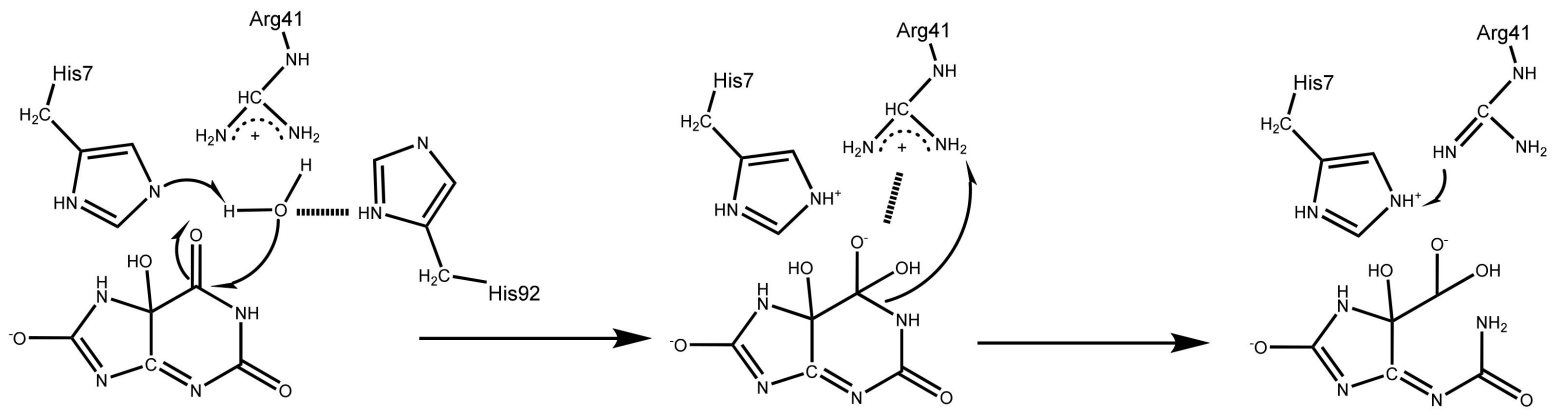
A mechanistic proposal by French et.al for the conversion of 5-hydroxyisourate to 2-oxo-4-hydroxy-4-carboxy-5-ureidoimidazoline by *kp*HIUH.

The sequence arrangement of the residues in the active center of *kp*HIUH was found to be very similar as those of serine hydrolases [8]. However, close examination of the conserved residues indicates that their relative positions and orientations are quite different in the two enzymes, suggesting that *kp*HIUH may possess different catalytic mechanism from serine hydrolases. *kp*HIUH catalyzes a very similar reaction to the amidohyrolase superfamily, which employs a mononuclear or binuclear metallic center to active the substrate and enhance hydrolytic water for nucleophilic attack [9]. However, lacking metallic cofactors in its active center, *kp*HIUH unlikely follows similar mechanism to amidohydrolases. Interestingly, an isozyme of *kp*HIUH, soybean HIUH, uses a covalent catalysis mechanism to hydrolyze 5-HIU, in which two glutamate residues act as the nucleophile and general acid catalyst, respectively [10]. *kp*HIUH differs remarkably from soybean HIUH in the gene primary sequences, especially exemplified by the lack of glutamate residues in the active site. There are four histidine residues (His7, His92 from two identical chains of the protein dimer) and two arginine residues (Arg41, Arg41*) symmetrically arranged around the substrate binding site in *kp*HIUH, which infers that it may adopt an acid/base catalytic mechanism. Site-directed mutagenesis experiments also confirmed the critical catalytic roles of these residues [1]. Both H7R and H92R mutations led to the inactivation of the enzyme. R41K mutation resulted in fivefold decrease in catalytic rate. Based on the X-ray structure of *kp*HIUH complexed with phosphate as a substrate analogue, French et al. proposed a binding mode of 5-HIU in the active pocket (Figure 2a) of *kp*HIUH and its possible catalytic hydrolysis mechanism. As shown in Scheme 2, in the proposed mechanism, the reaction is likely to be initiated by deprotonation of the lytic water molecule by His7. The deprotonated hydroxide then attacks the C6 site of the purine ring, leading to a tetrahedral oxyanion intermediate. After a proton being transferred from Arg41 to N1, oxyanion collapses and leads to ring opening. French et al. also indicated that the positively charged guanidyl side chain of Arg41 could stabilize the tetrahedral oxyanion intermediate in the reaction. Although this proposed mechanism is plausible, several aspects warrant further scrutiny. Kinetic experiments of the *kp*HIUH H92N mutant indicated that His92 is indispensable for the catalysis which, however, is not part of the proposed mechanism. Another noteworthy observation from experiments is that the R41K mutant of *kp*HIUH displayed fivefold lower activity than the wild-type enzyme despite that lysine is a better proton donor than arginine, which indicated that the role of Arg41 in the proposed mechanism is elusive. Moreover, the proposed mechanism is based on the protein-substrate binding mode determined from superposition of the substrate 5-HIU with 8-azaxanthine from the homologous PucM (PDB code 2H0E) structure [11]. In this case, except that the hydroxide group at the C5 site plays as a hydrogen bond donor to interact with His92 and His7 from one of the protein chains, O2, O6, O8 and N3 of 5-HIU also have several hydrogen bonding interactions simultaneously with the two protein chains from *kp*HIUH. Such a binding mode of 5-HIU which interacts with *kp*HIUH through multiple flexible hydrogen bonding interactions suggests that it might be able to adopt other feasible orientations in the active center and may, therefore, lead to alternative reaction mechanisms. In addition, as shown in Scheme 1, in the molecule of 5-HIU, there is more than one lactam group (C2-N1, C6-N1, C8-N7and C8-N9), thus how *kp*HIUH regiospecifically cleaves the C6-N1 bond remains elusive.

For the transient enzyme catalytic process, computational chemistry can provide insights not only into the binding-mode of the substrate with the enzyme but also the detailed reaction mechanism as a complementary tool to the experimental investigations. In the current work, by employing molecular docking and molecule dynamics (MD) simulations, an optimal binding conformation of 5-HIU in *kp*HIUH was proposed. Based on this conformation, density functional theory calculations were performed to study the catalytic mechanism of *kp*HIUH. The mechanism presented in this study agrees well with experimental observations. Our calculations revealed that the 5-HIU hydrolysis reaction consists of two stages: (1) the lytic water molecule is deprotonated by His92 and carries out nucleophilic attack on C6 carbonyl of 5-HIU, resulting in an oxyanion intermediate. (2) His92 transfers a proton to the N1, which results in C6–N1 lactam bond cleavage to complete the catalytic cycle. In this mechanism, His92 and His7 play critical roles to promote the hydrolysis reaction. One pair of His92 and His7 is involved in the hydrogen bonding with the C5-OH group to expose the C6 site to the other pair of His92 and His7 so that they are able to deprotonate the lytic water to initiate the hydrolysis reaction. Two Arg41 residues from two protein chains not only sever as hydrogen bonding donors to anchor the C6=O carbonyl of the substrate, but also polarize the carbonyl to facilitate the reaction. Two C-terminal Ser108 residues of the enzyme use the backbone amino and carboxylate groups to form hydrogen bonding interactions from two opposite directions of the substrate, which orientates the substrate to adopt a favorable conformation for hydrolysis. 

## 2. Results and Discussions

### 2.1. Molecular Docking Study of the Binding Mode of 5-Hydroxyisourate (5-HIU) in Klebsiella pneumonia 5-Hydroxyisourate Hydrolase (kp*HIUH*)

French et al. proposed a possible binding mode of 5-HIU to *kp*HIUH by superposition with 8-azaxanthine from the homologous PucM structure [11]. A similar *kp*HIUH/5-HIU complex was also modeled in this study. As displayed in the Figure 2a, the substrate 5-HIU was proposed to interact with Arg41 and Arg41* using O11 and O13 as two hydrogen bond acceptors. O8 of the five-membered ring establishes two hydrogen bonds with a pair of Tyr105 from two protein chains simultaneously. The C5 hydroxyl group of 5-HIU has two hydrogen bonding contacts with His92* and Arg41. These hydrogen bonds between 5-HIU and the protein align the substrate in the active site so that the 1–8 axis of the molecule (from N1 to C8) almost overlaps the 2-fold axis of two *kp*HIUH chains with the molecular plane being perpendicular to the dimer interface. Such a conformation of 5-HIU exposes its C6 site to His7 and His92 which were, therefore, proposed to activate the lytic water to attack on the C6 in the catalytic reaction. However, the spatial geometric structure of the complex indicated that His7 and His92 are located on the two sides of the molecular plane of 5-HIU separately, so that only His92 is able to assist the lytic water to attack on the C6 site.

Upon closely examining 5-HIU and the active site of *kp*HIUH, one can find that they both have multiple hydrogen bond acceptor and donor sites. Therefore, it is reasonable to speculate that 5-HIU might be able to adopt alternative binding modes in the active pocket. Thus, we carried out molecular docking analyses to search for possible binding conformations of 5-HIU based on the crystal structure of *kp*HIUH (PDB code 3QVA) [1]. Interestingly, docking calculations indeed yielded a distinct binding conformation of 5-HIU. As shown in Figure 2b, the docked complex showed that the substrate 5-HIU is sandwiched between two protein chains. Two Tyr105 residues interact with N3 and N9 of 5-HIU from two sides of the molecular plane through hydrogen bonds, while O6 binds with two Arg41 residues simultaneously. The C5–OH group of 5-HIU interacts with His7* and His92* through hydrogen bonding interactions. Ser108 from chain A has two hydrogen bonding interactions with C2–O and N1–H through its backbone amino group and terminal carboxylate respectively, while Ser108* makes two hydrogen bonds with C8–O and N7–H in a similar mode. Compared to the binding conformation proposed by French et al., 5-HIU is rotated clockwise for ~90° around the 4–5 axis (from C4 to C5) and the 1–8 axis sequentially. In this complex obtained from molecular docking, the 4–5 axis instead of the 1–8 axis of 5-HIU overlaps with the two-fold axis of the protein. It is worth mentioning that the 4–5 axis and 1–8 axis can be seen as two pseudo 2-fold axes of the uric acid molecule, which presents a planar conformation without C5–OH group. Therefore, the two binding conformations of 5-HIU in the 2-fold symmetry active site of *kp*HIUH, including that proposed by French et al. and that obtained from the docking experiment, are actually two quasi symmetry-adapted complexes. In addition, since the active center of *kp*HIUH is constituted by two identical protein chains with two-fold symmetry, the substrate can adopt two symmetrically equal conformations by rotating 180° around the two-fold axis of the protein.

**Figure 3:**
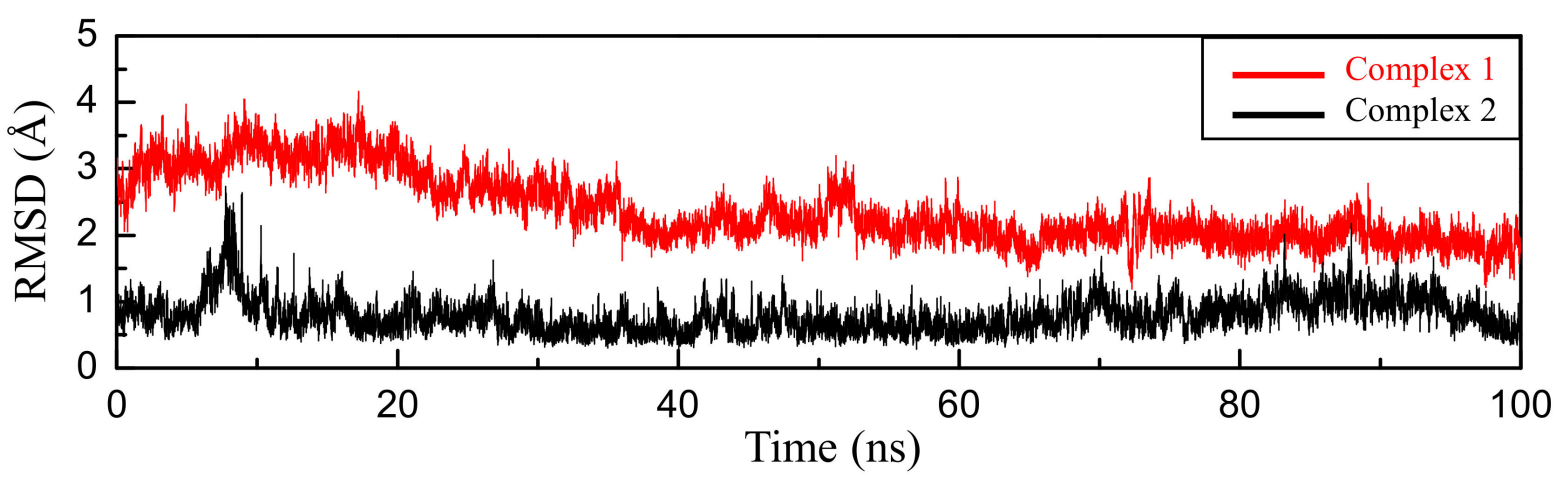
RMSD (Å) plot of substrate 5-HIU during 100 ns simulations, the final ligand conformation from **complex 2** was used as the reference, the RMSD of **complex 1** is marked with red and the **complex 2** is black.

**Figure 4:**
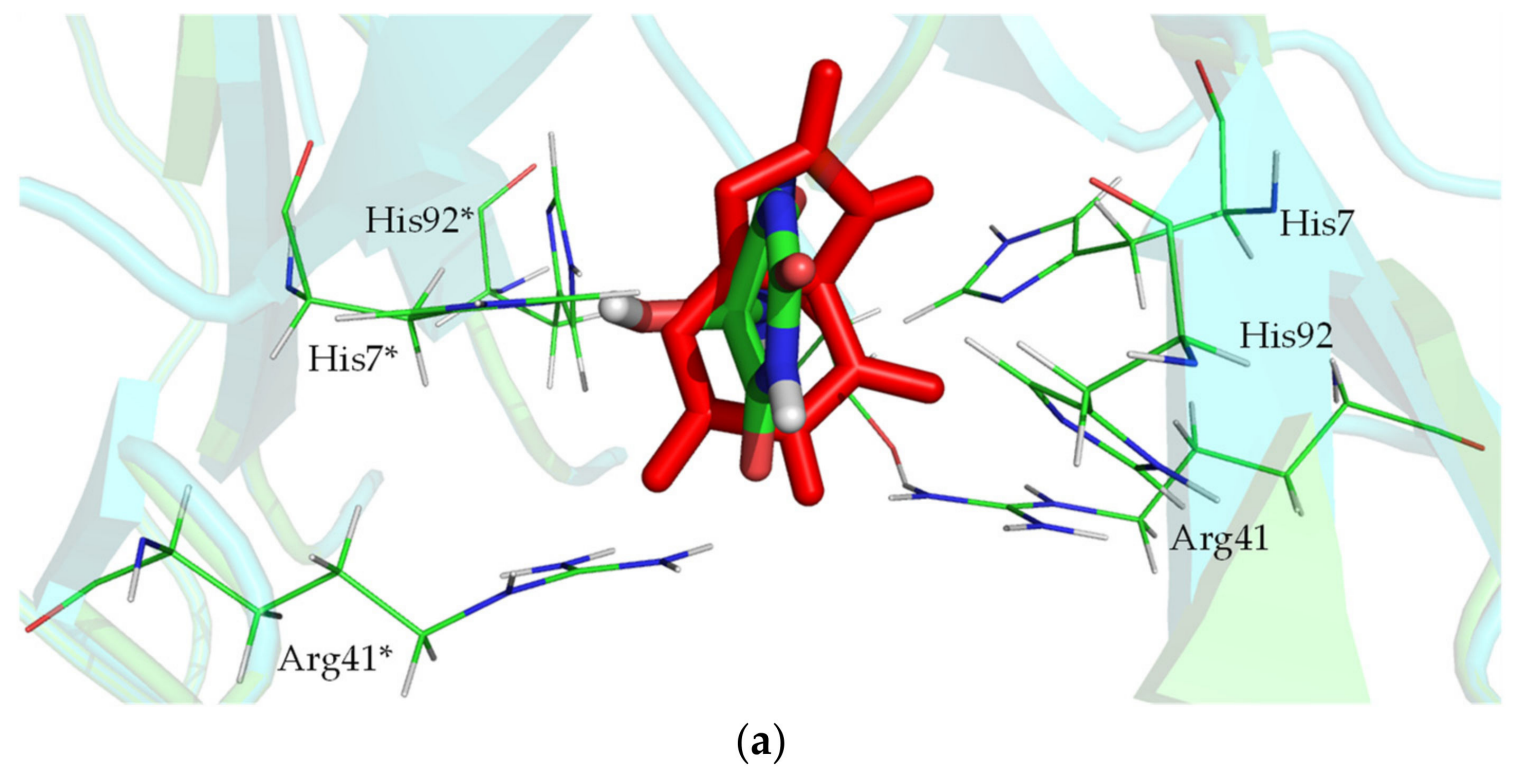

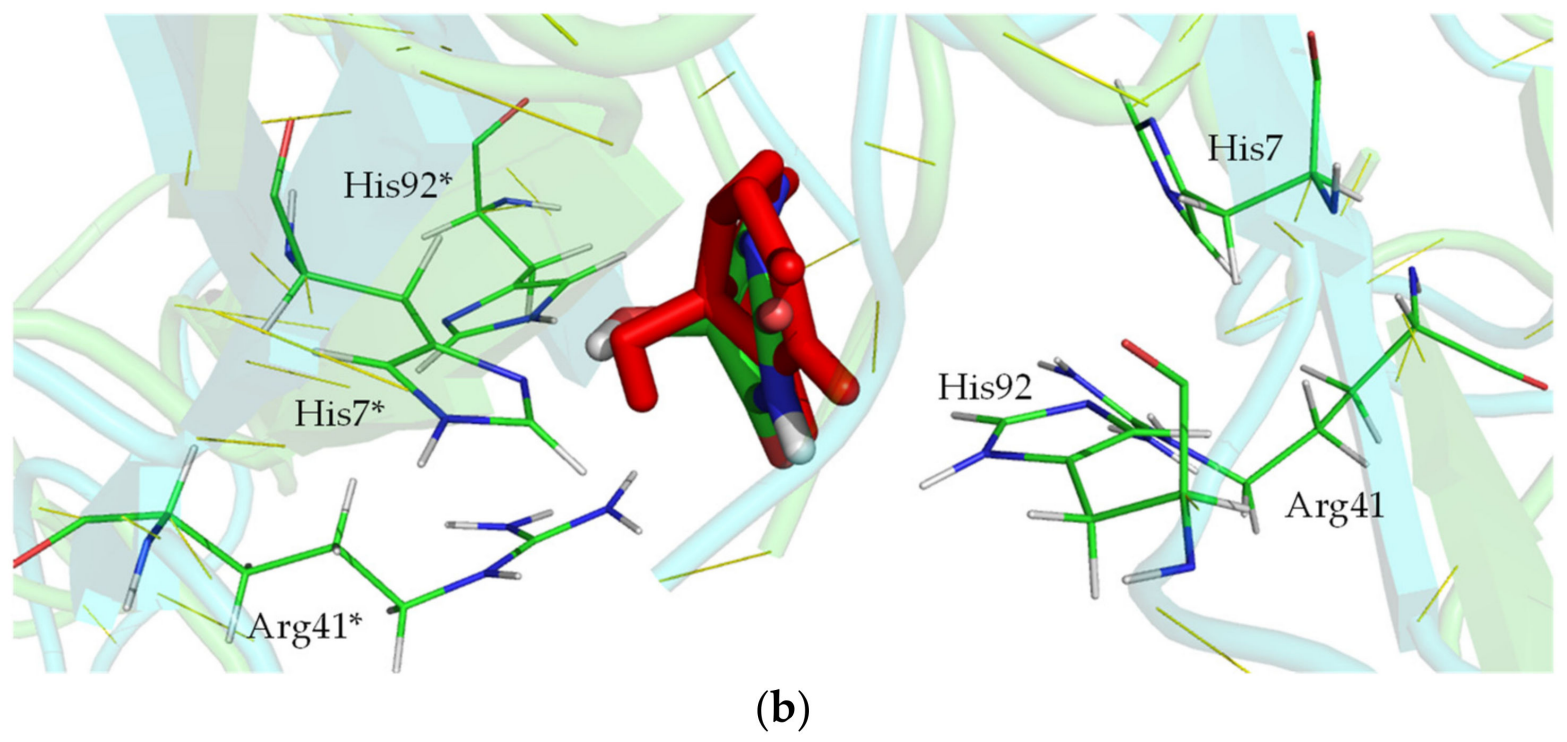
Overlaying 5-HIU of **complex 1 (colored with red)** with **complex 2 (colored by elements)** along their MD trajectories: (**a**) the initial structures; (**b**) the final snapshots.

To further examine if 5-HIU is able to steadily bind in the active site of kpHIUH, 100 ns molecule dynamics (MD) simulations were performed for two different kpHIUH/5-HIU complexes in which 5-HIU adopts the conformation proposed by French et al. (**complex 1**) or as obtained from docking calculation (**complex 2**) respectively. Notably, **complex 2** showed well converged root-mean-squared deviation (RMSD) during the 100 ns simulation, which indicates that the docked conformation is a stable binding one. Whereas **complex 1** displayed distinct dynamics during MD simulation. During the heating stage, a significant structural variation of the substrate 5-HIU was observed. Visual inspection of the trajectory revealed that the substrate experienced an orientation conversion from its initial conformation to the one as that in **complex 2**. As shown in Figure 3, the RMSD plot of two complexes during 100 ns also shows that **complex 1** gradually converge to **complex 2**. By overlaying **complex 1** with **complex 2** (Figure 4a), it is founded that at the initial stage, the substrate adopted different orientations in the two complexes. The RMSD of the substrate molecule between two complexes is 3.7 Å. After 100 ns simulation, the RMSD of the substrate between two complexes decreased to 1.0 Å (Figure 4b). The MD simulations therefore indicated that the docked conformation is the dominant one. Hence the following discussion is based on the structure of **complex 2**.

Based on the 100 ns MD simulation trajectory of **complex 2**, the CPPTRAJ program was used to analyze the hydrogen bonding occupancy between the substrate and the residues from *kp*HIUH protein. Hydrogen bonds are determined using geometric criteria with the donor to acceptor heavy atom distance of 3.5 Å and the donor-hydrogen-acceptor angle of 120°. As listed in Table 1, Tyr105 and Ser108 from both of the two protein chains and Arg41* from chain D had persistent hydrogen bonding interactions with the substrate during 100 ns simulation, while His92* and His7* formed two stable hydrogen bonds with the C5–OH group of the substrate and pulled it towards chain D so that the two rings (five membered ring and purine ring) of the substrate appeared to move towards chain A, which exposed C6-N1 bond to the water molecule residing between His7 and His92. Such a binding conformation of 5-HIU facilitates the hydrolysis reaction to regiospecifically proceed on the C6-N1 bond. 

An important structural feature of **complex 2** is that Ser108 and Ser108* are able to make hydrogen bonds with the substrate from two terminals of the 1–8 axis in this conformation. As mentioned above, these hydrogen bonding interactions formed by the backbone groups of two Ser108 orientate the substrate in the active site. Previous experiments indicated that S108A mutation only led to a very small catalytic efficiency reduction of *kp*HIUH [1,11], whereas deletion of the conserved C-terminal tetrapeptide including Ser108 greatly suppressed the catalysis [11]. These experimental observations could be well rationalized by the structure of **complex 2** in which Ser108 uses the backbone but not side chain to form hydrogen bond with the substrate to stabilizes it. However, the hydrogen bonding interactions between 5-HIU and Ser108 are not established in **complex 1**. Interestingly, Jung et al. have resolved the crystal structure of PucM in complex with the urate analog 8-azaxanthine (AZX) (PDB code 2H0F) [11], in which AZX molecule adopts a conformation as that 5-HIU in **complex 1**. Compared to 5-HIU, AZX lacks the C8=O carbonyl and its N7 site is deprotonated, which therefore inhibit it from forming hydrogen bonding interactions with Ser108 through the purine ring as 5-HIU. Moreover, HIU-5 is anchored by His7 and His92 through its C5–OH group in **complex 2**. AZX lacks the hydroxyl substituent on C5 site, which also leads to its rotation to adopt an alternative conformation to better interact with two histidines. 

### 2.2. Hydrolysis Mechanism of 5-HIU Catalyzed by kp*HIUH* Based on Complex ***2***

**Scheme 3:**
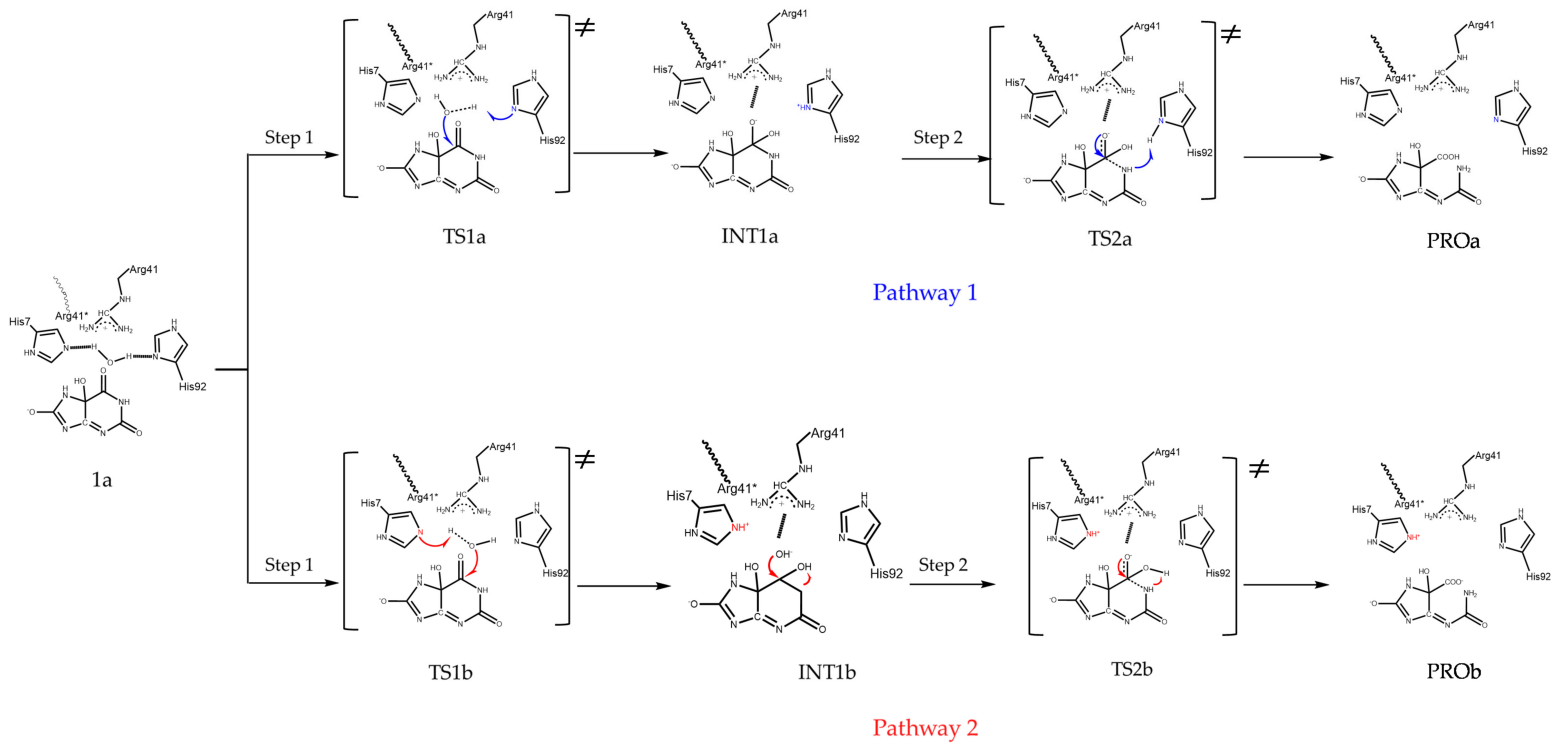
Two possible pathways for 5-HIU hydrolysis by *kp*HIUH.

Since both of the docking experiment and the MD simulation confirmed that **complex 2** is the preferential binding complex of 5-HIUand *kp*HIUH, the final snapshot was then extracted from its product phase to build the cluster model for further catalytic mechanism investigations. According to the geometric arrangement of the residues relative to 5-HIU in **complex 2**, we proposed two possible catalytic mechanisms. As shown in Scheme 3, in **complex 2**, the lytic water is located adjacent to C6 of the substrate and has hydrogen bonding interactions with His92 and His7 from chain A. Each of these two histidine residues could deprotonate the lytic water to trigger the catalytic reaction. After addition of hydroxide to C6, the generated tetrahedral C6 oxyanion intermediate is stabilized by the nearby positively charged Arg41 and Arg41*. In the second step of the reaction, to accomplish C6–N1 bond cleavage, a proton needs to be added on the N1 site. Depending on whether the lytic water is deprotonated by His92 or His7 in the first step, the proton transfer could proceed through an intermolecular mode (**Pathway 1**) or an intramolecular mode (**Pathway 2**). 

**Figure 5:**
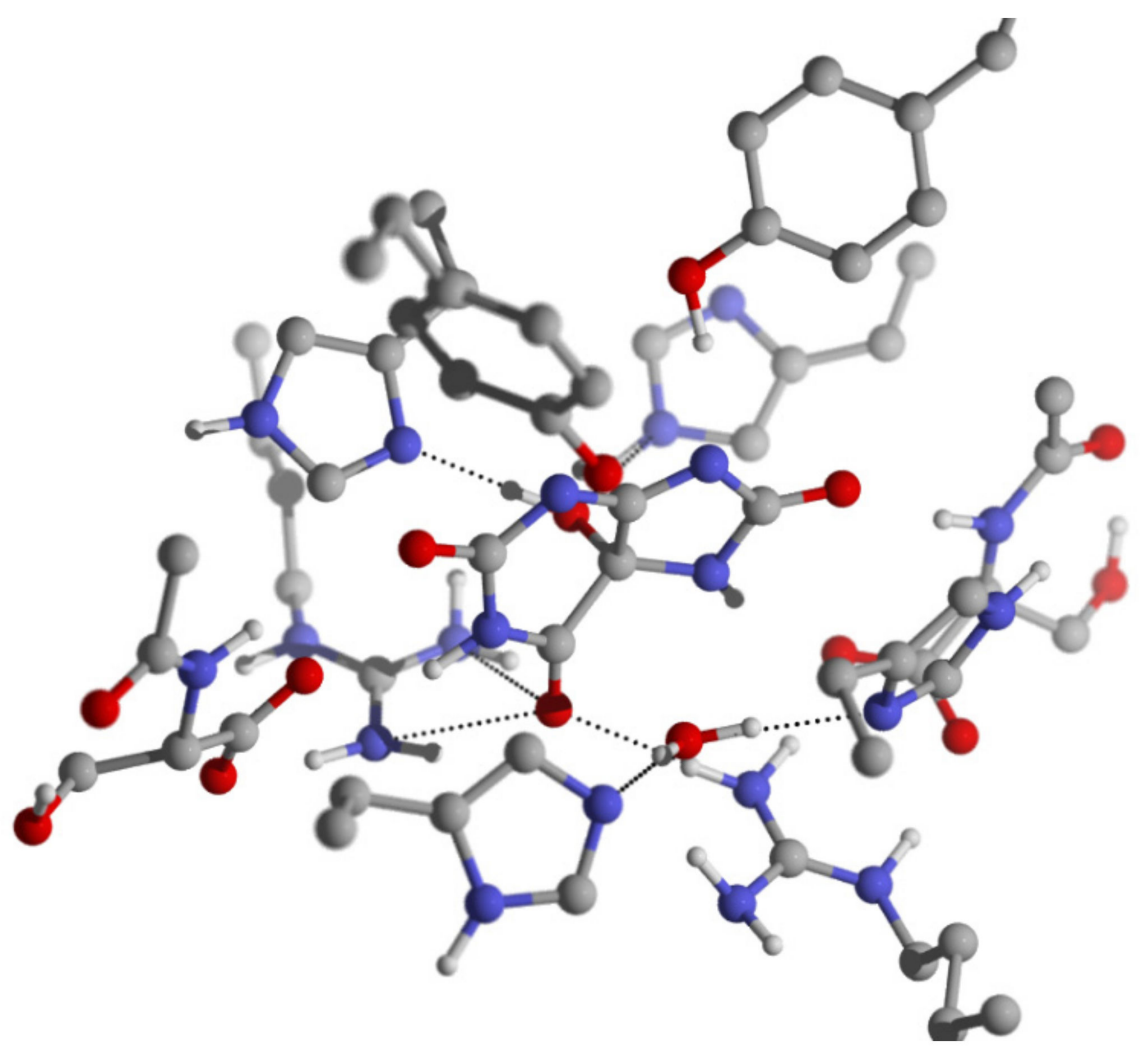
The geometry structure of **1a**, some important bond lengths are labeled in the unit of Å.

Based on the cluster model built from **complex 2**, the reactant **1a** was optimized. In the obtained **1a**, the carbonyl C6=O of the substrate resides between the two guanidinium groups of Arg41 and Arg41* and forms three hydrogen bonds with them (Figure 5). The O-N^ω^_Arg41_, O-N^ω^_Arg41*_ and O-N^ω1^_Arg41*_ distances are 3.19 Å, 2.91 Å and 2.96 Å, respectively. These hydrogen bonding interactions and the positive charge carried by the two guanidiniums polarize the C6=O bond, which shows a stretched C6=O bond length of 1.25 Å with corresponding Wiberg bond order of 1.51. The Mulliken charges of C6 and O are 0.69 and −0.60 respectively. The C5-OH group of 5-HIU, which is located on the opposite side of molecular plane from the lytic water, forms two hydrogen bonds with His7* and His92*. Besides these hydrogen bonds formed around the reaction site, there are several other hydrogen bonds formed between the substrate and the protein. The detailed structure parameters of these hydrogen bonds are summarized in Table 2. These hydrogen bonds firmly stabilize 5-HIU molecule in a conformation which exposes C6 to the lytic water. In **1a**, the oxygen atom of the lytic water departures from C6 for 2.81 Å, while His7 and His92 form two hydrogen bonds with the lytic water with distances of 2.92 Å (N^δ^_His7_-O) and 2.79 Å (N^ε^_His92_-O) respectively. Although both of the two histidine residues are able to deprotonate the lytic water to initiate the catalytic reaction, His92 shows higher potential than His7. 

**Figure 6:**
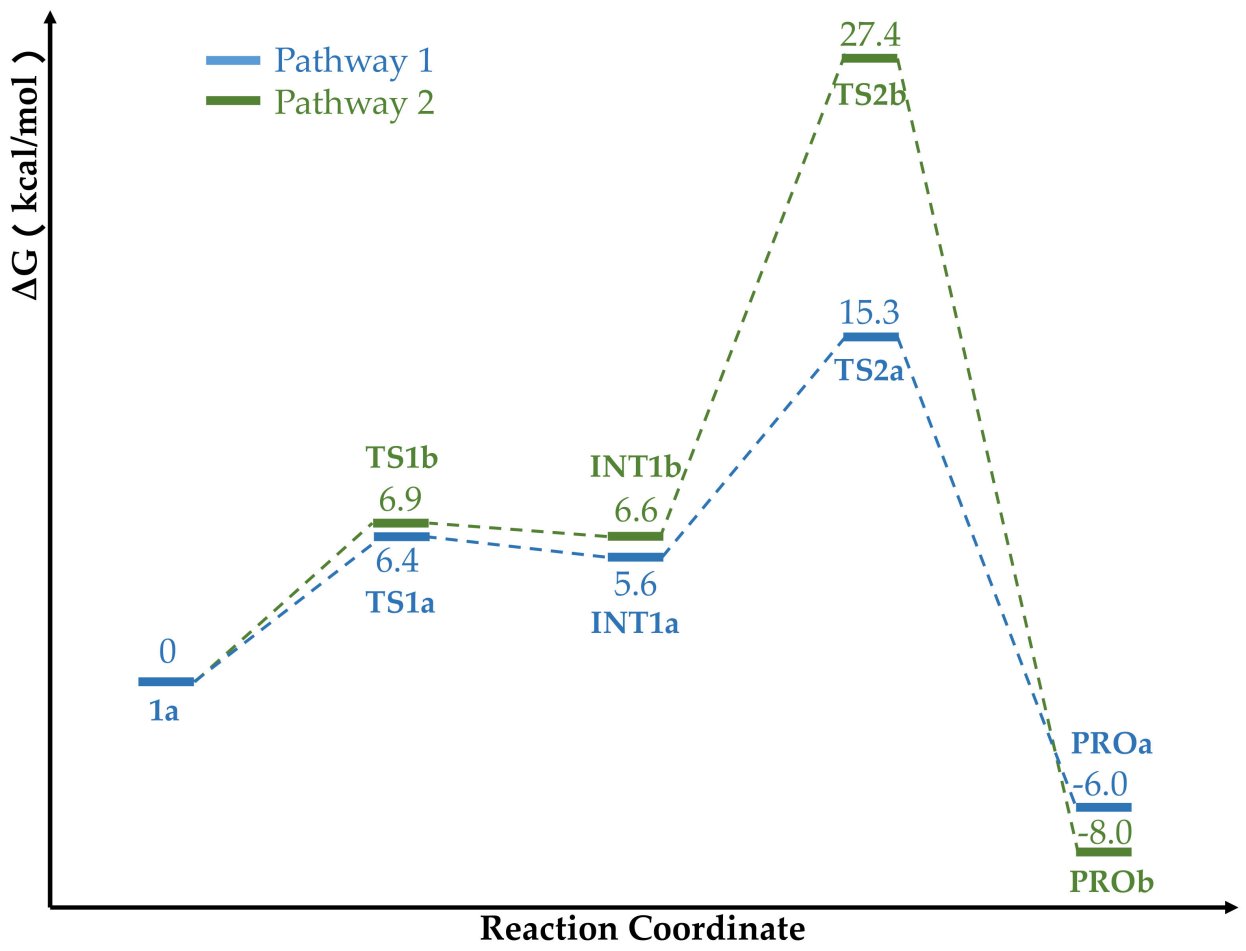
Free energy profile of the **pathway 1** and **pathway 2**. The relative energies are labeled in kcal/mol.

Starting from the reactant **1a**, we explored two possible hydrolysis pathways of 5-HIU. The calculated energy profiles of the two pathways are shown in Figure 6. Both of the two pathways are rate-limited by the second step in which C6-N1 bond cleaves, while **pathway 1** is kinetically 12.1 kcal/mol favorable than **pathway 2** in this rate-limiting step. It is worth mentioning that the activation barrier of the rate-limiting step deduced from the experiment is 14.4 kcal mol^−1^ [1], which agrees well with 15.3 kcal mol^-1^ that is obtained for **pathway 1** from our calculations.

**Figure 7:**
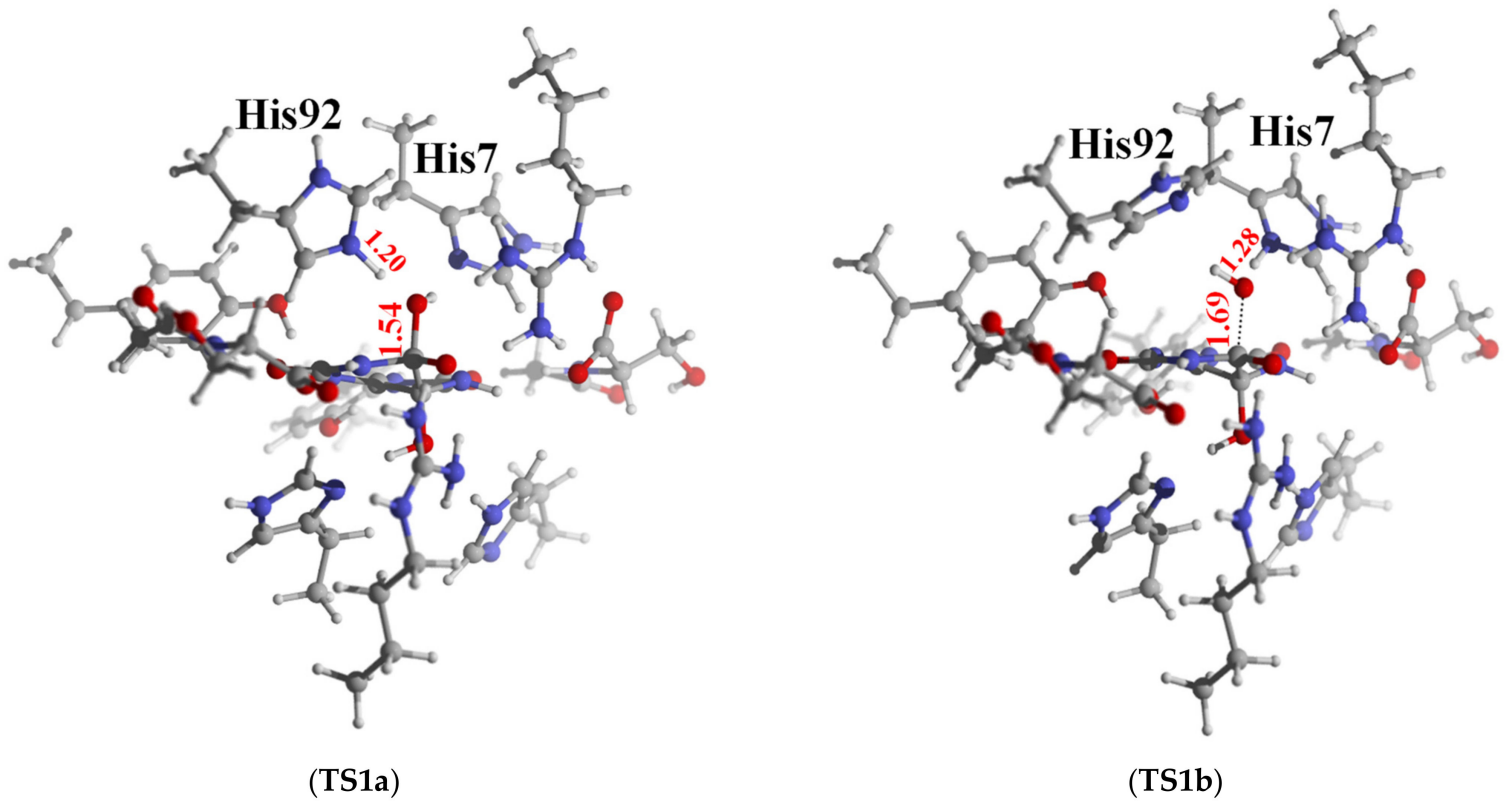
The geometry structure of **TS1a** and **TS1b**; some important bond lengths are labeled in the unit of Å.

**Pathway 1** and **2** experience very similar energy barriers in the first reaction step, which are 6.4 kcal/mol for **pathway 1** and 6.9 kcal/mol for **pathway 2** respectively. In Table 3, we summarize some important structural parameters of the two transition states (**TS1a** and **TS1b**). Due to the fact that His92 locates in a shorter distance from C6 than His7, the two transition states present distinct structural characteristics. Specifically, **TS1a** is a late transition state while **TS1b** is an early one as shown in Figure 7. As evidenced by the structural parameters listed in Table 3, in **TS1a**, the deprotonation of the lytic water by N^ε^ of His92 is almost accomplished, while in **TS1b**, the proton transferring still stays at the initial stage, which accordingly results in hydroxide addition on C6 to proceed in different phases in the two transition states. The distances of the oxygen atom of the lytic water to C6 in **TS1a** and **TS1b** are 1.54 Å and 1.42 Å, respectively. It should be noted that Arg41 and Arg41* play important roles in promoting this first reaction step in both pathways. Their positive charge strongly polarizes the C6=O bond, which promotes its conversion to a single C6–O^-^ bond in this first step by which C6 is converted from *sp^2^* to *sp^3^* hybridization. In **TS1a**, C6=O forms four hydrogen bonds with the two guanidine groups from Arg41 and Arg41* simultaneously. The distances of O–N^ω^_Arg41_, O–N^ω1^_Arg41_, O–N^ω^_Arg41*_ and O–N^ω1^_Arg41*_ are 2.83 Å, 3.28 Å, 2.89 Å and 2.94 Å respectively. In **TS1b**, C6=O group also has four hydrogen bond interactions with Arg41 and Arg41*. Along with C6 and the lytic water approaching each other, the C6=O carbonyl moves slightly towards the Chain A side, so that the two hydrogen bonds formed between C6=O and Arg41 (O–N^ω^_Arg41_ = 2.80 Å and O–N^ω1^_Arg41_ = 3.09 Å) are shorter than the two formed between it and Arg41* (O–N^ω^_Arg41*_ = 3.42 Å and O–N^ω1^_Arg41*_ = 2.88 Å). These hydrogen bonds stabilize the transition state. Arg41 has been identified to play a role in the catalysis process. French et. al conducted a R41K mutation experiment of *kp*HIUH and found a five-fold decrease of its catalytic rate [1]. Another experiment of PucM, a homologue of *kp*HIUH, also displayed decreased 5-HIU decomposition rate upon R49K mutation (Arg47 is the corresponding residue in PucM as Arg41 of *kp*HIUH) [11]. A rational explanation to these two experiments is that Arg to Lys mutation weakens the hydrogen bond interactions between the enzyme and the C6=O group. Specifically, compared to the guanidyl side chain of arginine which has two hydrogen bond donor sites, the ammonium group of lysine can only form one hydrogen bond with C6=O. In addition, the experiment also found that R49E mutation essentially abolished the activity of PucM [11], which should be attributed to the fact that the negative charged glutamate cannot form hydrogen bond with C6=O to stabilize HIU-5 in the active site for hydrolysis. 

Although the two transition states are quite different in their geometry structures, the intermediates in two reaction pathways (**Int1a** and **Int1b**) are very similar. The two intermediates are also very close in their energy with **Int1a** slightly lower by 1.0 kcal/mol. In both **Int1a** and **Int1b**, C6 presents a tetrahedral conformation with C6–N1 bond stretching to 1.46 Å. The C6 carbonyl bond converts to a C–O^−^ single bond, with bond length of 1.33 Å. The calculated Mulliken charge population on the O^−^ is −0.72 in **Int1a** and −0.74 in **Int1b**. C6=O group keeps the four hydrogen bond interactions with two Arg41 from two protein chains in these two intermediates. The corresponding hydrogen bond lengths are listed in Table 4.

In both **Int1a** and **Int1b**, the terminal carboxylate group of Ser108 acts as a hydrogen bond acceptor to form a hydrogen bond with N1-H of the substrate. The O_Ser108_–N1 distances in two intermediates are 2.91 Å and 2.92 Å respectively. This hydrogen bonding interaction is able to increase electron population on N1 and facilitate it to accept proton in the second reaction step. Therefore, in addition to assisting the substrate 5-HIU to orientate in the active site, Ser108 also plays a role in promoting the catalytic reaction. Moreover, as a C-terminal residue, Ser108/Ser108* also plays an important role to stabilize the positive charged arginine in the active center. In all optimized reaction species, there are steady salt-bridge interactions between the carboxylate group of Ser108 and the guanidyl group of Arg41 (also between Ser108* and Arg41*), which maintains the geometric structure of the active center. In **Int1a** and **Int1b**, the hydroxide group added on the C6 site has two hydrogen bond interactions with His7 and His92 simultaneously. The hydroxide group serves as both hydrogen bond donor and acceptor in this hydrogen bond network, which results in synergistic enhancement of two hydrogen bonds. In **Int1a**, the protonated His92 locates in a very close distance to the C6 site of the substrate, so that it forms a very strong hydrogen bonding with the hydroxide (O–N^ε^_His92_ = 2.49 Å), which in turn strengthen the hydrogen bonding between the hydroxide and His7(O–N^δ^_His7_ = 2.79 Å). In contrast, in **Int1b**, the relatively weak hydrogen bonding between hydroxide and protonated His7 (O–N^δ^_His7_ = 2.70 Å) also limits its influence on the hydrogen bond between hydroxide and His92(O–N^ε^_His92_ = 2.95 Å). Therefore, although the two intermediates are very similar in their structures, **Int1a** is slightly lower in energy (only 1.0 kcal/mol) than **Int1b**. 

Since the C6–OH group points towards two different histidine residues in two directions in the **Int1a** and **Int1b**, the second reaction step bifurcates into intramolecular proton transfer and intermolecular transfer modes (As shown in Scheme 3). In **Int1a**, His92 has accepted a proton from the lytic water in the first step and locates adjacent to the N1 site of the substrate. The calculated Mulliken population of N1 is −0.61 in **Int1a**, which hence could easily accept a proton from His92 and induce C6–N1 bond cleavage. From **Int1a**, it costs 9.7 kcal/mol energy to reach the second transition state (**TS2a**). This reaction step is also rate-limiting in **pathway 1** with a global energy barrier of 15.3 kcal/mol. In the optimized **TS2a**, His92 breaks down the hydrogen bond with the C5–OH group. Its N^ε^H moiety turns to point towards the N1 site of the substrate. The proton locates in the middle of two nitrogen atoms with N^ε^_His92_–H and N1–H distances of 1.23 Å and 1.30 Å respectively. The proton transferring from His92 to N1 activates C6–N1 bond, which stretches to 1.55 Å in the **TS2a**. Such a structural change from **Int1a** to **TS2a** indicates that the second reaction step is a concerted one with associative characteristics, supported by the 0.81 Wiberg bond index(order) of C6–N1 and 0.32 of N1–H_His92_, respectively. In the **TS2a**, C6–O^-^ bond length is 1.33 Å, whereas the C6–OH bond is shortened to 1.42 Å, which should be ascribed to the loss of hydrogen bond between C6–OH and His92. His7, on the other hand, remains to be hydrogen bonded with C6–OH with a length of 2.78 Å. In **TS2a**, with elongation of the C6–N1 bond, C6 converts from tetrahedral conformation to a planar one, which rotates C6–O^−^ moiety towards chain D so that two hydrogen bonds between C6–O^-^ and Arg41 are weakened, while the two hydrogen bonds formed with Arg41* are strengthened (Table 4). By crossing over the transition state **TS2a**, the reaction system reaches the product of **pathway 1** (**PROa**) and completes the catalytic cycle. The whole reaction of **pathway 1** is exothermic with −6.0 kcal/mol. Among all residues in the active center, His92 plays the most critical role in this reaction pathway which deprotonates the lytic water and delivers the proton to N1 in two reaction steps respectively. It is in accordance with the results from the mutation experiments that H92R mutation led to the full inactivation of the enzyme [1].

From **Int1b**, the reaction could follow an alternative pathway (**pathway 2**). the second step of **pathway 2** needs to cross over an energy barrier as high as 27.4 kcal/mol to reach the transition state **TS2b**. Figure S5 depicts the optimized **TS2b**.In this transition state structure, the hydroxide of the C6 site breaks off its hydrogen bond with His92 and turns towards N1. The H–O–C6–N1 dihedral angle decreases to 0.69° from 56.7° in **Int1b**. The C6–OH bond bends towards the N1 site, so that the H–O–C6 angle distorts to 93° and the O–N1–C6 angle bends to 81°, which reflects the high tension in the geometric structure of this transition state. The H–O bond length of the hydroxide slightly stretches to 1.05 Å. In the meantime, the C6–N1 bond is significantly elongated to 2.19 Å. The calculated Wiberg bond order of C6–N1 bond is only 0.25, which indicates that this second step of **pathway 2** follows a dissociative mechanism. The Mulliken charge of C6 and N1 are 0.67 and −0.76 respectively, corresponding to heterolytic cleavage of the C6–N1 bond. Along with the breakage of the C6–N1 bond, C6 converts from *sp*^3^ hybridization to a planar *sp*^2^ conformation. The C6–O^-^ bond shortens to 1.25 Å correspondingly. Its Wiberg bond order increases to 1.33. The Mulliken charge of the O^−^ increases to −0.51. The shortening of C6–O^-^ weakens its four hydrogen bond interactions with Arg41 and Arg41*. Clearly, the dissociative characteristics of **TS2b** results in a shorter C6–O^−^ bond, which weakens these hydrogen bonds with Arg41/Arg41* and increases the energy barrier. In addition, heterolysis of the C6–N1 bond results in concentrated negative charge on N1(−0.76), which leads to electrostatic repulsion between it and the carboxylate group of Ser108 thus abolishes their hydrogen bond interaction. It also results in an energetically unfavorable geometric structure of **TS2b**. By crossing over the transition state **TS2b**, the reaction system reaches the product of **pathway 2** (**PROb**) and completes the catalytic cycle.

### 2.3. Hydrolysis Mechanism of 5-HIU Catalyzed by kp*HIUH* Based on Complex ***1***

The docking and MD simulation calculations indicated that the binding conformation of 5-HIU in **complex 1** is not as stable as that in **complex 2**. The spatial arrangement of the residues around the substrate in this complex does not allow the reaction to proceed in a mechanism as proposed by French et al. either [1], by which one of the Arg41 serves as the proton donor in the reaction. In **complex 1**, the substrate adopts distinct orientation from that in **complex 2**. In **complex 1** (Figure 2a), two histidine residues His7 and His92 from chain A are located on the two sides of the molecular plane of 5-HIU separately. Therefore, based on this complex, the lytic water can only form a hydrogen bond with His92 and thus the proposed reaction mechanism uses an intramolecular proton transfer mode in the second step (Scheme S1, pathway 3). We examined pathway 3 with DFT calculations. With only one histidine (His92) involved in the reaction, in this pathway, the first step needs to cross over a 11.7 kcal/mol energy barrier, while the energy barrier for the second step is 24.9 kcal/mol. Therefore, we can conclude that pathway 3 is not as competitive as pathway 1 in energy. The corresponding two transition states and intermediate along this pathway are depicted in Figures S7–S9. 

## 3. Materials and Methods

To investigate the possible binding conformations of 5-HIU to *kp*HIUH, molecular docking calculations were conducted based on crystal structure of *kp*HIUH (PDB code 3QVA) [1] by using the program AutoDock 4.2 [12]. A *kp*HIUH dimer which consists two intact active sites was extracted from the X-ray structure (PDB entry 3QVA) [1] to build the protein model. When performing the docking calculations, the *kp*HIUH protein was set to be rigid. While for the substrate 5-HIU, all the C-C, C-N single bonds were set to be rotatable to better fit in the binding pocket. A cubic grid with 108 × 90 × 108 grid points and a spacing of 0.24 Å was chosen to ensure complete coverage one of the active sites in the dimer. The docking simulation was performed with 500 cycles using Lamarckian genetic algorithm (LGA) [13]. The obtained complex structure was then subjected to a 100 ns molecular dynamics (MD) simulation to verify its stability. MD simulation was also performed for the complex structure in which 5-HIU adopts a conformation from the homologue superposition by French et al. [1]. All MD simulations were performed by using Amber20 program. Amber ff14SB [14] force field was used for the protein, while 5-HIU was parametrized with a general Amber force field (GAFF) using the ANTECHAMBER [15,16] module embedded in Amber20. As for the substrate 5-HIU, the atomic charges were generated by the AM1-BCC protocol by using ANTECHAMBER program [17].The two complexes were soaked into TIP3P [18] water box with a minimum distance of 10 Å to the protein boundary, by which 16049 water molecules were inserted in the system. The whole system was then neutralized with Na^+^ as counter ions. Prior to MD simulations, 1000-step steepest descent and 1000-step conjugated gradient optimizations were performed for the whole systems. The energy minimized systems were then heated to 300 K and equilibrated for 120 ps in the NPT ensemble with 300 K temperature and 1bar pressure. Product phase simulations were finally performed in the NPT ensemble for 100 ns using a Langevin thermostat and a Berendsen barostat at 300 K and 1 bar respectively [19,20]. Long-distance electrostatic interactions were treated by the particle-mesh Ewald (PME) method [21] with 8 Å cutoff. The SHAKE algorithm [22] was used to constrain the covalent bonds involving hydrogen atoms, allowing for a 2 fs time step. 

Since the *kp*HIUH/5-HIU complex displays very small structural fluctuation during MD simulation, the final snapshot was therefore extracted from the MD product phase trajectory to build the cluster model for the investigation of the catalysis mechanism of *kp*HIUH. The cluster model was built from a *kp*HIUH dimer of protein chain A and D. The cluster model contains 197 atoms with a net charge of -1 and consists of the substrate 5-HIU and the first shell residues, including His7-His7*, Arg41-Arg41*, His92-His92*, Tyr105-Tyr105*, and Ser108-Ser108* (asterisk signifies chain D). A water molecule which locates next to the C6 of 5-HIU was included in the cluster model as the lytic water. All amino acid residues were truncated at α-carbon. The boundary α-carbon atoms were captured by hydrogen atoms. To maintain the overall structure of the active site and mimic the protein environment, these boundary α-carbon atoms and the attached hydrogen atoms were fixed during geometry optimizations. 

The geometric structures of all stationary points, including reactant, intermediates and transition structures were optimized by using B3LYP functional with the 6-31G (D, P) basis set. Analytic vibrational frequency calculations were then performed for the optimized geometry structures to confirm that correct stationary points were located and to obtain the corresponding zero-point energies (ZPE) and entropy corrections. Electronic energies were then evaluated with larger basis sets by using the cc-pVTZ [23] for all atoms. Solvent effects were estimated by the SMD continuum solvation model with dielectric constant set to 4 to mimic the protein environment [24]. Van der Waals effect was assessed using Grimme’s D3(BJ) method [25]. All DFT calculations were performed by Gaussian09 [26]. 

## 4. Conclusions

In this study, by employing molecular docking, molecular dynamics simulation and DFT calculation, we have systematically investigated the binding conformation of 5-HIU in *kp*HIUH and its catalytic hydrolysis mechanism. Our calculations indicated that 5-HIU is bound in the active site of *kp*HIUH in a stable conformation through multiple hydrogen bonding interactions which are arranged in nearly two-fold symmetry around the substrate. The stable binding conformation of 5-HIU allows His92 from one protein chain to deprotonate the lytic water in order to initiate the hydrolysis reaction, while His7 assists to stabilize the lytic water in the reaction. The hydrolysis process was found to proceed via a two-step reaction. In the first step, the lytic water molecule is deprotonated by His92 and carries out nucleophilic attack on the C6 carbonyl of 5-HIU, resulting in an oxyanion intermediate. Subsequently, by accepting a proton transferred from His92, the C6–N1 bond cleaves and leads to ring opening, which completes the catalytic cycle. The reaction is rate-limited by the second reaction step with calculated energy barrier of 15.3 kcal/mol, which agrees well with the experiment results. Based on the obtained catalytic mechanism, we have also analyzed the roles of important residues in the active site, including His7, His92, Arg41 and Ser108. 

## Figures and Tables

**Table 1:** The occupancy of the hydrogen bond interactions between the residues in the active site of *kp*HIUH and the substrate 5-HIU in **complex 2**. Residue names followed by an asterisk (*) are from the adjoining chain.

Hydrogen bonds	His 7 *	His 92 *	Arg 41 *	Tyr 105	Tyr 105 *	Ser 108	Ser 108 *
Occupancy (%)	90.68	70.34	74.66	64.79	33.15	33.15	65.66

**Table 2:** The hydrogen bond lengths (Å) between *kp*HIUH and the substrate 5-HIU in the optimized **complex 2**. The distances are measured between the two heavy atoms in the hydrogen bonds.

Hydrogen Bonds	Distance (Å)
His7*……O12	2.73
His92*……O12	2.95
Arg41(N^ω^) ……O13	3.19
Arg41*(N^ω^) ……O13	2.91
Arg41*(N^ω1^) ……O13	2.96
Tyr 105……N7	2.78
Tyr 105*……N3	2.79
Ser 108……O10	3.05
Ser 108*……O11	2.80
Ser 108*……N1	3.58

**Table 3:** Some important bond lengths (Å) related to reaction coordinate in **la**, **TS1a** and **TS1b**.

	C6_HIU_–O_WAT_	C6–O13	C6–N1	C5–C6	O_WAT_–H1_WAT_	O_WAT_–H2_WAT_	N^ε^_His92_–H1_WAT_	N^δ^_His7_–H2_WAT_
1a	2.81	1.25	1.35	1.53	0.98	0.98	1.82	1.94
TS1a	1.54	1.33	1.46	1.55	1.28	1.00	1.20	1.83
TS1b	1.69	1.30	1.42	1.55	0.98	1.20	1.91	1.29

**Table 4:** The lengths of the hydrogen bonds between Arg41/Arg41* and C6=O in the reaction species.

Hydrogen Bonds	1a	TS1a	TS1b	INT1a	INT1b	TS2a	TS2b
O–N^ω^_Arg41_	3.19	2.83	2.80	2.82	2.78	2.91	2.94
O–N^ω1^_Arg41_	4.07	3.28	3.09	3.29	3.21	3.42	3.29
O–N^ω^_Arg41*_	2.91	2.89	2.88	2.88	2.89	2.81	2.97
O–N^ω1^_Arg41*_	2.96	2.94	3.42	2.91	2.87	2.79	3.08

## Data Availability

The data presented in this study are available on request from the corresponding author.

## Supplementary Materials

The following are available online, Figure S1: Structure of *kp*HIUH. (a) the 222 symmetry structure of *kp*HIUH, (b) the 2-fold symmetry active site embed in the *kp*HIUH tetramer., Figure S2. The best docking pose of 5-HIU to kpHIUH. Figure S3. The RMSD (Å) of kpHIUH/5-HIU complex during 100 ns simulation in **complex 1**. Figure S4. The RMSD (Å) of kpHIUH/5-HIU complex during 100 ns simulation in **complex 2**. Figure S5. The geometry structure of TS1a and TS1b, some important bond lengths are labeled in the unit of Å. Scheme S1. The possible pathway for 5-HIU hydrolysis by kpHIUH based on complex. Figure S6. Free energy profile of the pathway 3. The relative energies are labeled in kcal/mol. Figure S7. The geometry structure of TS1c, some important bond lengths are labeled in the unit of Å. Figure S8. The geometry structure of INT1c, some important bond lengths are labeled in the unit of Å. Figure S9. The geometry structure of TS2c, some important bond lengths are labeled in the unit of Å. Table S1: The energy data of the reaction species of the three possible reaction pathway (a.u.). Table S2. The average distance of hydrogen bonding interactions between 5-HIU and kpHIUH during the last 50 ns MD simulation (the hydrogen bonding lengthes were measured between two heavy atoms).

## Sample Availability

Not available.
