# Supplementary material for: Theoretical Studies on the Binding Mode and Reaction Mechanism of TLP Hydrolase kpHIUH
Source: Molecules. 2021 Jun 25;26(13):3884. doi: 10.3390/molecules26133884 (PMC8272043; doi:10.3390/molecules26133884)
Supplement: Supplementary file 1 [file molecules-26-03884-s001.zip › molecules-1203815-supplementary.pdf]

Supplementary Material

Theoretical Studies on the Binding Mode and Reaction Mechanism of TLP Hydrolase *kp*HIUH

Xixi Wang, Jiankai Shan, Wei Liu, Jing Li, Hongwei Tan *, Xichen Li * and Guangju Chen

Key Laboratory of Theoretical and Computational Photochemistry, Ministry of Education, College of Chemistry, Beijing Normal University, Beijing 100875, China; chemxixi@outlook.com (X.W.); jks@mail.bnu.edu.cn (J.S.); 201731150027@mail.bnu.edu.cn (W.L.); 201821150059@mail.bnu.edu.cn (J.L.); gjchen@bnu.edu.cn (G.C.)

* Correspondence: hongwei.tan@bnu.edu.cn (H.T.); xcli@bnu.edu.cn (X.L.)

Supplementary Figures

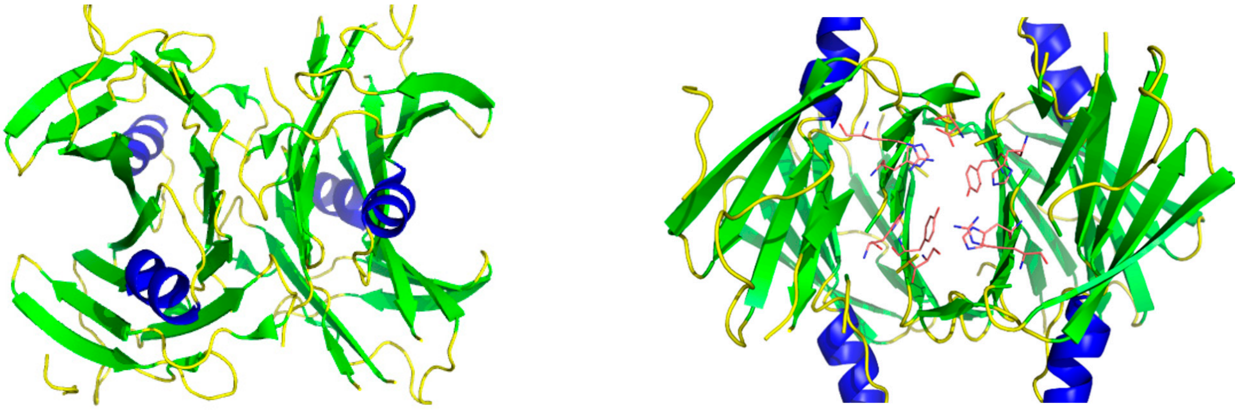

(a) (b)

Figure S1. Structure of *kp*HIUH. (**a**) the 222 symmetry structure of *kp*HIUH, (b) the 2-fold symmetry active site embed in the *kp*HIUH tetramer.

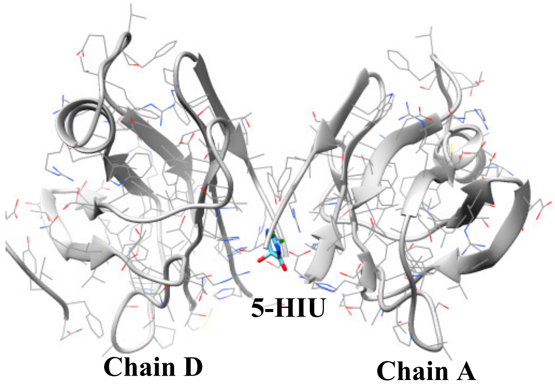

Figure S2. The best docking pose of 5-HIU to *kp*HIUH.

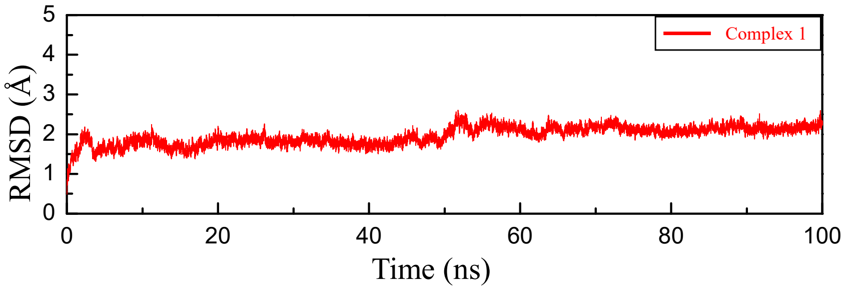

Figure S3. The RMSD (Å) of *kp*HIUH/5-HIU complex during 100ns simulation in **complex 1**.

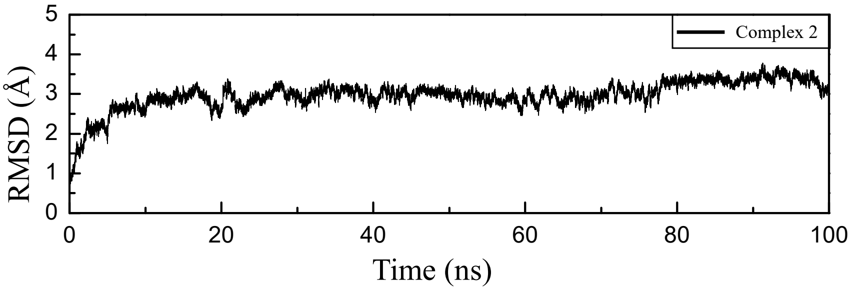

Figure S4. The RMSD (Å) of *kp*HIUH/5-HIU complex during 100ns simulation in **complex 2**.

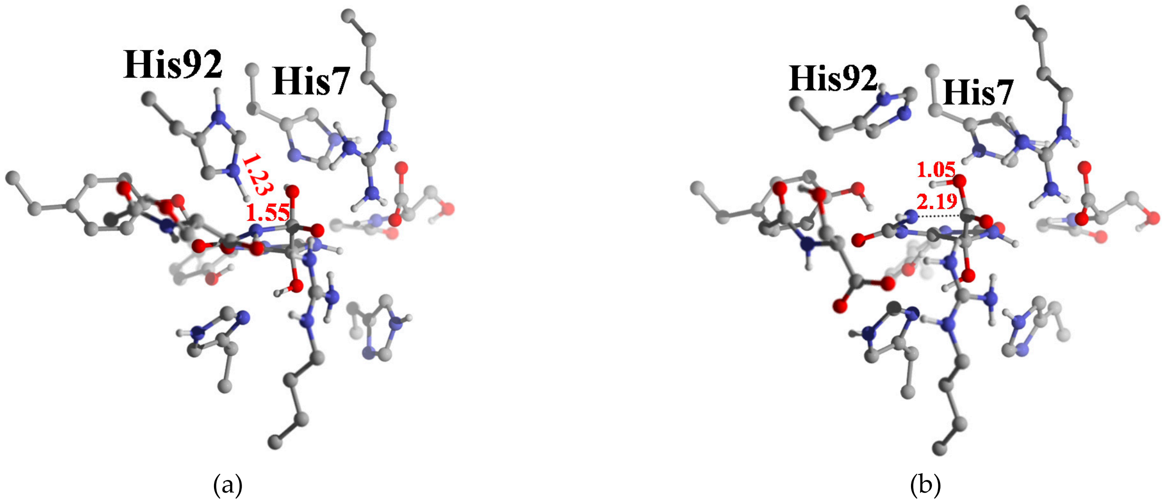

Figure S5. The geometry structure of TS1a and TS1b, some important bond lengths are labeled in the unit of Å.

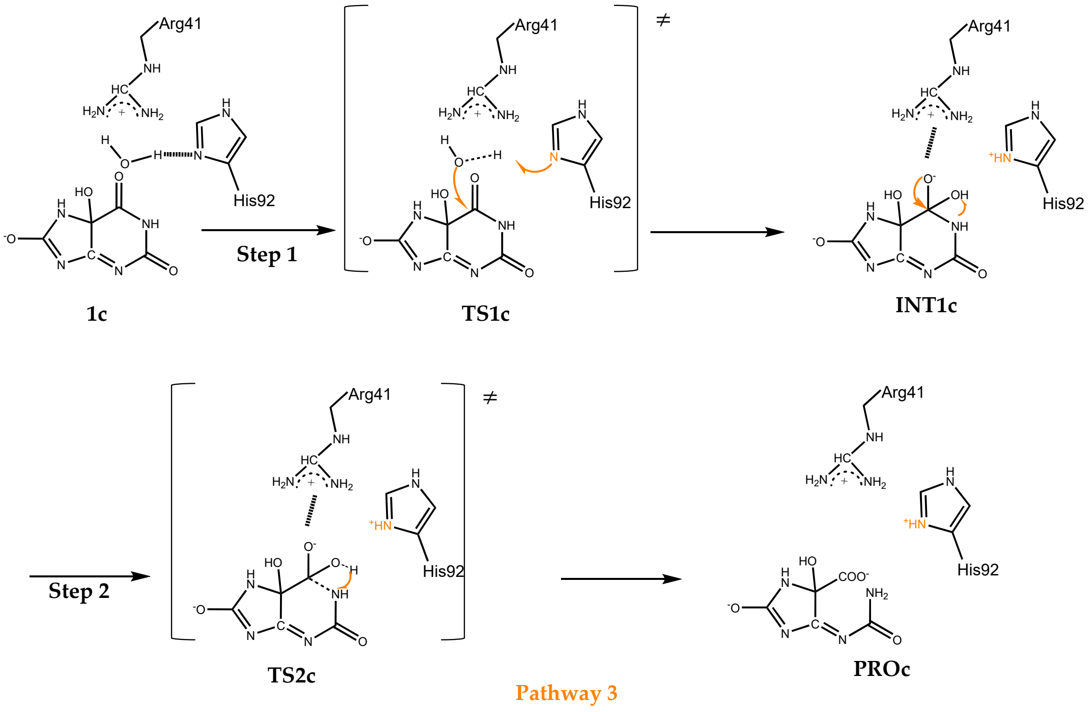

Scheme S1. The possible pathway for 5-HIU hydrolysis by *kp*HIUH based on **complex 1**.

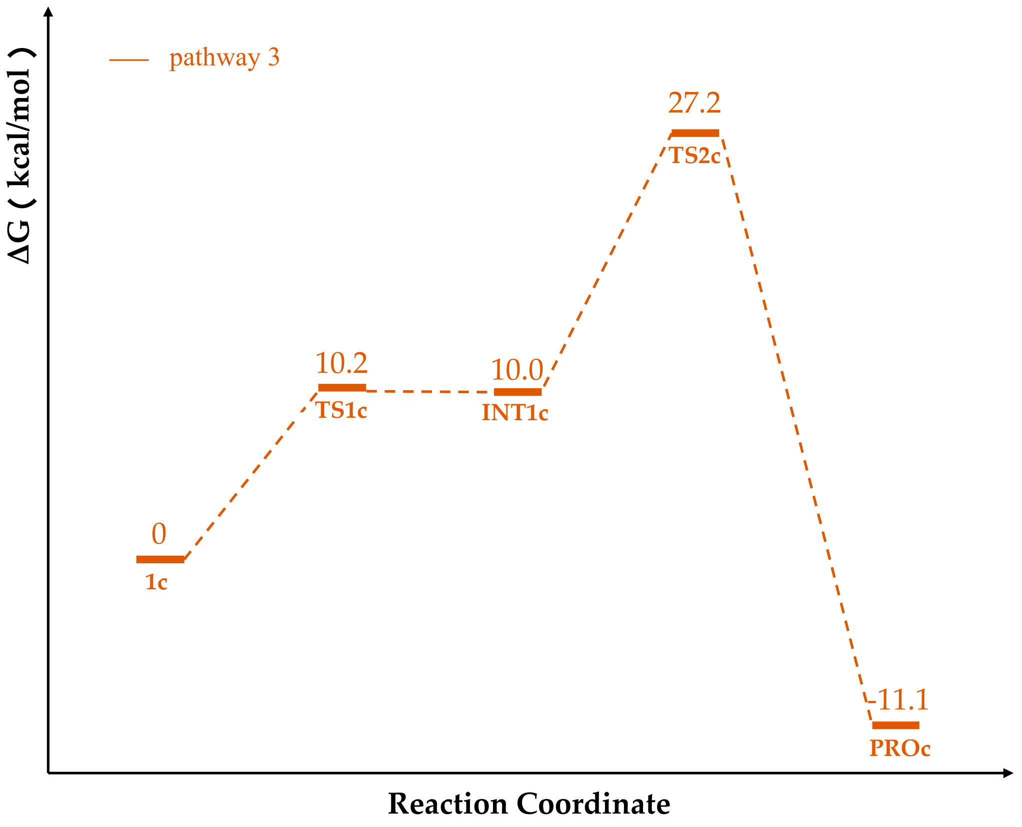

Figure S6. Free energy profile of the **pathway 3**. The relative energies are labeled in kcal/mol.

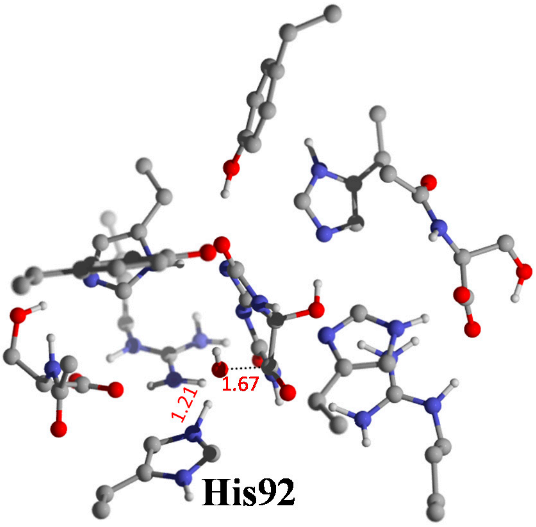

Figure S7. The geometry structure of **TS1c**, some important bond lengths are labeled in the unit of Å.

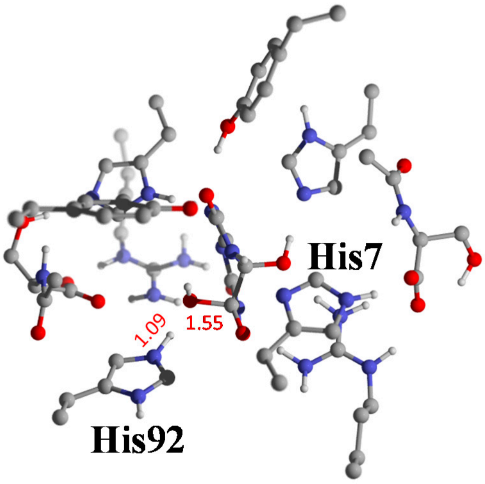

Figure S8. The geometry structure of **INT1c**, some important bond lengths are labeled in the unit of Å.

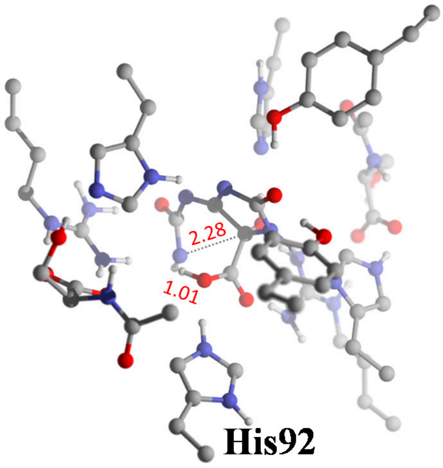

Figure S9. The geometry structure of **TS2c**, some important bond lengths are labeled in the unit of Å.

Supplementary table.

Table S1. The energy data of the reaction species of the three possible reaction pathway (a.u.).

	Electronic Energy (B3LYP/6-31G**)	Electronic Energy (cc-pVTZ)	Dispersion correction	ZPE	Solvation energy correction	Free energy correction	Corrected free energy
1a	−4609.22852	−4610.79977	−0.42810	1.28707	−0.14766	1.23701	−4608.85145
TS1a	−4609.19564	−4610.75678	−0.44133	1.28398	−0.15935	1.23220	−4608.84128
INT1a	−4609.20142	−4610.76448	−0.43547	1.28592	−0.16287	1.23439	−4608.84250
TS2a	−4609.17005	−4610.73973	−0.44347	1.28461	−0.15953	1.23104	−4608.82708
Proa	−4609.19692	−4610.76676	−0.43193	1.27481	−0.15934	1.22221	−4608.86101
TS1b	−4609.20050	−4610.76337	−0.43865	1.28370	−0.15219	1.23008	−4608.84042
INT1b	−4609.20826	−4610.77867	−0.43830	1.28877	−0.15258	1.23988	−4608.84091
TS2b	−4609.16653	−4610.73194	−0.43757	1.28354	−0.15135	1.22954	−4608.80778
Prob	−4609.22703	−4610.79456	−0.44612	1.28761	−0.14612	1.23499	−4608.86419
1c	−4609.24813	−4610.82118	−0.41511	1.49598	−0.14417	1.44888	−4608.43560
TS1c	−4609.22501	−4610.79239	−0.41581	1.49433	−0.14482	1.44176	−4608.41693
INT1c	−4609.22606	−4610.79363	−0.41708	1.49736	−0.14825	1.44508	−4608.41653
TS2c	−4609.19553	−4610.76361	−0.42028	1.49637	−0.15192	1.44358	−4608.39585
Proc	−4609.25481	−4610.82808	−0.41994	1.49791	−0.14491	1.44174	−4608.45328

Table S2. The average distance of hydrogen bonding interactions between 5-HIU and kpHIUH during the last 50ns MD simulation (the hydrogen bonding lengthes were measured between two heavy atoms).

Hydrogen bonding	Distance (Å)
His7*……O12	2.84
His92*……O12	2.96
Arg41*（N^{ω}）……O13	4.09
Arg41*（$N^{\omega 1}$）……O13	3.16
Tyr 105……N7	3.24
Tyr 105*……N3	3.20
Ser 108……N9	3.03
Ser 108……O10	4.03
Ser 108*……O11	4.51
Ser 108*……N1	3.03

Supplementary coordinates

1a

C	27.475	28.332	31.202
H	27.366	27.297	30.953
C	27.728	28.511	32.709
H	28.582	27.904	33.025
H	28.031	29.547	32.908
C	26.551	28.179	33.576
N	26.721	27.708	34.867
C	25.516	27.604	35.402
H	25.283	27.305	36.419

N	24.569	27.968	34.503
H	23.580	28.022	34.688
C	25.209	28.339	33.333
H	24.665	28.690	32.471
C	25.497	24.195	31.031
H	26.390	24.720	30.766
C	25.702	23.351	32.311
H	24.894	22.609	32.378
H	26.634	22.774	32.239
C	25.699	24.181	33.609
H	26.509	24.921	33.598
H	24.769	24.761	33.669
C	25.806	23.291	34.862
H	25.034	22.515	34.818
H	26.768	22.767	34.881
N	25.641	23.979	36.147
H	24.714	24.045	36.547
C	26.640	24.372	36.965
N	27.912	24.414	36.499
H	28.024	24.628	35.519
H	28.565	24.926	37.093
N	26.389	24.650	38.235
H	25.400	24.796	38.553
H	27.109	25.197	38.712
C	35.188	27.597	31.880
H	34.483	27.363	31.109
C	34.496	27.525	33.256
H	35.120	26.976	33.967
H	34.411	28.540	33.665
C	33.111	26.940	33.256
N	32.300	26.911	32.136
C	31.125	26.501	32.568
H	30.244	26.363	31.955
N	31.135	26.229	33.906
H	30.331	26.211	34.524
C	32.407	26.518	34.357
H	32.697	26.394	35.390
C	34.818	35.663	31.905
H	35.371	36.567	31.750
C	34.090	35.789	33.262
H	34.838	35.843	34.063
H	33.565	36.754	33.277
C	33.103	34.686	33.572
C	33.350	33.731	34.565
H	34.290	33.768	35.114
C	32.423	32.739	34.882
H	32.633	32.007	35.658
C	31.198	32.683	34.198
O	30.245	31.767	34.466
H	30.477	31.252	35.279
C	30.943	33.623	33.186
H	29.991	33.576	32.666
C	31.881	34.604	32.887
H	31.652	35.336	32.115
C	36.057	30.045	35.668
H	36.505	30.520	36.516
H	34.996	30.171	35.708

C	36.405	28.568	35.852
O	37.459	28.098	35.407
N	35.523	27.900	36.647
H	34.592	28.310	36.740
C	35.632	26.485	37.070
H	35.399	25.829	36.221
C	37.026	26.086	37.597
H	37.403	26.897	38.241
H	36.894	25.212	38.241
O	37.939	25.720	36.586
H	37.970	26.495	35.989
C	34.538	26.257	38.155
O	33.362	26.025	37.746
O	34.901	26.330	39.364
C	31.541	32.283	42.545
H	31.752	31.477	43.218
C	31.392	31.833	41.073
H	30.502	31.203	40.990
H	31.186	32.720	40.464
C	32.551	31.095	40.476
N	32.657	29.715	40.580
C	33.754	29.377	39.923
H	34.145	28.376	39.788
N	34.370	30.473	39.415
H	35.157	30.450	38.785
C	33.608	31.576	39.745
H	33.865	32.572	39.418
C	33.624	29.523	45.334
H	33.361	30.228	44.574
C	33.300	28.093	44.847
H	33.744	27.364	45.540
H	32.214	27.926	44.901
C	33.774	27.811	43.409
H	33.209	28.438	42.710
H	34.822	28.114	43.296
C	33.628	26.318	42.999
H	34.573	25.790	43.152
H	32.903	25.819	43.654
N	33.232	26.068	41.615
H	33.942	26.018	40.859
C	31.969	26.214	41.187
N	30.959	26.556	42.040
H	31.271	26.958	42.913
H	30.292	27.176	41.548
N	31.655	25.970	39.920
H	32.381	25.891	39.176
H	30.713	26.200	39.616
C	24.032	31.459	42.887
H	24.803	31.665	43.600
C	24.546	30.380	41.887
H	23.959	29.460	41.993
H	24.400	30.706	40.856
C	25.996	30.075	42.061
N	26.479	29.376	43.157
C	27.835	29.310	43.051
H	28.456	28.799	43.773
N	28.253	29.930	41.967

C	27.117	30.410	41.343
H	27.176	30.969	40.419
C	24.062	37.649	37.528
H	23.759	38.612	37.172
C	24.742	36.851	36.388
H	23.983	36.457	35.700
H	25.348	37.551	35.796
C	25.633	35.721	36.870
C	25.391	34.381	36.549
H	24.518	34.128	35.949
C	26.239	33.354	36.970
H	26.028	32.317	36.728
C	27.376	33.656	37.735
O	28.246	32.721	38.185
H	28.196	31.882	37.660
C	27.627	34.995	38.075
H	28.514	35.221	38.657
C	26.771	36.001	37.644
H	27.004	37.033	37.901
C	22.425	31.108	38.385
H	21.606	31.057	37.699
H	23.346	31.024	37.849
C	22.199	29.904	39.303
O	21.287	29.917	40.143
N	22.967	28.823	39.027
H	23.822	28.933	38.481
C	22.866	27.490	39.638
H	23.156	27.537	40.700
C	21.456	26.875	39.610
H	21.014	27.060	38.614
H	21.578	25.795	39.723
O	20.622	27.320	40.658
H	20.675	28.297	40.635
C	23.934	26.590	38.941
O	23.813	25.337	39.016
O	24.878	27.205	38.369
O	28.755	26.303	38.623
C	28.630	27.437	38.123
N	27.500	28.174	38.225
H	26.653	27.757	38.627
C	27.266	29.482	37.667
O	26.139	29.949	37.801
N	28.300	30.154	37.089
C	29.455	29.522	36.938
N	30.611	30.084	36.643
C	31.595	29.109	36.837
O	32.803	29.280	36.694
N	31.008	27.922	37.288
H	31.564	27.073	37.291
C	29.604	27.996	37.087
O	29.223	27.289	35.890
H	28.293	27.526	35.591
H	30.601	32.724	42.803
H	32.313	33.020	42.618
H	34.673	29.585	45.532
H	33.078	29.743	46.229
H	23.796	32.354	42.350

H	23.160	31.107	43.395
H	34.106	35.542	31.116
H	35.491	34.831	31.909
H	35.559	28.590	31.733
H	36.001	26.903	31.843
H	25.201	23.563	30.219
H	24.717	24.898	31.237
H	28.320	28.736	30.684
H	26.593	28.863	30.915
H	22.398	32.045	38.903
H	36.436	30.487	34.771
O	30.006	28.519	40.325
H	30.915	28.891	40.344
H	29.458	29.140	40.855
H	24.781	37.769	38.311
H	23.208	37.125	37.906
H	25.917	28.965	43.886

TS1a

C	27.476	28.332	31.202
H	27.366	27.297	30.953
C	27.741	28.500	32.708
H	28.600	27.894	33.009
H	28.038	29.537	32.913
C	26.576	28.144	33.579
N	26.757	27.547	34.815
C	25.563	27.462	35.376
H	25.341	27.113	36.377
N	24.611	27.947	34.542
H	23.636	28.055	34.773
C	25.238	28.393	33.392
H	24.693	28.850	32.582
C	25.497	24.195	31.031
H	26.390	24.720	30.766
C	25.718	23.351	32.313
H	24.917	22.602	32.389
H	26.653	22.782	32.226
C	25.729	24.180	33.614
H	26.495	24.963	33.568
H	24.772	24.707	33.717
C	25.946	23.301	34.864
H	25.237	22.465	34.843
H	26.945	22.853	34.849
N	25.771	23.969	36.159
H	24.848	23.987	36.570
C	26.759	24.448	36.949
N	28.022	24.552	36.479
H	28.108	24.727	35.489
H	28.608	25.185	37.043
N	26.518	24.764	38.214
H	25.541	24.873	38.558
H	27.243	25.359	38.640
C	35.188	27.597	31.880
H	34.483	27.363	31.109
C	34.489	27.530	33.252
H	35.140	27.053	33.991
H	34.324	28.550	33.622

C	33.148	26.853	33.263
N	32.296	26.864	32.173
C	31.155	26.380	32.617
H	30.255	26.251	32.031
N	31.227	26.013	33.932
H	30.440	25.962	34.570
C	32.505	26.325	34.355
H	32.841	26.150	35.367
C	34.818	35.663	31.905
H	35.371	36.567	31.750
C	34.075	35.790	33.252
H	34.807	35.797	34.070
H	33.582	36.772	33.279
C	33.046	34.712	33.499
C	33.294	33.638	34.360
H	34.253	33.578	34.873
C	32.347	32.642	34.586
H	32.561	31.814	35.255
C	31.098	32.706	33.943
O	30.132	31.786	34.132
H	30.383	31.189	34.887
C	30.839	33.772	33.067
H	29.868	33.818	32.582
C	31.800	34.753	32.856
H	31.573	35.582	32.186
C	36.057	30.044	35.668
H	36.505	30.520	36.516
H	34.996	30.171	35.708
C	36.455	28.591	35.886
O	37.550	28.155	35.510
N	35.576	27.909	36.672
H	34.619	28.266	36.674
C	35.772	26.548	37.215
H	35.648	25.804	36.418
C	37.157	26.318	37.853
H	37.427	27.212	38.440
H	37.057	25.495	38.567
O	38.157	25.948	36.931
H	38.149	26.662	36.260
C	34.628	26.334	38.241
O	33.500	26.011	37.757
O	34.875	26.531	39.465
C	31.540	32.283	42.545
H	31.752	31.477	43.218
C	31.388	31.787	41.089
H	30.398	31.339	40.958
H	31.386	32.656	40.418
C	32.378	30.770	40.600
N	31.944	29.715	39.812
C	33.018	29.021	39.495
H	33.032	28.137	38.883
N	34.130	29.561	40.042
H	35.047	29.145	39.954
C	33.744	30.682	40.754
H	34.454	31.309	41.270
C	33.624	29.523	45.334
H	33.361	30.228	44.574

C	33.231	28.105	44.826
H	33.856	27.346	45.316
H	32.202	27.885	45.142
C	33.304	27.932	43.294
H	32.613	28.634	42.812
H	34.301	28.201	42.925
C	32.970	26.494	42.840
H	33.782	25.822	43.141
H	32.074	26.135	43.359
N	32.763	26.319	41.403
H	33.582	26.310	40.764
C	31.567	26.159	40.811
N	30.400	26.330	41.514
H	30.430	27.052	42.220
H	29.600	26.426	40.889
N	31.469	25.768	39.553
H	32.314	25.729	38.940
H	30.543	25.901	39.104
C	24.032	31.460	42.887
H	24.803	31.665	43.600
C	24.457	30.303	41.963
H	24.138	29.356	42.420
H	23.929	30.354	41.013
C	25.912	30.196	41.673
N	26.893	30.190	42.657
C	28.085	29.915	42.084
H	29.024	29.852	42.611
N	27.906	29.750	40.784
C	26.568	29.926	40.505
H	26.176	29.847	39.499
C	24.061	37.649	37.528
H	23.759	38.612	37.172
C	24.723	36.863	36.367
H	23.947	36.491	35.686
H	25.321	37.571	35.777
C	25.620	35.713	36.788
C	25.375	34.388	36.406
H	24.480	34.161	35.829
C	26.249	33.347	36.727
H	26.037	32.323	36.435
C	27.423	33.618	37.449
O	28.330	32.678	37.787
H	28.238	31.843	37.256
C	27.671	34.938	37.861
H	28.584	35.143	38.413
C	26.786	35.959	37.531
H	27.023	36.977	37.835
C	22.425	31.108	38.386
H	21.606	31.057	37.699
H	23.346	31.024	37.849
C	22.202	29.913	39.311
O	21.329	29.946	40.193
N	22.950	28.825	39.022
H	23.753	28.897	38.396
C	22.887	27.505	39.667
H	23.187	27.589	40.723
C	21.493	26.858	39.671

H	21.041	26.993	38.671
H	21.641	25.786	39.826
O	20.654	27.327	40.706
H	20.706	28.304	40.667
C	23.974	26.620	38.971
O	23.911	25.371	39.113
O	24.858	27.259	38.337
O	28.873	26.385	38.619
C	28.893	27.653	38.233
N	27.571	28.214	37.999
H	26.751	27.717	38.344
C	27.309	29.418	37.356
O	26.164	29.898	37.398
N	28.342	30.099	36.738
C	29.498	29.478	36.606
N	30.631	30.032	36.187
C	31.649	29.123	36.437
O	32.849	29.293	36.224
N	31.132	27.981	37.071
H	31.687	27.134	37.041
C	29.707	27.989	36.956
O	29.281	27.129	35.903
H	28.353	27.349	35.605
H	30.601	32.724	42.803
H	32.313	33.020	42.618
H	34.673	29.585	45.532
H	33.078	29.743	46.229
H	23.796	32.354	42.350
H	23.160	31.107	43.395
H	34.106	35.542	31.116
H	35.491	34.831	31.909
H	35.559	28.590	31.733
H	36.001	26.903	31.843
H	25.201	23.563	30.219
H	24.717	24.898	31.237
H	28.320	28.736	30.684
H	26.593	28.863	30.915
H	22.398	32.045	38.903
H	36.436	30.487	34.771
O	29.499	28.505	39.363
H	30.402	28.917	39.244
H	28.693	29.243	40.039
H	24.781	37.769	38.311
H	23.208	37.125	37.906
H	26.746	30.369	43.639

INT1a

C	27.475	28.332	31.202
H	27.366	27.297	30.953
C	27.743	28.498	32.707
H	28.603	27.891	33.007
H	28.040	29.535	32.914
C	26.581	28.141	33.581
N	26.766	27.529	34.810
C	25.574	27.448	35.378
H	25.357	27.093	36.378
N	24.621	27.948	34.555

H	23.648	28.062	34.795
C	25.244	28.401	33.406
H	24.696	28.872	32.605
C	25.497	24.195	31.031
H	26.390	24.720	30.766
C	25.719	23.350	32.311
H	24.917	22.602	32.388
H	26.653	22.779	32.222
C	25.734	24.176	33.614
H	26.500	24.958	33.568
H	24.778	24.705	33.719
C	25.951	23.295	34.862
H	25.240	22.461	34.841
H	26.949	22.844	34.844
N	25.782	23.962	36.158
H	24.860	23.984	36.571
C	26.774	24.438	36.945
N	28.036	24.538	36.474
H	28.123	24.709	35.484
H	28.624	25.171	37.041
N	26.536	24.758	38.210
H	25.560	24.872	38.554
H	27.267	25.348	38.632
C	35.188	27.597	31.880
H	34.483	27.363	31.109
C	34.484	27.528	33.250
H	35.139	27.070	33.996
H	34.297	28.548	33.610
C	33.157	26.826	33.257
N	32.300	26.840	32.169
C	31.170	26.329	32.609
H	30.270	26.196	32.024
N	31.253	25.941	33.917
H	30.468	25.877	34.556
C	32.527	26.269	34.342
H	32.868	26.084	35.350
C	34.818	35.663	31.905
H	35.371	36.567	31.750
C	34.075	35.791	33.252
H	34.807	35.798	34.069
H	33.583	36.774	33.278
C	33.045	34.714	33.497
C	33.299	33.630	34.344
H	34.261	33.563	34.849
C	32.352	32.633	34.565
H	32.570	31.797	35.223
C	31.099	32.705	33.932
O	30.134	31.783	34.118
H	30.393	31.175	34.861
C	30.833	33.782	33.071
H	29.859	33.835	32.595
C	31.794	34.765	32.865
H	31.564	35.602	32.206
C	36.057	30.044	35.668
H	36.505	30.520	36.516
H	34.996	30.171	35.708
C	36.465	28.594	35.885

O	37.566	28.169	35.512
N	35.589	27.903	36.667
H	34.629	28.251	36.661
C	35.797	26.545	37.212
H	35.681	25.798	36.416
C	37.182	26.329	37.854
H	37.443	27.227	38.440
H	37.087	25.506	38.569
O	38.188	25.967	36.936
H	38.175	26.680	36.264
C	34.652	26.324	38.236
O	33.526	26.001	37.747
O	34.896	26.519	39.460
C	31.540	32.283	42.546
H	31.752	31.477	43.218
C	31.401	31.787	41.087
H	30.416	31.332	40.947
H	31.396	32.659	40.419
C	32.403	30.780	40.601
N	31.983	29.726	39.805
C	33.066	29.043	39.491
H	33.090	28.162	38.876
N	34.170	29.591	40.047
H	35.092	29.187	39.963
C	33.768	30.705	40.763
H	34.471	31.335	41.286
C	33.624	29.523	45.334
H	33.361	30.228	44.574
C	33.233	28.105	44.822
H	33.856	27.346	45.313
H	32.202	27.885	45.135
C	33.311	27.935	43.290
H	32.619	28.636	42.807
H	34.308	28.207	42.925
C	32.981	26.497	42.831
H	33.794	25.826	43.134
H	32.084	26.135	43.346
N	32.780	26.325	41.393
H	33.602	26.312	40.757
C	31.587	26.158	40.797
N	30.417	26.324	41.497
H	30.443	27.051	42.198
H	29.621	26.419	40.866
N	31.494	25.765	39.540
H	32.341	25.726	38.929
H	30.567	25.888	39.087
C	24.098	31.417	42.824
H	24.869	31.622	43.538
C	24.519	30.257	41.908
H	24.209	29.312	42.375
H	23.988	30.296	40.959
C	25.972	30.163	41.616
N	26.954	30.163	42.601
C	28.148	29.906	42.029
H	29.090	29.847	42.550
N	27.963	29.751	40.728
C	26.625	29.911	40.445

H	26.234	29.833	39.437
C	24.061	37.649	37.529
H	23.759	38.612	37.172
C	24.723	36.859	36.369
H	23.948	36.495	35.684
H	25.329	37.564	35.783
C	25.611	35.701	36.791
C	25.369	34.382	36.386
H	24.481	34.165	35.794
C	26.237	33.334	36.702
H	26.028	32.315	36.392
C	27.403	33.592	37.443
O	28.306	32.646	37.778
H	28.229	31.821	37.228
C	27.646	34.906	37.878
H	28.552	35.100	38.444
C	26.768	35.933	37.552
H	27.004	36.946	37.875
C	22.425	31.108	38.386
H	21.606	31.057	37.699
H	23.346	31.024	37.849
C	22.204	29.910	39.308
O	21.334	29.940	40.193
N	22.951	28.823	39.014
H	23.755	28.897	38.390
C	22.890	27.502	39.657
H	23.179	27.589	40.717
C	21.500	26.849	39.649
H	21.055	26.983	38.646
H	21.650	25.777	39.803
O	20.650	27.313	40.678
H	20.703	28.290	40.646
C	23.989	26.623	38.971
O	23.926	25.374	39.104
O	24.881	27.270	38.354
O	28.911	26.351	38.573
C	28.941	27.628	38.199
N	27.611	28.191	37.970
H	26.793	27.694	38.319
C	27.346	29.392	37.326
O	26.201	29.875	37.376
N	28.372	30.073	36.700
C	29.529	29.452	36.564
N	30.659	30.008	36.143
C	31.680	29.101	36.392
O	32.880	29.276	36.182
N	31.167	27.958	37.024
H	31.726	27.113	37.002
C	29.740	27.961	36.912
O	29.311	27.104	35.860
H	28.378	27.320	35.577
H	30.601	32.724	42.803
H	32.313	33.020	42.618
H	34.673	29.585	45.532
H	33.078	29.743	46.229
H	23.862	32.312	42.287
H	23.225	31.065	43.333

H	34.106	35.542	31.116
H	35.491	34.831	31.909
H	35.559	28.590	31.733
H	36.001	26.903	31.843
H	25.201	23.563	30.219
H	24.717	24.898	31.237
H	28.320	28.736	30.684
H	26.593	28.863	30.915
H	22.398	32.045	38.903
H	36.436	30.487	34.771
O	29.549	28.457	39.309
H	30.447	28.855	39.164
H	28.714	29.279	40.016
H	24.781	37.769	38.311
H	23.208	37.125	37.906
H	26.805	30.333	43.583

TS2a

C	27.475	28.332	31.202
H	27.366	27.297	30.953
C	27.726	28.489	32.709
H	28.576	27.874	33.023
H	28.014	29.524	32.933
C	26.537	28.112	33.535
N	26.668	27.405	34.719
C	25.452	27.297	35.227
H	25.193	26.885	36.192
N	24.538	27.877	34.412
H	23.557	27.982	34.618
C	25.212	28.408	33.328
H	24.703	28.943	32.543
C	25.497	24.195	31.031
H	26.390	24.720	30.766
C	25.712	23.327	32.293
H	24.900	22.588	32.357
H	26.638	22.745	32.190
C	25.743	24.127	33.606
H	26.535	24.884	33.582
H	24.805	24.687	33.713
C	25.916	23.218	34.838
H	25.154	22.430	34.816
H	26.884	22.706	34.803
N	25.803	23.880	36.138
H	24.893	23.952	36.574
C	26.829	24.375	36.865
N	28.080	24.426	36.361
H	28.162	24.534	35.361
H	28.693	25.076	36.893
N	26.624	24.764	38.119
H	25.656	24.895	38.469
H	27.384	25.341	38.506
C	35.187	27.598	31.880
H	34.483	27.363	31.109
C	34.437	27.473	33.215
H	35.120	27.602	34.056
H	33.705	28.286	33.298
C	33.730	26.153	33.370

N	33.120	25.517	32.301
C	32.578	24.425	32.796
H	32.022	23.683	32.239
N	32.802	24.315	34.137
H	32.495	23.574	34.746
C	33.543	25.418	34.520
H	33.835	25.570	35.550
C	34.819	35.663	31.905
H	35.371	36.567	31.750
C	34.072	35.795	33.249
H	34.799	35.797	34.070
H	33.580	36.777	33.274
C	33.042	34.716	33.474
C	33.329	33.576	34.231
H	34.307	33.479	34.699
C	32.395	32.559	34.404
H	32.635	31.680	34.994
C	31.125	32.669	33.813
O	30.177	31.719	33.953
H	30.479	31.067	34.637
C	30.823	33.805	33.047
H	29.834	33.885	32.607
C	31.772	34.807	32.886
H	31.519	35.689	32.298
C	36.057	30.044	35.668
H	36.505	30.520	36.516
H	34.996	30.171	35.708
C	36.537	28.623	35.920
O	37.675	28.264	35.589
N	35.699	27.900	36.711
H	34.716	28.169	36.657
C	36.017	26.615	37.363
H	36.022	25.804	36.623
C	37.386	26.595	38.073
H	37.531	27.558	38.590
H	37.343	25.826	38.850
O	38.462	26.265	37.223
H	38.402	26.910	36.488
C	34.852	26.349	38.351
O	33.813	25.827	37.851
O	34.998	26.699	39.560
C	31.540	32.283	42.546
H	31.752	31.477	43.218
C	31.405	31.819	41.075
H	30.404	31.408	40.909
H	31.440	32.707	40.430
C	32.368	30.785	40.562
N	31.928	29.859	39.631
C	32.968	29.097	39.352
H	32.962	28.258	38.677
N	34.066	29.482	40.043
H	34.926	28.945	40.031
C	33.702	30.561	40.828
H	34.405	31.063	41.474
C	33.624	29.523	45.334
H	33.361	30.228	44.574
C	33.227	28.107	44.824

H	33.833	27.343	45.329
H	32.189	27.902	45.120
C	33.329	27.934	43.295
H	32.670	28.658	42.801
H	34.342	28.174	42.951
C	32.961	26.511	42.824
H	33.732	25.808	43.165
H	32.028	26.187	43.298
N	32.819	26.347	41.377
H	33.666	26.369	40.776
C	31.655	26.157	40.732
N	30.456	26.337	41.376
H	30.461	27.083	42.056
H	29.698	26.448	40.704
N	31.627	25.726	39.483
H	32.510	25.660	38.928
H	30.733	25.828	38.974
C	24.098	31.417	42.824
H	24.869	31.622	43.538
C	24.429	30.167	42.012
H	24.341	29.312	42.699
H	23.653	30.010	41.265
C	25.744	30.067	41.346
N	26.979	30.204	41.971
C	27.947	29.811	41.106
H	29.006	29.798	41.307
N	27.377	29.445	39.978
C	26.024	29.599	40.100
H	25.370	29.306	39.297
C	24.061	37.649	37.529
H	23.759	38.612	37.172
C	24.708	36.873	36.350
H	23.920	36.528	35.669
H	25.305	37.590	35.767
C	25.605	35.700	36.710
C	25.385	34.411	36.206
H	24.496	34.222	35.607
C	26.280	33.360	36.429
H	26.084	32.365	36.040
C	27.450	33.584	37.169
O	28.387	32.641	37.413
H	28.311	31.850	36.831
C	27.669	34.863	37.710
H	28.577	35.031	38.281
C	26.765	35.893	37.478
H	26.985	36.882	37.878
C	22.425	31.108	38.386
H	21.606	31.057	37.699
H	23.346	31.024	37.849
C	22.212	29.889	39.281
O	21.439	29.916	40.252
N	22.859	28.776	38.866
H	23.578	28.839	38.147
C	22.865	27.453	39.507
H	23.189	27.553	40.554
C	21.493	26.766	39.543
H	21.025	26.867	38.548

H	21.668	25.702	39.722
O	20.655	27.245	40.577
H	20.745	28.218	40.582
C	23.972	26.611	38.785
O	23.962	25.363	38.936
O	24.815	27.285	38.127
O	29.025	26.227	38.268
C	29.163	27.515	37.964
N	27.778	28.209	37.821
H	26.983	27.565	37.800
C	27.506	29.357	36.941
O	26.354	29.778	36.929
N	28.551	29.971	36.332
C	29.704	29.317	36.263
N	30.842	29.849	35.860
C	31.840	28.897	36.072
O	33.041	29.057	35.864
N	31.297	27.751	36.646
H	31.806	26.879	36.574
C	29.866	27.822	36.613
O	29.357	26.995	35.585
H	28.402	27.189	35.406
H	30.601	32.724	42.803
H	32.313	33.020	42.618
H	34.673	29.585	45.532
H	33.078	29.743	46.229
H	23.862	32.312	42.287
H	23.225	31.065	43.333
H	34.106	35.542	31.116
H	35.491	34.831	31.909
H	35.559	28.590	31.733
H	36.001	26.903	31.843
H	25.201	23.563	30.219
H	24.717	24.898	31.237
H	28.320	28.736	30.684
H	26.593	28.863	30.915
H	22.398	32.045	38.903
H	36.436	30.487	34.771
O	29.778	28.187	39.056
H	30.422	28.902	38.857
H	27.714	28.827	38.964
H	24.781	37.769	38.311
H	23.208	37.125	37.906
H	27.134	30.556	42.904

Proa

C	-1.995	-0.592	-6.062
H	-2.105	-1.626	-6.312
C	-1.691	-0.437	-4.563
H	-0.815	-1.042	-4.302
H	-1.401	0.599	-4.347
C	-2.818	-0.827	-3.663
N	-2.588	-1.361	-2.405
C	-3.772	-1.506	-1.835
H	-3.972	-1.827	-0.822
N	-4.759	-1.124	-2.680
H	-5.733	-1.052	-2.432

C	-4.170	-0.678	-3.849
H	-4.748	-0.292	-4.673
C	-3.974	-4.729	-6.234
H	-3.080	-4.203	-6.499
C	-3.773	-5.582	-4.959
H	-4.579	-6.328	-4.904
H	-2.839	-6.156	-5.032
C	-3.784	-4.765	-3.653
H	-2.986	-4.012	-3.665
H	-4.719	-4.197	-3.582
C	-3.658	-5.666	-2.409
H	-4.449	-6.423	-2.430
H	-2.710	-6.218	-2.430
N	-3.763	-4.991	-1.113
H	-4.662	-4.949	-0.651
C	-2.739	-4.430	-0.435
N	-1.512	-4.359	-1.003
H	-1.505	-4.336	-2.013
H	-0.898	-3.629	-0.622
N	-2.924	-4.018	0.809
H	-3.890	-3.914	1.190
H	-2.195	-3.426	1.205
C	5.716	-1.326	-5.385
H	5.012	-1.561	-6.156
C	4.967	-1.436	-4.046
H	5.655	-1.295	-3.210
H	4.234	-0.621	-3.975
C	4.259	-2.753	-3.866
N	3.646	-3.406	-4.923
C	3.110	-4.492	-4.411
H	2.557	-5.245	-4.957
N	3.338	-4.581	-3.069
H	3.053	-5.326	-2.452
C	4.078	-3.471	-2.705
H	4.377	-3.311	-1.678
C	5.347	6.739	-5.360
H	5.899	7.643	-5.515
C	4.607	6.857	-4.009
H	5.346	6.906	-3.199
H	4.079	7.821	-3.993
C	3.623	5.745	-3.729
C	3.893	4.741	-2.792
H	4.840	4.755	-2.256
C	2.979	3.725	-2.519
H	3.207	2.953	-1.790
C	1.743	3.696	-3.186
O	0.809	2.751	-2.959
H	1.046	2.221	-2.153
C	1.464	4.688	-4.140
H	0.504	4.664	-4.645
C	2.391	5.692	-4.398
H	2.145	6.464	-5.125
C	6.586	1.120	-1.596
H	7.033	1.595	-0.748
H	5.524	1.246	-1.557
C	7.046	-0.307	-1.359
O	8.142	-0.711	-1.766

N	6.241	-1.000	-0.505
H	5.272	-0.685	-0.464
C	6.570	-2.311	0.086
H	6.538	-3.092	-0.682
C	7.971	-2.369	0.737
H	8.156	-1.415	1.259
H	7.951	-3.148	1.505
O	9.000	-2.708	-0.162
H	8.911	-2.065	-0.895
C	5.462	-2.620	1.121
O	4.454	-3.251	0.686
O	5.621	-2.212	2.310
C	2.069	3.358	5.280
H	2.281	2.553	5.952
C	2.026	2.939	3.777
H	1.025	2.653	3.457
H	2.244	3.833	3.180
C	2.943	1.831	3.344
N	2.453	0.758	2.612
C	3.491	-0.006	2.319
H	3.453	-0.914	1.743
N	4.630	0.505	2.823
H	5.518	0.023	2.736
C	4.306	1.677	3.481
H	5.052	2.297	3.952
C	4.152	0.598	8.069
H	3.890	1.304	7.308
C	3.781	-0.825	7.581
H	4.371	-1.568	8.136
H	2.732	-1.033	7.835
C	3.958	-1.043	6.067
H	3.243	-0.417	5.520
H	4.953	-0.710	5.746
C	3.766	-2.519	5.667
H	4.632	-3.102	6.003
H	2.900	-2.943	6.187
N	3.601	-2.768	4.236
H	4.417	-2.667	3.597
C	2.433	-3.046	3.642
N	1.251	-2.954	4.325
H	1.215	-2.223	5.020
H	0.440	-2.926	3.709
N	2.409	-3.488	2.390
H	3.274	-3.474	1.781
H	1.507	-3.476	1.925
C	-5.372	2.493	5.558
H	-4.602	2.698	6.272
C	-4.846	1.457	4.547
H	-4.823	0.459	5.006
H	-5.524	1.382	3.698
C	-3.477	1.817	4.071
N	-2.393	1.685	4.919
C	-1.285	2.114	4.260
H	-0.310	2.089	4.721
N	-1.571	2.513	3.042
C	-2.939	2.344	2.919
H	-3.448	2.590	1.998

C	-5.409	8.725	0.263
H	-5.711	9.688	-0.093
C	-4.773	7.968	-0.936
H	-5.573	7.539	-1.552
H	-4.282	8.715	-1.575
C	-3.754	6.892	-0.616
C	-3.970	5.540	-0.909
H	-4.931	5.234	-1.318
C	-2.982	4.568	-0.722
H	-3.173	3.528	-0.971
C	-1.721	4.941	-0.227
O	-0.694	4.090	-0.047
H	-0.903	3.161	-0.309
C	-1.498	6.292	0.097
H	-0.521	6.573	0.477
C	-2.494	7.239	-0.099
H	-2.281	8.282	0.129
C	-7.046	2.184	1.120
H	-7.865	2.132	0.433
H	-6.124	2.099	0.583
C	-7.297	0.993	2.044
O	-8.155	1.040	2.938
N	-6.592	-0.112	1.722
H	-5.818	-0.046	1.063
C	-6.626	-1.430	2.367
H	-6.298	-1.342	3.414
C	-8.013	-2.089	2.402
H	-8.475	-1.983	1.404
H	-7.858	-3.156	2.584
O	-8.843	-1.595	3.431
H	-8.777	-0.619	3.390
C	-5.550	-2.289	1.631
O	-5.555	-3.540	1.774
O	-4.715	-1.630	0.950
O	-0.389	-2.627	1.562
C	0.023	-1.476	1.346
N	-2.249	-0.148	1.065
H	-3.191	-0.461	1.295
C	-2.234	0.775	0.024
O	-3.277	1.167	-0.503
N	-0.994	1.252	-0.380
C	0.112	0.555	-0.391
N	1.276	1.088	-0.799
C	2.270	0.151	-0.725
O	3.459	0.291	-1.020
N	1.775	-1.052	-0.182
H	2.197	-1.907	-0.525
C	0.328	-0.988	-0.090
O	-0.263	-1.832	-1.033
H	-1.097	-1.479	-1.468
H	1.129	3.800	5.537
H	2.841	4.095	5.352
H	5.202	0.661	8.266
H	3.607	0.818	8.963
H	-5.609	3.388	5.022
H	-6.245	2.141	6.067
H	4.634	6.617	-6.149

H	6.019	5.907	-5.355
H	6.088	-0.333	-5.532
H	6.529	-2.020	-5.422
H	-4.269	-5.361	-7.046
H	-4.754	-4.026	-6.028
H	-1.151	-0.188	-6.580
H	-2.878	-0.060	-6.350
H	-7.073	3.121	1.637
H	6.964	1.563	-2.494
O	0.351	-0.716	2.370
H	1.074	0.033	2.276
H	-1.695	0.136	1.863
H	-4.689	8.845	1.045
H	-6.262	8.201	0.640
H	-2.428	1.396	5.876

The parameter files of 5-HIU during MD simulation

Prepin file

```
    0    0    2

This is a remark line
molecule.res
HIU    INT   0
CORRECT     OMIT DU   BEG
  0.0000
   1  DUMM  DU    M    0  -1  -2     0.000      .0        .0      .00000
   2  DUMM  DU    M    1   0  -1     1.449      .0        .0      .00000
   3  DUMM  DU    M    2   1   0     1.523   111.21       .0      .00000
   4  O     o     M    3   2   1     1.540   111.208  -180.000 -0.633500
   5  C     c     M    4   3   2     1.239    89.353   -19.517  0.711500
   6  N     n     M    5   4   3     1.353   123.791  -131.519 -0.638500
   7  H     hn    E    6   5   4     1.033   118.716    -3.901  0.315500
   8  C1    c2    M    6   5   4     1.439   125.970  -175.043  0.425000
   9  O1    o     E    8   6   5     1.230   116.452   166.961 -0.397000
  10  N1    ne    M    8   6   5     1.362   119.857   -16.238 -0.547300
  11  C2    ce    M   10   8   6     1.320   117.366     9.967  0.696300
  12  N2    nf    M   11  10   8     1.328   126.255  -164.768 -0.707100
  13  C3    c     M   12  11  10     1.383   106.915   169.605  0.787900
  14  O2    o     E   13  12  11     1.227   127.046  -178.580 -0.646500
  15  N3    n     M   13  12  11     1.430   110.876    -2.747 -0.518900
  16  H1    hn    E   15  13  12     1.014   114.400   146.359  0.294500
  17  C4    c3    M   15  13  12     1.436   106.678    16.160  0.045900
  18  O3    oh    M   17  15  13     1.424   109.586    98.112 -0.609800
  19  H2    ho    E   18  17  15     1.018   113.518   179.280  0.421000
```

```
 frcmod
remark goes here
MASS

BOND

ANGLE
n -c2-o     74.833     117.460    Calculated with empirical approach
o -c2-ne    78.936     117.755    Calculated with empirical approach
ne-ce-nf    77.960     113.820    same as n2-c2-n2
ne-ce-c3    66.920     122.700    same as c3-ce-n2
ce-nf-c     67.850     118.530    same as c -nf-c2
```

DIHE					
c -c3-ce-ne	1	0.000	0.000	2.000	same as X -c2-c3-X
c -c3-ce-nf	1	0.000	0.000	2.000	same as X -c2-c3-X
ne-ce-nf-c	1	4.150	180.000	2.000	same as X -c2-nf-X
ne-ce-c3-n	1	0.000	0.000	2.000	same as X -c2-c3-X
ne-ce-c3-oh	1	0.000	0.000	2.000	same as X -c2-c3-X
nf-ce-c3-n	1	0.000	0.000	2.000	same as X -c2-c3-X
nf-ce-c3-oh	1	0.000	0.000	2.000	same as X -c2-c3-X
c -nf-ce-c3	1	4.150	180.000	2.000	same as X -c2-nf-X

IMPROPER				
c3-n -c -o	10.5	180.0	2.0	General improper torsional angle (2 general atom types)
c -c2-n -hn	1.1	180.0	2.0	General improper torsional angle (2 general atom types)
n -ne-c2-o	1.1	180.0	2.0	Using default value
c3-ne-ce-nf	1.1	180.0	2.0	Using default value
n -nf-c -o	10.5	180.0	2.0	General improper torsional angle (2 general atom types)

NONBON

The pdb format of all the reaction species computed via DFT.

1a

HETATM	1	C	0	27.476	28.332	31.203	C
HETATM	2	H	0	27.366	27.297	30.953	H
HETATM	3	C	0	27.728	28.511	32.709	C
HETATM	4	H	0	28.583	27.904	33.025	H
HETATM	5	H	0	28.032	29.548	32.908	H
HETATM	6	C	0	26.552	28.180	33.576	C
HETATM	7	N	0	26.722	27.708	34.868	N
HETATM	8	C	0	25.516	27.605	35.403	C
HETATM	9	H	0	25.284	27.305	36.419	H
HETATM	10	N	0	24.570	27.968	34.503	N
HETATM	11	H	0	23.580	28.023	34.689	H
HETATM	12	C	0	25.209	28.339	33.333	C
HETATM	13	H	0	24.666	28.690	32.471	H
HETATM	14	C	0	25.497	24.195	31.031	C
HETATM	15	H	0	26.391	24.721	30.767	H
HETATM	16	C	0	25.702	23.351	32.312	C
HETATM	17	H	0	24.894	22.609	32.379	H
HETATM	18	H	0	26.634	22.774	32.239	H
HETATM	19	C	0	25.700	24.181	33.610	C
HETATM	20	H	0	26.510	24.921	33.598	H
HETATM	21	H	0	24.770	24.761	33.670	H
HETATM	22	C	0	25.807	23.292	34.862	C
HETATM	23	H	0	25.035	22.515	34.818	H
HETATM	24	H	0	26.768	22.768	34.882	H
HETATM	25	N	0	25.641	23.980	36.147	N
HETATM	26	H	0	24.714	24.046	36.547	H
HETATM	27	C	0	26.640	24.373	36.966	C
HETATM	28	N	0	27.913	24.415	36.499	N
HETATM	29	H	0	28.025	24.629	35.519	H
HETATM	30	H	0	28.565	24.926	37.093	H
HETATM	31	N	0	26.390	24.650	38.236	N
HETATM	32	H	0	25.401	24.797	38.553	H
HETATM	33	H	0	27.109	25.198	38.712	H
HETATM	34	C	0	35.188	27.598	31.880	C
HETATM	35	H	0	34.484	27.363	31.109	H

HETATM	36	C	0	34.496	27.526	33.256	C
HETATM	37	H	0	35.120	26.977	33.967	H
HETATM	38	H	0	34.411	28.540	33.666	H
HETATM	39	C	0	33.111	26.941	33.257	C
HETATM	40	N	0	32.301	26.911	32.137	N
HETATM	41	C	0	31.125	26.502	32.568	C
HETATM	42	H	0	30.244	26.364	31.956	H
HETATM	43	N	0	31.136	26.229	33.907	N
HETATM	44	H	0	30.331	26.212	34.524	H
HETATM	45	C	0	32.407	26.519	34.358	C
HETATM	46	H	0	32.697	26.394	35.391	H
HETATM	47	C	0	34.819	35.664	31.905	C
HETATM	48	H	0	35.371	36.567	31.750	H
HETATM	49	C	0	34.091	35.789	33.262	C
HETATM	50	H	0	34.838	35.843	34.063	H
HETATM	51	H	0	33.565	36.755	33.277	H
HETATM	52	C	0	33.104	34.686	33.572	C
HETATM	53	C	0	33.351	33.732	34.565	C
HETATM	54	H	0	34.290	33.769	35.115	H
HETATM	55	C	0	32.424	32.739	34.883	C
HETATM	56	H	0	32.633	32.008	35.658	H
HETATM	57	C	0	31.199	32.684	34.198	C
HETATM	58	O	0	30.246	31.768	34.466	O
HETATM	59	H	0	30.477	31.252	35.280	H
HETATM	60	C	0	30.943	33.624	33.186	C
HETATM	61	H	0	29.992	33.576	32.666	H
HETATM	62	C	0	31.882	34.604	32.888	C
HETATM	63	H	0	31.652	35.337	32.115	H
HETATM	64	C	0	36.058	30.045	35.669	C
HETATM	65	H	0	36.506	30.520	36.517	H
HETATM	66	H	0	34.996	30.171	35.709	H
HETATM	67	C	0	36.405	28.569	35.852	C
HETATM	68	O	0	37.459	28.099	35.407	O
HETATM	69	N	0	35.523	27.900	36.648	N
HETATM	70	H	0	34.592	28.310	36.740	H
HETATM	71	C	0	35.632	26.485	37.071	C
HETATM	72	H	0	35.399	25.829	36.222	H
HETATM	73	C	0	37.026	26.087	37.598	C
HETATM	74	H	0	37.403	26.897	38.242	H
HETATM	75	H	0	36.894	25.213	38.241	H
HETATM	76	O	0	37.939	25.721	36.587	O
HETATM	77	H	0	37.970	26.496	35.990	H
HETATM	78	C	0	34.539	26.257	38.156	C
HETATM	79	O	0	33.363	26.025	37.747	O
HETATM	80	O	0	34.902	26.330	39.364	O
HETATM	81	C	0	31.541	32.283	42.546	C
HETATM	82	H	0	31.753	31.478	43.218	H
HETATM	83	C	0	31.393	31.833	41.074	C
HETATM	84	H	0	30.503	31.204	40.990	H
HETATM	85	H	0	31.186	32.721	40.465	H
HETATM	86	C	0	32.551	31.095	40.477	C
HETATM	87	N	0	32.657	29.715	40.580	N
HETATM	88	C	0	33.755	29.377	39.923	C
HETATM	89	H	0	34.146	28.377	39.789	H
HETATM	90	N	0	34.371	30.473	39.416	N
HETATM	91	H	0	35.157	30.451	38.785	H
HETATM	92	C	0	33.608	31.577	39.745	C

HETATM	93	H	0	33.865	32.572	39.419	H
HETATM	94	C	0	33.624	29.523	45.335	C
HETATM	95	H	0	33.362	30.228	44.575	H
HETATM	96	C	0	33.301	28.093	44.848	C
HETATM	97	H	0	33.745	27.365	45.540	H
HETATM	98	H	0	32.214	27.926	44.902	H
HETATM	99	C	0	33.774	27.811	43.409	C
HETATM	100	H	0	33.209	28.439	42.711	H
HETATM	101	H	0	34.822	28.114	43.297	H
HETATM	102	C	0	33.629	26.319	42.999	C
HETATM	103	H	0	34.574	25.791	43.153	H
HETATM	104	H	0	32.903	25.819	43.655	H
HETATM	105	N	0	33.232	26.068	41.615	N
HETATM	106	H	0	33.942	26.018	40.860	H
HETATM	107	C	0	31.970	26.215	41.187	C
HETATM	108	N	0	30.960	26.557	42.041	N
HETATM	109	H	0	31.271	26.959	42.913	H
HETATM	110	H	0	30.293	27.177	41.549	H
HETATM	111	N	0	31.655	25.970	39.921	N
HETATM	112	H	0	32.381	25.891	39.176	H
HETATM	113	H	0	30.713	26.200	39.616	H
HETATM	114	C	0	24.033	31.460	42.887	C
HETATM	115	H	0	24.803	31.665	43.601	H
HETATM	116	C	0	24.547	30.380	41.888	C
HETATM	117	H	0	23.959	29.461	41.994	H
HETATM	118	H	0	24.401	30.706	40.857	H
HETATM	119	C	0	25.997	30.076	42.061	C
HETATM	120	N	0	26.480	29.377	43.158	N
HETATM	121	C	0	27.835	29.311	43.051	C
HETATM	122	H	0	28.456	28.799	43.773	H
HETATM	123	N	0	28.253	29.930	41.967	N
HETATM	124	C	0	27.118	30.411	41.344	C
HETATM	125	H	0	27.177	30.969	40.419	H
HETATM	126	C	0	24.062	37.650	37.529	C
HETATM	127	H	0	23.760	38.612	37.172	H
HETATM	128	C	0	24.742	36.852	36.388	C
HETATM	129	H	0	23.983	36.458	35.701	H
HETATM	130	H	0	25.349	37.551	35.796	H
HETATM	131	C	0	25.634	35.722	36.870	C
HETATM	132	C	0	25.392	34.381	36.549	C
HETATM	133	H	0	24.519	34.129	35.950	H
HETATM	134	C	0	26.240	33.354	36.971	C
HETATM	135	H	0	26.029	32.317	36.729	H
HETATM	136	C	0	27.377	33.656	37.736	C
HETATM	137	O	0	28.247	32.722	38.185	O
HETATM	138	H	0	28.196	31.882	37.660	H
HETATM	139	C	0	27.627	34.995	38.075	C
HETATM	140	H	0	28.515	35.222	38.658	H
HETATM	141	C	0	26.771	36.001	37.645	C
HETATM	142	H	0	27.004	37.033	37.901	H
HETATM	143	C	0	22.425	31.109	38.386	C
HETATM	144	H	0	21.606	31.057	37.699	H
HETATM	145	H	0	23.347	31.024	37.849	H
HETATM	146	C	0	22.200	29.905	39.303	C
HETATM	147	O	0	21.287	29.917	40.144	O
HETATM	148	N	0	22.967	28.824	39.027	N
HETATM	149	H	0	23.822	28.934	38.482	H

HETATM	150	C	0	22.867	27.491	39.639	C
HETATM	151	H	0	23.157	27.537	40.701	H
HETATM	152	C	0	21.456	26.875	39.611	C
HETATM	153	H	0	21.015	27.060	38.614	H
HETATM	154	H	0	21.579	25.796	39.724	H
HETATM	155	O	0	20.622	27.320	40.658	O
HETATM	156	H	0	20.675	28.298	40.636	H
HETATM	157	C	0	23.935	26.590	38.941	C
HETATM	158	O	0	23.814	25.338	39.016	O
HETATM	159	O	0	24.878	27.205	38.369	O
HETATM	160	O	0	28.755	26.304	38.624	O
HETATM	161	C	0	28.630	27.438	38.123	C
HETATM	162	N	0	27.500	28.174	38.225	N
HETATM	163	H	0	26.653	27.757	38.627	H
HETATM	164	C	0	27.267	29.482	37.668	C
HETATM	165	O	0	26.139	29.950	37.802	O
HETATM	166	N	0	28.301	30.154	37.089	N
HETATM	167	C	0	29.455	29.522	36.938	C
HETATM	168	N	0	30.612	30.084	36.643	N
HETATM	169	C	0	31.596	29.110	36.838	C
HETATM	170	O	0	32.803	29.281	36.694	O
HETATM	171	N	0	31.009	27.922	37.288	N
HETATM	172	H	0	31.564	27.073	37.292	H
HETATM	173	C	0	29.605	27.996	37.088	C
HETATM	174	O	0	29.223	27.289	35.890	O
HETATM	175	H	0	28.294	27.526	35.592	H
HETATM	176	H	0	30.601	32.725	42.803	H
HETATM	177	H	0	32.313	33.020	42.618	H
HETATM	178	H	0	34.674	29.586	45.533	H
HETATM	179	H	0	33.079	29.743	46.229	H
HETATM	180	H	0	23.796	32.355	42.350	H
HETATM	181	H	0	23.160	31.108	43.396	H
HETATM	182	H	0	34.106	35.542	31.116	H
HETATM	183	H	0	35.491	34.831	31.910	H
HETATM	184	H	0	35.560	28.591	31.733	H
HETATM	185	H	0	36.001	26.904	31.844	H
HETATM	186	H	0	25.202	23.563	30.220	H
HETATM	187	H	0	24.717	24.898	31.238	H
HETATM	188	H	0	28.320	28.736	30.685	H
HETATM	189	H	0	26.593	28.864	30.916	H
HETATM	190	H	0	22.398	32.045	38.903	H
HETATM	191	H	0	36.436	30.487	34.771	H
HETATM	192	O	0	30.006	28.520	40.326	O
HETATM	193	H	0	30.916	28.892	40.345	H
HETATM	194	H	0	29.458	29.141	40.856	H
HETATM	195	H	0	24.782	37.770	38.312	H
HETATM	196	H	0	23.209	37.126	37.906	H
HETATM	197	H	0	25.917	28.965	43.887	H

TS1a

HETATM	1	C	0	27.476	28.332	31.203	C
HETATM	2	H	0	27.366	27.297	30.953	H
HETATM	3	C	0	27.741	28.501	32.708	C
HETATM	4	H	0	28.601	27.894	33.010	H
HETATM	5	H	0	28.038	29.538	32.914	H
HETATM	6	C	0	26.577	28.145	33.579	C
HETATM	7	N	0	26.758	27.548	34.816	N

HETATM	8	C	0	25.563	27.463	35.377	C
HETATM	9	H	0	25.341	27.113	36.378	H
HETATM	10	N	0	24.612	27.948	34.542	N
HETATM	11	H	0	23.636	28.055	34.773	H
HETATM	12	C	0	25.239	28.393	33.392	C
HETATM	13	H	0	24.693	28.851	32.583	H
HETATM	14	C	0	25.497	24.195	31.031	C
HETATM	15	H	0	26.391	24.721	30.767	H
HETATM	16	C	0	25.719	23.352	32.313	C
HETATM	17	H	0	24.918	22.603	32.390	H
HETATM	18	H	0	26.654	22.782	32.227	H
HETATM	19	C	0	25.730	24.180	33.615	C
HETATM	20	H	0	26.495	24.964	33.569	H
HETATM	21	H	0	24.773	24.707	33.717	H
HETATM	22	C	0	25.946	23.301	34.864	C
HETATM	23	H	0	25.237	22.465	34.843	H
HETATM	24	H	0	26.946	22.853	34.850	H
HETATM	25	N	0	25.771	23.969	36.160	N
HETATM	26	H	0	24.848	23.988	36.571	H
HETATM	27	C	0	26.759	24.449	36.950	C
HETATM	28	N	0	28.022	24.553	36.479	N
HETATM	29	H	0	28.109	24.728	35.489	H
HETATM	30	H	0	28.608	25.186	37.044	H
HETATM	31	N	0	26.519	24.765	38.215	N
HETATM	32	H	0	25.542	24.873	38.558	H
HETATM	33	H	0	27.243	25.360	38.641	H
HETATM	34	C	0	35.188	27.598	31.880	C
HETATM	35	H	0	34.484	27.363	31.109	H
HETATM	36	C	0	34.489	27.530	33.253	C
HETATM	37	H	0	35.140	27.053	33.992	H
HETATM	38	H	0	34.324	28.550	33.622	H
HETATM	39	C	0	33.148	26.854	33.264	C
HETATM	40	N	0	32.296	26.864	32.173	N
HETATM	41	C	0	31.156	26.380	32.618	C
HETATM	42	H	0	30.255	26.252	32.031	H
HETATM	43	N	0	31.227	26.014	33.933	N
HETATM	44	H	0	30.441	25.963	34.571	H
HETATM	45	C	0	32.505	26.326	34.356	C
HETATM	46	H	0	32.841	26.150	35.368	H
HETATM	47	C	0	34.819	35.664	31.905	C
HETATM	48	H	0	35.371	36.567	31.750	H
HETATM	49	C	0	34.075	35.790	33.253	C
HETATM	50	H	0	34.808	35.797	34.070	H
HETATM	51	H	0	33.582	36.772	33.279	H
HETATM	52	C	0	33.047	34.712	33.500	C
HETATM	53	C	0	33.295	33.638	34.361	C
HETATM	54	H	0	34.254	33.578	34.873	H
HETATM	55	C	0	32.348	32.642	34.586	C
HETATM	56	H	0	32.561	31.815	35.255	H
HETATM	57	C	0	31.099	32.706	33.944	C
HETATM	58	O	0	30.133	31.786	34.133	O
HETATM	59	H	0	30.383	31.189	34.888	H
HETATM	60	C	0	30.839	33.772	33.068	C
HETATM	61	H	0	29.869	33.819	32.583	H
HETATM	62	C	0	31.800	34.754	32.857	C
HETATM	63	H	0	31.574	35.582	32.187	H
HETATM	64	C	0	36.058	30.045	35.669	C

HETATM	65	H	0	36.506	30.520	36.517	H
HETATM	66	H	0	34.996	30.171	35.709	H
HETATM	67	C	0	36.456	28.592	35.887	C
HETATM	68	O	0	37.551	28.156	35.510	O
HETATM	69	N	0	35.576	27.909	36.673	N
HETATM	70	H	0	34.620	28.267	36.675	H
HETATM	71	C	0	35.773	26.549	37.215	C
HETATM	72	H	0	35.648	25.804	36.418	H
HETATM	73	C	0	37.157	26.319	37.854	C
HETATM	74	H	0	37.428	27.212	38.440	H
HETATM	75	H	0	37.057	25.495	38.567	H
HETATM	76	O	0	38.157	25.948	36.932	O
HETATM	77	H	0	38.149	26.662	36.261	H
HETATM	78	C	0	34.629	26.334	38.242	C
HETATM	79	O	0	33.501	26.012	37.757	O
HETATM	80	O	0	34.876	26.532	39.465	O
HETATM	81	C	0	31.541	32.283	42.546	C
HETATM	82	H	0	31.753	31.478	43.218	H
HETATM	83	C	0	31.389	31.787	41.089	C
HETATM	84	H	0	30.398	31.340	40.958	H
HETATM	85	H	0	31.387	32.656	40.419	H
HETATM	86	C	0	32.378	30.770	40.601	C
HETATM	87	N	0	31.944	29.716	39.813	N
HETATM	88	C	0	33.019	29.021	39.496	C
HETATM	89	H	0	33.032	28.138	38.884	H
HETATM	90	N	0	34.131	29.561	40.042	N
HETATM	91	H	0	35.047	29.145	39.955	H
HETATM	92	C	0	33.744	30.683	40.754	C
HETATM	93	H	0	34.455	31.309	41.270	H
HETATM	94	C	0	33.624	29.523	45.335	C
HETATM	95	H	0	33.362	30.228	44.575	H
HETATM	96	C	0	33.232	28.105	44.826	C
HETATM	97	H	0	33.856	27.347	45.317	H
HETATM	98	H	0	32.202	27.886	45.142	H
HETATM	99	C	0	33.305	27.932	43.295	C
HETATM	100	H	0	32.613	28.634	42.813	H
HETATM	101	H	0	34.302	28.201	42.926	H
HETATM	102	C	0	32.970	26.494	42.841	C
HETATM	103	H	0	33.783	25.822	43.141	H
HETATM	104	H	0	32.075	26.135	43.360	H
HETATM	105	N	0	32.763	26.320	41.404	N
HETATM	106	H	0	33.582	26.310	40.764	H
HETATM	107	C	0	31.568	26.160	40.812	C
HETATM	108	N	0	30.401	26.331	41.515	N
HETATM	109	H	0	30.431	27.053	42.220	H
HETATM	110	H	0	29.600	26.427	40.889	H
HETATM	111	N	0	31.469	25.769	39.553	N
HETATM	112	H	0	32.315	25.729	38.941	H
HETATM	113	H	0	30.543	25.901	39.104	H
HETATM	114	C	0	24.033	31.460	42.887	C
HETATM	115	H	0	24.803	31.665	43.601	H
HETATM	116	C	0	24.457	30.303	41.964	C
HETATM	117	H	0	24.139	29.357	42.421	H
HETATM	118	H	0	23.929	30.355	41.014	H
HETATM	119	C	0	25.913	30.197	41.673	C
HETATM	120	N	0	26.894	30.191	42.658	N
HETATM	121	C	0	28.085	29.915	42.085	C

HETATM	122	H	0	29.024	29.853	42.611	H
HETATM	123	N	0	27.907	29.750	40.785	N
HETATM	124	C	0	26.569	29.926	40.506	C
HETATM	125	H	0	26.177	29.847	39.499	H
HETATM	126	C	0	24.062	37.650	37.529	C
HETATM	127	H	0	23.760	38.612	37.172	H
HETATM	128	C	0	24.723	36.863	36.368	C
HETATM	129	H	0	23.947	36.492	35.687	H
HETATM	130	H	0	25.321	37.572	35.778	H
HETATM	131	C	0	25.621	35.714	36.789	C
HETATM	132	C	0	25.375	34.389	36.406	C
HETATM	133	H	0	24.480	34.162	35.829	H
HETATM	134	C	0	26.250	33.348	36.728	C
HETATM	135	H	0	26.038	32.323	36.436	H
HETATM	136	C	0	27.423	33.618	37.450	C
HETATM	137	O	0	28.331	32.679	37.788	O
HETATM	138	H	0	28.238	31.843	37.257	H
HETATM	139	C	0	27.671	34.939	37.862	C
HETATM	140	H	0	28.584	35.143	38.413	H
HETATM	141	C	0	26.787	35.959	37.532	C
HETATM	142	H	0	27.024	36.978	37.836	H
HETATM	143	C	0	22.425	31.109	38.386	C
HETATM	144	H	0	21.606	31.057	37.699	H
HETATM	145	H	0	23.347	31.024	37.849	H
HETATM	146	C	0	22.203	29.913	39.311	C
HETATM	147	O	0	21.329	29.946	40.194	O
HETATM	148	N	0	22.950	28.825	39.023	N
HETATM	149	H	0	23.754	28.897	38.397	H
HETATM	150	C	0	22.888	27.505	39.667	C
HETATM	151	H	0	23.187	27.590	40.724	H
HETATM	152	C	0	21.494	26.858	39.671	C
HETATM	153	H	0	21.042	26.994	38.672	H
HETATM	154	H	0	21.641	25.787	39.826	H
HETATM	155	O	0	20.654	27.328	40.706	O
HETATM	156	H	0	20.707	28.304	40.668	H
HETATM	157	C	0	23.974	26.620	38.972	C
HETATM	158	O	0	23.912	25.372	39.114	O
HETATM	159	O	0	24.859	27.260	38.337	O
HETATM	160	O	0	28.874	26.385	38.619	O
HETATM	161	C	0	28.894	27.653	38.233	C
HETATM	162	N	0	27.572	28.214	38.000	N
HETATM	163	H	0	26.751	27.718	38.344	H
HETATM	164	C	0	27.310	29.419	37.357	C
HETATM	165	O	0	26.164	29.898	37.399	O
HETATM	166	N	0	28.342	30.100	36.739	N
HETATM	167	C	0	29.499	29.479	36.606	C
HETATM	168	N	0	30.631	30.033	36.188	N
HETATM	169	C	0	31.650	29.123	36.437	C
HETATM	170	O	0	32.850	29.293	36.225	O
HETATM	171	N	0	31.133	27.982	37.072	N
HETATM	172	H	0	31.687	27.134	37.042	H
HETATM	173	C	0	29.707	27.989	36.957	C
HETATM	174	O	0	29.281	27.129	35.904	O
HETATM	175	H	0	28.353	27.349	35.605	H
HETATM	176	H	0	30.601	32.725	42.803	H
HETATM	177	H	0	32.313	33.020	42.618	H
HETATM	178	H	0	34.674	29.586	45.533	H

HETATM	179	H	0	33.079	29.743	46.229	H
HETATM	180	H	0	23.796	32.355	42.350	H
HETATM	181	H	0	23.160	31.108	43.396	H
HETATM	182	H	0	34.106	35.542	31.116	H
HETATM	183	H	0	35.491	34.831	31.910	H
HETATM	184	H	0	35.560	28.591	31.733	H
HETATM	185	H	0	36.001	26.904	31.844	H
HETATM	186	H	0	25.202	23.563	30.220	H
HETATM	187	H	0	24.717	24.898	31.238	H
HETATM	188	H	0	28.320	28.736	30.685	H
HETATM	189	H	0	26.593	28.864	30.916	H
HETATM	190	H	0	22.398	32.045	38.903	H
HETATM	191	H	0	36.436	30.487	34.771	H
HETATM	192	O	0	29.500	28.506	39.363	O
HETATM	193	H	0	30.402	28.917	39.244	H
HETATM	194	H	0	28.693	29.243	40.039	H
HETATM	195	H	0	24.782	37.770	38.312	H
HETATM	196	H	0	23.209	37.126	37.906	H
HETATM	197	H	0	26.746	30.369	43.639	H

INT1a

HETATM	1	C	0	27.476	28.332	31.203	C
HETATM	2	H	0	27.366	27.297	30.953	H
HETATM	3	C	0	27.743	28.499	32.708	C
HETATM	4	H	0	28.604	27.892	33.007	H
HETATM	5	H	0	28.040	29.536	32.915	H
HETATM	6	C	0	26.581	28.141	33.582	C
HETATM	7	N	0	26.766	27.529	34.810	N
HETATM	8	C	0	25.575	27.448	35.379	C
HETATM	9	H	0	25.358	27.093	36.378	H
HETATM	10	N	0	24.621	27.949	34.556	N
HETATM	11	H	0	23.649	28.063	34.795	H
HETATM	12	C	0	25.244	28.402	33.406	C
HETATM	13	H	0	24.697	28.873	32.606	H
HETATM	14	C	0	25.497	24.195	31.031	C
HETATM	15	H	0	26.391	24.721	30.767	H
HETATM	16	C	0	25.719	23.350	32.312	C
HETATM	17	H	0	24.917	22.602	32.388	H
HETATM	18	H	0	26.653	22.779	32.223	H
HETATM	19	C	0	25.734	24.176	33.614	C
HETATM	20	H	0	26.501	24.959	33.568	H
HETATM	21	H	0	24.778	24.705	33.719	H
HETATM	22	C	0	25.952	23.296	34.862	C
HETATM	23	H	0	25.241	22.461	34.842	H
HETATM	24	H	0	26.950	22.845	34.844	H
HETATM	25	N	0	25.782	23.962	36.158	N
HETATM	26	H	0	24.860	23.984	36.572	H
HETATM	27	C	0	26.774	24.439	36.946	C
HETATM	28	N	0	28.037	24.539	36.475	N
HETATM	29	H	0	28.124	24.710	35.484	H
HETATM	30	H	0	28.625	25.172	37.041	H
HETATM	31	N	0	26.537	24.759	38.211	N
HETATM	32	H	0	25.561	24.873	38.555	H
HETATM	33	H	0	27.267	25.349	38.633	H
HETATM	34	C	0	35.188	27.598	31.880	C
HETATM	35	H	0	34.484	27.363	31.109	H
HETATM	36	C	0	34.485	27.529	33.250	C

HETATM	37	H	0	35.140	27.070	33.996	H
HETATM	38	H	0	34.298	28.548	33.611	H
HETATM	39	C	0	33.157	26.827	33.258	C
HETATM	40	N	0	32.301	26.840	32.169	N
HETATM	41	C	0	31.171	26.330	32.610	C
HETATM	42	H	0	30.270	26.197	32.025	H
HETATM	43	N	0	31.254	25.941	33.918	N
HETATM	44	H	0	30.469	25.877	34.556	H
HETATM	45	C	0	32.528	26.270	34.342	C
HETATM	46	H	0	32.869	26.085	35.351	H
HETATM	47	C	0	34.819	35.664	31.905	C
HETATM	48	H	0	35.371	36.567	31.750	H
HETATM	49	C	0	34.075	35.792	33.253	C
HETATM	50	H	0	34.808	35.798	34.070	H
HETATM	51	H	0	33.584	36.774	33.279	H
HETATM	52	C	0	33.046	34.715	33.498	C
HETATM	53	C	0	33.299	33.631	34.345	C
HETATM	54	H	0	34.262	33.564	34.849	H
HETATM	55	C	0	32.353	32.633	34.565	C
HETATM	56	H	0	32.570	31.798	35.223	H
HETATM	57	C	0	31.100	32.706	33.933	C
HETATM	58	O	0	30.135	31.784	34.118	O
HETATM	59	H	0	30.394	31.176	34.862	H
HETATM	60	C	0	30.834	33.783	33.072	C
HETATM	61	H	0	29.860	33.836	32.595	H
HETATM	62	C	0	31.795	34.766	32.865	C
HETATM	63	H	0	31.564	35.602	32.207	H
HETATM	64	C	0	36.058	30.045	35.669	C
HETATM	65	H	0	36.506	30.520	36.517	H
HETATM	66	H	0	34.996	30.171	35.709	H
HETATM	67	C	0	36.465	28.594	35.886	C
HETATM	68	O	0	37.566	28.169	35.512	O
HETATM	69	N	0	35.590	27.903	36.667	N
HETATM	70	H	0	34.629	28.251	36.662	H
HETATM	71	C	0	35.797	26.546	37.212	C
HETATM	72	H	0	35.682	25.798	36.417	H
HETATM	73	C	0	37.183	26.330	37.855	C
HETATM	74	H	0	37.443	27.227	38.441	H
HETATM	75	H	0	37.088	25.507	38.569	H
HETATM	76	O	0	38.188	25.967	36.936	O
HETATM	77	H	0	38.176	26.681	36.264	H
HETATM	78	C	0	34.652	26.325	38.236	C
HETATM	79	O	0	33.527	26.002	37.747	O
HETATM	80	O	0	34.896	26.519	39.461	O
HETATM	81	C	0	31.541	32.283	42.546	C
HETATM	82	H	0	31.753	31.478	43.218	H
HETATM	83	C	0	31.401	31.788	41.087	C
HETATM	84	H	0	30.416	31.333	40.947	H
HETATM	85	H	0	31.397	32.659	40.419	H
HETATM	86	C	0	32.403	30.781	40.602	C
HETATM	87	N	0	31.983	29.727	39.805	N
HETATM	88	C	0	33.066	29.044	39.491	C
HETATM	89	H	0	33.090	28.162	38.876	H
HETATM	90	N	0	34.170	29.591	40.048	N
HETATM	91	H	0	35.093	29.188	39.963	H
HETATM	92	C	0	33.769	30.705	40.763	C
HETATM	93	H	0	34.471	31.335	41.287	H

HETATM	94	C	0	33.624	29.523	45.335	C
HETATM	95	H	0	33.362	30.228	44.575	H
HETATM	96	C	0	33.233	28.106	44.823	C
HETATM	97	H	0	33.856	27.347	45.313	H
HETATM	98	H	0	32.203	27.885	45.135	H
HETATM	99	C	0	33.311	27.936	43.291	C
HETATM	100	H	0	32.619	28.637	42.808	H
HETATM	101	H	0	34.309	28.208	42.925	H
HETATM	102	C	0	32.981	26.498	42.832	C
HETATM	103	H	0	33.794	25.827	43.135	H
HETATM	104	H	0	32.085	26.136	43.347	H
HETATM	105	N	0	32.780	26.325	41.394	N
HETATM	106	H	0	33.602	26.312	40.757	H
HETATM	107	C	0	31.588	26.158	40.797	C
HETATM	108	N	0	30.417	26.325	41.497	N
HETATM	109	H	0	30.444	27.051	42.199	H
HETATM	110	H	0	29.621	26.419	40.867	H
HETATM	111	N	0	31.494	25.765	39.540	N
HETATM	112	H	0	32.342	25.726	38.929	H
HETATM	113	H	0	30.567	25.888	39.087	H
HETATM	114	C	0	24.099	31.418	42.825	C
HETATM	115	H	0	24.869	31.623	43.538	H
HETATM	116	C	0	24.519	30.257	41.909	C
HETATM	117	H	0	24.210	29.312	42.375	H
HETATM	118	H	0	23.988	30.297	40.960	H
HETATM	119	C	0	25.972	30.164	41.617	C
HETATM	120	N	0	26.955	30.163	42.601	N
HETATM	121	C	0	28.148	29.906	42.030	C
HETATM	122	H	0	29.090	29.847	42.550	H
HETATM	123	N	0	27.964	29.752	40.728	N
HETATM	124	C	0	26.626	29.911	40.446	C
HETATM	125	H	0	26.235	29.834	39.438	H
HETATM	126	C	0	24.062	37.650	37.529	C
HETATM	127	H	0	23.760	38.612	37.172	H
HETATM	128	C	0	24.723	36.860	36.370	C
HETATM	129	H	0	23.948	36.495	35.685	H
HETATM	130	H	0	25.330	37.565	35.784	H
HETATM	131	C	0	25.611	35.702	36.791	C
HETATM	132	C	0	25.369	34.383	36.386	C
HETATM	133	H	0	24.481	34.166	35.795	H
HETATM	134	C	0	26.238	33.335	36.703	C
HETATM	135	H	0	26.028	32.315	36.393	H
HETATM	136	C	0	27.403	33.592	37.443	C
HETATM	137	O	0	28.306	32.647	37.778	O
HETATM	138	H	0	28.230	31.822	37.229	H
HETATM	139	C	0	27.647	34.906	37.879	C
HETATM	140	H	0	28.553	35.101	38.444	H
HETATM	141	C	0	26.768	35.933	37.553	C
HETATM	142	H	0	27.004	36.946	37.875	H
HETATM	143	C	0	22.425	31.109	38.386	C
HETATM	144	H	0	21.606	31.057	37.699	H
HETATM	145	H	0	23.347	31.024	37.849	H
HETATM	146	C	0	22.205	29.911	39.309	C
HETATM	147	O	0	21.334	29.940	40.194	O
HETATM	148	N	0	22.951	28.823	39.014	N
HETATM	149	H	0	23.756	28.898	38.390	H
HETATM	150	C	0	22.891	27.503	39.658	C

HETATM	151	H	0	23.180	27.589	40.717	H
HETATM	152	C	0	21.500	26.849	39.649	C
HETATM	153	H	0	21.056	26.984	38.646	H
HETATM	154	H	0	21.651	25.778	39.804	H
HETATM	155	O	0	20.650	27.314	40.678	O
HETATM	156	H	0	20.703	28.290	40.647	H
HETATM	157	C	0	23.990	26.624	38.972	C
HETATM	158	O	0	23.927	25.375	39.105	O
HETATM	159	O	0	24.881	27.270	38.355	O
HETATM	160	O	0	28.911	26.352	38.574	O
HETATM	161	C	0	28.941	27.629	38.199	C
HETATM	162	N	0	27.612	28.191	37.970	N
HETATM	163	H	0	26.794	27.695	38.319	H
HETATM	164	C	0	27.346	29.392	37.326	C
HETATM	165	O	0	26.201	29.875	37.377	O
HETATM	166	N	0	28.373	30.073	36.700	N
HETATM	167	C	0	29.529	29.452	36.564	C
HETATM	168	N	0	30.660	30.009	36.144	N
HETATM	169	C	0	31.681	29.102	36.393	C
HETATM	170	O	0	32.881	29.277	36.183	O
HETATM	171	N	0	31.167	27.958	37.024	N
HETATM	172	H	0	31.727	27.114	37.003	H
HETATM	173	C	0	29.741	27.962	36.913	C
HETATM	174	O	0	29.311	27.105	35.860	O
HETATM	175	H	0	28.378	27.320	35.577	H
HETATM	176	H	0	30.601	32.725	42.803	H
HETATM	177	H	0	32.313	33.020	42.618	H
HETATM	178	H	0	34.674	29.586	45.533	H
HETATM	179	H	0	33.079	29.743	46.229	H
HETATM	180	H	0	23.862	32.312	42.288	H
HETATM	181	H	0	23.226	31.066	43.333	H
HETATM	182	H	0	34.106	35.542	31.116	H
HETATM	183	H	0	35.491	34.831	31.910	H
HETATM	184	H	0	35.560	28.591	31.733	H
HETATM	185	H	0	36.001	26.904	31.844	H
HETATM	186	H	0	25.202	23.563	30.220	H
HETATM	187	H	0	24.717	24.898	31.238	H
HETATM	188	H	0	28.320	28.736	30.685	H
HETATM	189	H	0	26.593	28.864	30.916	H
HETATM	190	H	0	22.398	32.045	38.903	H
HETATM	191	H	0	36.436	30.487	34.771	H
HETATM	192	O	0	29.549	28.458	39.310	O
HETATM	193	H	0	30.447	28.855	39.165	H
HETATM	194	H	0	28.714	29.279	40.017	H
HETATM	195	H	0	24.782	37.770	38.312	H
HETATM	196	H	0	23.209	37.126	37.906	H
HETATM	197	H	0	26.805	30.334	43.584	H

TS2a

HETATM	1	C	0	27.476	28.332	31.203	C
HETATM	2	H	0	27.366	27.297	30.953	H
HETATM	3	C	0	27.727	28.489	32.710	C
HETATM	4	H	0	28.577	27.875	33.023	H
HETATM	5	H	0	28.014	29.525	32.934	H
HETATM	6	C	0	26.538	28.111	33.536	C
HETATM	7	N	0	26.670	27.404	34.720	N
HETATM	8	C	0	25.455	27.295	35.228	C

HETATM	9	H	0	25.197	26.883	36.194	H
HETATM	10	N	0	24.540	27.875	34.415	N
HETATM	11	H	0	23.559	27.979	34.622	H
HETATM	12	C	0	25.213	28.406	33.330	C
HETATM	13	H	0	24.703	28.940	32.545	H
HETATM	14	C	0	25.497	24.195	31.031	C
HETATM	15	H	0	26.391	24.721	30.767	H
HETATM	16	C	0	25.713	23.327	32.293	C
HETATM	17	H	0	24.901	22.589	32.358	H
HETATM	18	H	0	26.639	22.745	32.189	H
HETATM	19	C	0	25.745	24.127	33.607	C
HETATM	20	H	0	26.537	24.884	33.582	H
HETATM	21	H	0	24.807	24.686	33.715	H
HETATM	22	C	0	25.920	23.217	34.838	C
HETATM	23	H	0	25.160	22.427	34.816	H
HETATM	24	H	0	26.890	22.707	34.802	H
HETATM	25	N	0	25.806	23.877	36.139	N
HETATM	26	H	0	24.896	23.948	36.574	H
HETATM	27	C	0	26.832	24.375	36.865	C
HETATM	28	N	0	28.082	24.428	36.362	N
HETATM	29	H	0	28.165	24.535	35.362	H
HETATM	30	H	0	28.694	25.079	36.894	H
HETATM	31	N	0	26.626	24.765	38.120	N
HETATM	32	H	0	25.658	24.895	38.469	H
HETATM	33	H	0	27.385	25.343	38.506	H
HETATM	34	C	0	35.188	27.598	31.880	C
HETATM	35	H	0	34.484	27.363	31.109	H
HETATM	36	C	0	34.439	27.473	33.216	C
HETATM	37	H	0	35.125	27.597	34.056	H
HETATM	38	H	0	33.709	28.288	33.303	H
HETATM	39	C	0	33.728	26.154	33.368	C
HETATM	40	N	0	33.113	25.524	32.298	N
HETATM	41	C	0	32.568	24.433	32.789	C
HETATM	42	H	0	32.009	23.695	32.230	H
HETATM	43	N	0	32.796	24.317	34.129	N
HETATM	44	H	0	32.485	23.575	34.736	H
HETATM	45	C	0	33.541	25.415	34.515	C
HETATM	46	H	0	33.835	25.561	35.546	H
HETATM	47	C	0	34.819	35.664	31.905	C
HETATM	48	H	0	35.371	36.567	31.750	H
HETATM	49	C	0	34.072	35.796	33.249	C
HETATM	50	H	0	34.799	35.797	34.071	H
HETATM	51	H	0	33.581	36.778	33.275	H
HETATM	52	C	0	33.042	34.717	33.473	C
HETATM	53	C	0	33.329	33.575	34.228	C
HETATM	54	H	0	34.308	33.477	34.695	H
HETATM	55	C	0	32.395	32.558	34.400	C
HETATM	56	H	0	32.635	31.678	34.989	H
HETATM	57	C	0	31.125	32.669	33.811	C
HETATM	58	O	0	30.176	31.719	33.950	O
HETATM	59	H	0	30.479	31.066	34.632	H
HETATM	60	C	0	30.822	33.806	33.047	C
HETATM	61	H	0	29.833	33.888	32.608	H
HETATM	62	C	0	31.772	34.809	32.887	C
HETATM	63	H	0	31.519	35.691	32.301	H
HETATM	64	C	0	36.058	30.045	35.669	C
HETATM	65	H	0	36.506	30.520	36.517	H

HETATM	66	H	0	34.996	30.171	35.709	H
HETATM	67	C	0	36.537	28.623	35.920	C
HETATM	68	O	0	37.675	28.264	35.589	O
HETATM	69	N	0	35.697	27.900	36.710	N
HETATM	70	H	0	34.715	28.171	36.656	H
HETATM	71	C	0	36.014	26.615	37.361	C
HETATM	72	H	0	36.018	25.804	36.621	H
HETATM	73	C	0	37.384	26.592	38.070	C
HETATM	74	H	0	37.531	27.555	38.588	H
HETATM	75	H	0	37.340	25.823	38.847	H
HETATM	76	O	0	38.458	26.260	37.220	O
HETATM	77	H	0	38.399	26.906	36.485	H
HETATM	78	C	0	34.848	26.350	38.350	C
HETATM	79	O	0	33.806	25.837	37.847	O
HETATM	80	O	0	34.997	26.694	39.560	O
HETATM	81	C	0	31.541	32.283	42.546	C
HETATM	82	H	0	31.753	31.478	43.218	H
HETATM	83	C	0	31.405	31.819	41.075	C
HETATM	84	H	0	30.405	31.407	40.910	H
HETATM	85	H	0	31.440	32.707	40.430	H
HETATM	86	C	0	32.370	30.786	40.563	C
HETATM	87	N	0	31.932	29.860	39.631	N
HETATM	88	C	0	32.972	29.099	39.353	C
HETATM	89	H	0	32.968	28.260	38.677	H
HETATM	90	N	0	34.069	29.485	40.044	N
HETATM	91	H	0	34.930	28.949	40.032	H
HETATM	92	C	0	33.704	30.563	40.830	C
HETATM	93	H	0	34.406	31.066	41.476	H
HETATM	94	C	0	33.624	29.523	45.335	C
HETATM	95	H	0	33.362	30.228	44.575	H
HETATM	96	C	0	33.228	28.107	44.824	C
HETATM	97	H	0	33.835	27.344	45.328	H
HETATM	98	H	0	32.190	27.901	45.122	H
HETATM	99	C	0	33.327	27.935	43.295	C
HETATM	100	H	0	32.666	28.658	42.803	H
HETATM	101	H	0	34.340	28.177	42.950	H
HETATM	102	C	0	32.962	26.512	42.824	C
HETATM	103	H	0	33.735	25.810	43.163	H
HETATM	104	H	0	32.031	26.186	43.299	H
HETATM	105	N	0	32.819	26.347	41.378	N
HETATM	106	H	0	33.665	26.368	40.776	H
HETATM	107	C	0	31.654	26.159	40.733	C
HETATM	108	N	0	30.456	26.340	41.378	N
HETATM	109	H	0	30.462	27.086	42.060	H
HETATM	110	H	0	29.697	26.453	40.708	H
HETATM	111	N	0	31.624	25.729	39.484	N
HETATM	112	H	0	32.505	25.663	38.927	H
HETATM	113	H	0	30.729	25.831	38.977	H
HETATM	114	C	0	24.099	31.418	42.825	C
HETATM	115	H	0	24.869	31.623	43.538	H
HETATM	116	C	0	24.431	30.169	42.012	C
HETATM	117	H	0	24.345	29.313	42.698	H
HETATM	118	H	0	23.655	30.011	41.264	H
HETATM	119	C	0	25.746	30.072	41.346	C
HETATM	120	N	0	26.980	30.201	41.973	N
HETATM	121	C	0	27.949	29.814	41.105	C
HETATM	122	H	0	29.007	29.796	41.307	H

HETATM	123	N	0	27.380	29.461	39.973	N
HETATM	124	C	0	26.027	29.616	40.095	C
HETATM	125	H	0	25.373	29.331	39.288	H
HETATM	126	C	0	24.062	37.650	37.529	C
HETATM	127	H	0	23.760	38.612	37.172	H
HETATM	128	C	0	24.708	36.874	36.350	C
HETATM	129	H	0	23.920	36.529	35.670	H
HETATM	130	H	0	25.305	37.590	35.768	H
HETATM	131	C	0	25.605	35.700	36.709	C
HETATM	132	C	0	25.386	34.412	36.204	C
HETATM	133	H	0	24.497	34.224	35.603	H
HETATM	134	C	0	26.280	33.361	36.425	C
HETATM	135	H	0	26.085	32.366	36.035	H
HETATM	136	C	0	27.450	33.584	37.167	C
HETATM	137	O	0	28.387	32.641	37.410	O
HETATM	138	H	0	28.311	31.850	36.826	H
HETATM	139	C	0	27.668	34.862	37.711	C
HETATM	140	H	0	28.575	35.029	38.283	H
HETATM	141	C	0	26.764	35.892	37.479	C
HETATM	142	H	0	26.984	36.880	37.882	H
HETATM	143	C	0	22.425	31.109	38.386	C
HETATM	144	H	0	21.606	31.057	37.699	H
HETATM	145	H	0	23.347	31.024	37.849	H
HETATM	146	C	0	22.213	29.890	39.282	C
HETATM	147	O	0	21.440	29.917	40.253	O
HETATM	148	N	0	22.862	28.778	38.868	N
HETATM	149	H	0	23.580	28.840	38.149	H
HETATM	150	C	0	22.870	27.455	39.511	C
HETATM	151	H	0	23.195	27.558	40.558	H
HETATM	152	C	0	21.499	26.767	39.550	C
HETATM	153	H	0	21.029	26.867	38.555	H
HETATM	154	H	0	21.675	25.704	39.730	H
HETATM	155	O	0	20.662	27.246	40.585	O
HETATM	156	H	0	20.749	28.219	40.588	H
HETATM	157	C	0	23.977	26.614	38.789	C
HETATM	158	O	0	23.968	25.366	38.943	O
HETATM	159	O	0	24.818	27.287	38.129	O
HETATM	160	O	0	29.023	26.231	38.271	O
HETATM	161	C	0	29.162	27.519	37.965	C
HETATM	162	N	0	27.778	28.214	37.821	N
HETATM	163	H	0	26.982	27.571	37.806	H
HETATM	164	C	0	27.506	29.359	36.938	C
HETATM	165	O	0	26.354	29.780	36.926	O
HETATM	166	N	0	28.550	29.972	36.327	N
HETATM	167	C	0	29.704	29.318	36.260	C
HETATM	168	N	0	30.843	29.849	35.857	N
HETATM	169	C	0	31.840	28.898	36.073	C
HETATM	170	O	0	33.042	29.059	35.867	O
HETATM	171	N	0	31.298	27.753	36.649	N
HETATM	172	H	0	31.808	26.882	36.585	H
HETATM	173	C	0	29.866	27.824	36.615	C
HETATM	174	O	0	29.357	26.995	35.587	O
HETATM	175	H	0	28.403	27.190	35.406	H
HETATM	176	H	0	30.601	32.725	42.803	H
HETATM	177	H	0	32.313	33.020	42.618	H
HETATM	178	H	0	34.674	29.586	45.533	H
HETATM	179	H	0	33.079	29.743	46.229	H

HETATM	180	H	0	23.862	32.312	42.288	H
HETATM	181	H	0	23.226	31.066	43.333	H
HETATM	182	H	0	34.106	35.542	31.116	H
HETATM	183	H	0	35.491	34.831	31.910	H
HETATM	184	H	0	35.560	28.591	31.733	H
HETATM	185	H	0	36.001	26.904	31.844	H
HETATM	186	H	0	25.202	23.563	30.220	H
HETATM	187	H	0	24.717	24.898	31.238	H
HETATM	188	H	0	28.320	28.736	30.685	H
HETATM	189	H	0	26.593	28.864	30.916	H
HETATM	190	H	0	22.398	32.045	38.903	H
HETATM	191	H	0	36.436	30.487	34.771	H
HETATM	192	O	0	29.778	28.192	39.057	O
HETATM	193	H	0	30.425	28.903	38.857	H
HETATM	194	H	0	27.716	28.839	38.961	H
HETATM	195	H	0	24.782	37.770	38.312	H
HETATM	196	H	0	23.209	37.126	37.906	H
HETATM	197	H	0	27.136	30.542	42.909	H

PROa

HETATM	1	C	0	-1.996	-0.592	-6.063	C
HETATM	2	H	0	-2.105	-1.627	-6.312	H
HETATM	3	C	0	-1.691	-0.438	-4.564	C
HETATM	4	H	0	-0.816	-1.042	-4.303	H
HETATM	5	H	0	-1.402	0.599	-4.347	H
HETATM	6	C	0	-2.818	-0.827	-3.663	C
HETATM	7	N	0	-2.589	-1.361	-2.406	N
HETATM	8	C	0	-3.773	-1.506	-1.836	C
HETATM	9	H	0	-3.973	-1.828	-0.822	H
HETATM	10	N	0	-4.760	-1.125	-2.680	N
HETATM	11	H	0	-5.734	-1.052	-2.432	H
HETATM	12	C	0	-4.170	-0.679	-3.850	C
HETATM	13	H	0	-4.748	-0.292	-4.673	H
HETATM	14	C	0	-3.975	-4.729	-6.235	C
HETATM	15	H	0	-3.081	-4.204	-6.499	H
HETATM	16	C	0	-3.774	-5.583	-4.959	C
HETATM	17	H	0	-4.580	-6.328	-4.904	H
HETATM	18	H	0	-2.840	-6.156	-5.033	H
HETATM	19	C	0	-3.784	-4.765	-3.654	C
HETATM	20	H	0	-2.986	-4.013	-3.666	H
HETATM	21	H	0	-4.720	-4.197	-3.582	H
HETATM	22	C	0	-3.658	-5.667	-2.409	C
HETATM	23	H	0	-4.450	-6.423	-2.431	H
HETATM	24	H	0	-2.711	-6.218	-2.431	H
HETATM	25	N	0	-3.764	-4.992	-1.113	N
HETATM	26	H	0	-4.662	-4.950	-0.651	H
HETATM	27	C	0	-2.739	-4.430	-0.436	C
HETATM	28	N	0	-1.512	-4.360	-1.004	N
HETATM	29	H	0	-1.505	-4.336	-2.013	H
HETATM	30	H	0	-0.898	-3.629	-0.623	H
HETATM	31	N	0	-2.924	-4.018	0.810	N
HETATM	32	H	0	-3.890	-3.914	1.190	H
HETATM	33	H	0	-2.195	-3.426	1.205	H
HETATM	34	C	0	5.716	-1.326	-5.386	C
HETATM	35	H	0	5.012	-1.561	-6.156	H
HETATM	36	C	0	4.967	-1.436	-4.046	C
HETATM	37	H	0	5.656	-1.295	-3.211	H

HETATM	38	H	0	4.234	-0.621	-3.975	H
HETATM	39	C	0	4.260	-2.753	-3.867	C
HETATM	40	N	0	3.646	-3.406	-4.924	N
HETATM	41	C	0	3.111	-4.493	-4.412	C
HETATM	42	H	0	2.557	-5.246	-4.958	H
HETATM	43	N	0	3.338	-4.582	-3.069	N
HETATM	44	H	0	3.054	-5.326	-2.453	H
HETATM	45	C	0	4.078	-3.472	-2.705	C
HETATM	46	H	0	4.377	-3.312	-1.679	H
HETATM	47	C	0	5.347	6.740	-5.361	C
HETATM	48	H	0	5.900	7.643	-5.516	H
HETATM	49	C	0	4.608	6.858	-4.010	C
HETATM	50	H	0	5.346	6.906	-3.200	H
HETATM	51	H	0	4.079	7.821	-3.994	H
HETATM	52	C	0	3.623	5.746	-3.729	C
HETATM	53	C	0	3.893	4.741	-2.793	C
HETATM	54	H	0	4.840	4.756	-2.256	H
HETATM	55	C	0	2.979	3.725	-2.519	C
HETATM	56	H	0	3.208	2.954	-1.791	H
HETATM	57	C	0	1.744	3.696	-3.187	C
HETATM	58	O	0	0.809	2.751	-2.960	O
HETATM	59	H	0	1.047	2.222	-2.154	H
HETATM	60	C	0	1.465	4.689	-4.141	C
HETATM	61	H	0	0.504	4.664	-4.645	H
HETATM	62	C	0	2.392	5.692	-4.399	C
HETATM	63	H	0	2.146	6.465	-5.126	H
HETATM	64	C	0	6.586	1.121	-1.597	C
HETATM	65	H	0	7.034	1.596	-0.749	H
HETATM	66	H	0	5.525	1.247	-1.557	H
HETATM	67	C	0	7.046	-0.308	-1.359	C
HETATM	68	O	0	8.142	-0.712	-1.766	O
HETATM	69	N	0	6.242	-1.001	-0.505	N
HETATM	70	H	0	5.272	-0.685	-0.465	H
HETATM	71	C	0	6.571	-2.311	0.087	C
HETATM	72	H	0	6.539	-3.093	-0.682	H
HETATM	73	C	0	7.972	-2.369	0.737	C
HETATM	74	H	0	8.156	-1.416	1.260	H
HETATM	75	H	0	7.951	-3.149	1.506	H
HETATM	76	O	0	9.001	-2.709	-0.163	O
HETATM	77	H	0	8.912	-2.065	-0.896	H
HETATM	78	C	0	5.463	-2.621	1.121	C
HETATM	79	O	0	4.454	-3.251	0.687	O
HETATM	80	O	0	5.621	-2.212	2.311	O
HETATM	81	C	0	2.069	3.359	5.280	C
HETATM	82	H	0	2.281	2.554	5.952	H
HETATM	83	C	0	2.026	2.939	3.778	C
HETATM	84	H	0	1.025	2.653	3.457	H
HETATM	85	H	0	2.244	3.833	3.180	H
HETATM	86	C	0	2.944	1.832	3.344	C
HETATM	87	N	0	2.454	0.758	2.613	N
HETATM	88	C	0	3.492	-0.007	2.320	C
HETATM	89	H	0	3.453	-0.914	1.744	H
HETATM	90	N	0	4.630	0.505	2.824	N
HETATM	91	H	0	5.518	0.023	2.736	H
HETATM	92	C	0	4.306	1.678	3.481	C
HETATM	93	H	0	5.053	2.297	3.952	H
HETATM	94	C	0	4.152	0.599	8.069	C

HETATM	95	H	0	3.890	1.304	7.309	H
HETATM	96	C	0	3.782	-0.826	7.582	C
HETATM	97	H	0	4.372	-1.568	8.136	H
HETATM	98	H	0	2.733	-1.033	7.835	H
HETATM	99	C	0	3.959	-1.043	6.068	C
HETATM	100	H	0	3.243	-0.417	5.520	H
HETATM	101	H	0	4.953	-0.711	5.746	H
HETATM	102	C	0	3.767	-2.520	5.667	C
HETATM	103	H	0	4.633	-3.102	6.004	H
HETATM	104	H	0	2.901	-2.943	6.187	H
HETATM	105	N	0	3.601	-2.768	4.236	N
HETATM	106	H	0	4.417	-2.667	3.597	H
HETATM	107	C	0	2.434	-3.046	3.643	C
HETATM	108	N	0	1.252	-2.955	4.325	N
HETATM	109	H	0	1.216	-2.223	5.021	H
HETATM	110	H	0	0.441	-2.926	3.709	H
HETATM	111	N	0	2.409	-3.488	2.390	N
HETATM	112	H	0	3.275	-3.475	1.782	H
HETATM	113	H	0	1.508	-3.476	1.926	H
HETATM	114	C	0	-5.373	2.493	5.559	C
HETATM	115	H	0	-4.602	2.699	6.273	H
HETATM	116	C	0	-4.847	1.458	4.547	C
HETATM	117	H	0	-4.823	0.460	5.007	H
HETATM	118	H	0	-5.524	1.383	3.699	H
HETATM	119	C	0	-3.478	1.818	4.071	C
HETATM	120	N	0	-2.394	1.686	4.919	N
HETATM	121	C	0	-1.285	2.115	4.261	C
HETATM	122	H	0	-0.311	2.089	4.721	H
HETATM	123	N	0	-1.571	2.513	3.043	N
HETATM	124	C	0	-2.939	2.344	2.920	C
HETATM	125	H	0	-3.448	2.590	1.999	H
HETATM	126	C	0	-5.410	8.726	0.263	C
HETATM	127	H	0	-5.712	9.688	-0.094	H
HETATM	128	C	0	-4.774	7.969	-0.937	C
HETATM	129	H	0	-5.573	7.539	-1.553	H
HETATM	130	H	0	-4.282	8.716	-1.576	H
HETATM	131	C	0	-3.754	6.892	-0.616	C
HETATM	132	C	0	-3.970	5.541	-0.910	C
HETATM	133	H	0	-4.932	5.235	-1.319	H
HETATM	134	C	0	-2.983	4.569	-0.723	C
HETATM	135	H	0	-3.174	3.528	-0.972	H
HETATM	136	C	0	-1.722	4.941	-0.228	C
HETATM	137	O	0	-0.695	4.090	-0.047	O
HETATM	138	H	0	-0.903	3.162	-0.310	H
HETATM	139	C	0	-1.499	6.292	0.097	C
HETATM	140	H	0	-0.521	6.573	0.478	H
HETATM	141	C	0	-2.495	7.240	-0.100	C
HETATM	142	H	0	-2.281	8.283	0.130	H
HETATM	143	C	0	-7.046	2.185	1.120	C
HETATM	144	H	0	-7.865	2.133	0.433	H
HETATM	145	H	0	-6.125	2.100	0.584	H
HETATM	146	C	0	-7.297	0.993	2.044	C
HETATM	147	O	0	-8.156	1.040	2.939	O
HETATM	148	N	0	-6.593	-0.112	1.722	N
HETATM	149	H	0	-5.819	-0.047	1.063	H
HETATM	150	C	0	-6.626	-1.430	2.368	C
HETATM	151	H	0	-6.299	-1.342	3.415	H

HETATM	152	C	0	-8.013	-2.089	2.403	C
HETATM	153	H	0	-8.476	-1.983	1.404	H
HETATM	154	H	0	-7.858	-3.156	2.584	H
HETATM	155	O	0	-8.844	-1.596	3.432	O
HETATM	156	H	0	-8.777	-0.619	3.391	H
HETATM	157	C	0	-5.550	-2.290	1.631	C
HETATM	158	O	0	-5.555	-3.540	1.775	O
HETATM	159	O	0	-4.715	-1.631	0.950	O
HETATM	160	O	0	-0.389	-2.627	1.562	O
HETATM	161	C	0	0.023	-1.477	1.346	C
HETATM	162	N	0	-2.250	-0.148	1.065	N
HETATM	163	H	0	-3.191	-0.462	1.295	H
HETATM	164	C	0	-2.235	0.776	0.024	C
HETATM	165	O	0	-3.277	1.167	-0.504	O
HETATM	166	N	0	-0.994	1.252	-0.381	N
HETATM	167	C	0	0.113	0.556	-0.391	C
HETATM	168	N	0	1.277	1.089	-0.799	N
HETATM	169	C	0	2.271	0.152	-0.725	C
HETATM	170	O	0	3.460	0.292	-1.021	O
HETATM	171	N	0	1.776	-1.053	-0.182	N
HETATM	172	H	0	2.198	-1.908	-0.526	H
HETATM	173	C	0	0.328	-0.989	-0.090	C
HETATM	174	O	0	-0.264	-1.833	-1.034	O
HETATM	175	H	0	-1.097	-1.479	-1.468	H
HETATM	176	H	0	1.130	3.801	5.538	H
HETATM	177	H	0	2.842	4.096	5.352	H
HETATM	178	H	0	5.202	0.661	8.267	H
HETATM	179	H	0	3.607	0.819	8.963	H
HETATM	180	H	0	-5.609	3.388	5.022	H
HETATM	181	H	0	-6.246	2.141	6.068	H
HETATM	182	H	0	4.635	6.618	-6.149	H
HETATM	183	H	0	6.020	5.907	-5.356	H
HETATM	184	H	0	6.088	-0.334	-5.533	H
HETATM	185	H	0	6.530	-2.021	-5.422	H
HETATM	186	H	0	-4.270	-5.361	-7.046	H
HETATM	187	H	0	-4.754	-4.026	-6.028	H
HETATM	188	H	0	-1.151	-0.188	-6.581	H
HETATM	189	H	0	-2.878	-0.060	-6.350	H
HETATM	190	H	0	-7.073	3.121	1.637	H
HETATM	191	H	0	6.965	1.563	-2.495	H
HETATM	192	O	0	0.352	-0.717	2.370	O
HETATM	193	H	0	1.074	0.033	2.276	H
HETATM	194	H	0	-1.695	0.137	1.863	H
HETATM	195	H	0	-4.690	8.845	1.046	H
HETATM	196	H	0	-6.263	8.201	0.640	H
HETATM	197	H	0	-2.429	1.397	5.877	H

Pathway 2
TS1b

HETATM	1	C	0	27.476	28.332	31.203	C
HETATM	2	H	0	27.366	27.297	30.953	H
HETATM	3	C	0	27.722	28.516	32.710	C
HETATM	4	H	0	28.572	27.910	33.036	H
HETATM	5	H	0	28.027	29.554	32.905	H
HETATM	6	C	0	26.541	28.190	33.572	C
HETATM	7	N	0	26.704	27.704	34.858	N
HETATM	8	C	0	25.498	27.611	35.392	C

HETATM	9	H	0	25.261	27.305	36.406	H
HETATM	10	N	0	24.556	27.994	34.495	N
HETATM	11	H	0	23.568	28.062	34.683	H
HETATM	12	C	0	25.201	28.370	33.329	C
HETATM	13	H	0	24.662	28.737	32.471	H
HETATM	14	C	0	25.497	24.195	31.031	C
HETATM	15	H	0	26.391	24.721	30.767	H
HETATM	16	C	0	25.707	23.357	32.317	C
HETATM	17	H	0	24.908	22.606	32.386	H
HETATM	18	H	0	26.646	22.791	32.249	H
HETATM	19	C	0	25.693	24.195	33.612	C
HETATM	20	H	0	26.475	24.963	33.583	H
HETATM	21	H	0	24.744	24.741	33.682	H
HETATM	22	C	0	25.853	23.319	34.870	C
HETATM	23	H	0	25.118	22.506	34.836	H
HETATM	24	H	0	26.839	22.840	34.880	H
HETATM	25	N	0	25.669	24.000	36.156	N
HETATM	26	H	0	24.748	24.007	36.574	H
HETATM	27	C	0	26.660	24.455	36.956	C
HETATM	28	N	0	27.918	24.575	36.470	N
HETATM	29	H	0	27.991	24.785	35.485	H
HETATM	30	H	0	28.522	25.173	37.045	H
HETATM	31	N	0	26.424	24.722	38.231	N
HETATM	32	H	0	25.442	24.832	38.570	H
HETATM	33	H	0	27.140	25.305	38.681	H
HETATM	34	C	0	35.188	27.598	31.880	C
HETATM	35	H	0	34.484	27.363	31.109	H
HETATM	36	C	0	34.499	27.524	33.259	C
HETATM	37	H	0	35.124	26.975	33.969	H
HETATM	38	H	0	34.413	28.538	33.670	H
HETATM	39	C	0	33.113	26.943	33.267	C
HETATM	40	N	0	32.275	26.979	32.167	N
HETATM	41	C	0	31.103	26.571	32.608	C
HETATM	42	H	0	30.205	26.479	32.012	H
HETATM	43	N	0	31.138	26.235	33.932	N
HETATM	44	H	0	30.342	26.222	34.563	H
HETATM	45	C	0	32.428	26.478	34.363	C
HETATM	46	H	0	32.746	26.295	35.379	H
HETATM	47	C	0	34.819	35.664	31.905	C
HETATM	48	H	0	35.371	36.567	31.750	H
HETATM	49	C	0	34.084	35.790	33.259	C
HETATM	50	H	0	34.826	35.821	34.067	H
HETATM	51	H	0	33.576	36.764	33.280	H
HETATM	52	C	0	33.075	34.700	33.542	C
HETATM	53	C	0	33.320	33.692	34.481	C
HETATM	54	H	0	34.270	33.682	35.014	H
HETATM	55	C	0	32.380	32.703	34.762	C
HETATM	56	H	0	32.590	31.927	35.492	H
HETATM	57	C	0	31.142	32.706	34.098	C
HETATM	58	O	0	30.178	31.794	34.337	O
HETATM	59	H	0	30.410	31.251	35.135	H
HETATM	60	C	0	30.888	33.702	33.141	C
HETATM	61	H	0	29.926	33.700	32.637	H
HETATM	62	C	0	31.841	34.678	32.876	C
HETATM	63	H	0	31.614	35.454	32.146	H
HETATM	64	C	0	36.058	30.045	35.669	C
HETATM	65	H	0	36.506	30.520	36.517	H

HETATM	66	H	0	34.996	30.171	35.709	H
HETATM	67	C	0	36.430	28.583	35.878	C
HETATM	68	O	0	37.508	28.123	35.484	O
HETATM	69	N	0	35.541	27.917	36.670	N
HETATM	70	H	0	34.595	28.302	36.692	H
HETATM	71	C	0	35.686	26.533	37.171	C
HETATM	72	H	0	35.497	25.818	36.360	H
HETATM	73	C	0	37.076	26.216	37.760	C
HETATM	74	H	0	37.402	27.074	38.371	H
HETATM	75	H	0	36.956	25.372	38.446	H
HETATM	76	O	0	38.032	25.832	36.799	O
HETATM	77	H	0	38.050	26.574	36.160	H
HETATM	78	C	0	34.573	26.346	38.236	C
HETATM	79	O	0	33.434	25.990	37.810	O
HETATM	80	O	0	34.859	26.599	39.444	O
HETATM	81	C	0	31.541	32.283	42.546	C
HETATM	82	H	0	31.753	31.478	43.218	H
HETATM	83	C	0	31.333	31.753	41.108	C
HETATM	84	H	0	30.342	31.290	41.069	H
HETATM	85	H	0	31.295	32.596	40.407	H
HETATM	86	C	0	32.310	30.735	40.602	C
HETATM	87	N	0	31.866	29.625	39.893	N
HETATM	88	C	0	32.914	28.907	39.519	C
HETATM	89	H	0	32.877	27.999	38.947	H
HETATM	90	N	0	34.030	29.494	39.963	N
HETATM	91	H	0	34.940	29.071	39.826	H
HETATM	92	C	0	33.680	30.649	40.646	C
HETATM	93	H	0	34.416	31.306	41.080	H
HETATM	94	C	0	33.624	29.523	45.335	C
HETATM	95	H	0	33.362	30.228	44.575	H
HETATM	96	C	0	33.248	28.103	44.843	C
HETATM	97	H	0	33.833	27.353	45.392	H
HETATM	98	H	0	32.198	27.901	45.097	H
HETATM	99	C	0	33.418	27.892	43.327	C
HETATM	100	H	0	32.742	28.570	42.791	H
HETATM	101	H	0	34.431	28.166	43.009	H
HETATM	102	C	0	33.129	26.434	42.907	C
HETATM	103	H	0	33.979	25.801	43.179	H
HETATM	104	H	0	32.276	26.046	43.478	H
HETATM	105	N	0	32.861	26.207	41.490	N
HETATM	106	H	0	33.649	26.157	40.820	H
HETATM	107	C	0	31.629	26.275	40.945	C
HETATM	108	N	0	30.553	26.715	41.659	N
HETATM	109	H	0	30.784	27.251	42.482	H
HETATM	110	H	0	29.894	27.220	41.041	H
HETATM	111	N	0	31.427	25.874	39.699	N
HETATM	112	H	0	32.233	25.742	39.055	H
HETATM	113	H	0	30.497	26.037	39.286	H
HETATM	114	C	0	24.033	31.460	42.887	C
HETATM	115	H	0	24.803	31.665	43.601	H
HETATM	116	C	0	24.552	30.379	41.892	C
HETATM	117	H	0	24.047	29.424	42.080	H
HETATM	118	H	0	24.301	30.647	40.865	H
HETATM	119	C	0	26.030	30.182	41.968	C
HETATM	120	N	0	26.621	29.475	43.003	N
HETATM	121	C	0	27.971	29.516	42.824	C
HETATM	122	H	0	28.662	29.028	43.497	H

HETATM	123	N	0	28.287	30.210	41.750	N
HETATM	124	C	0	27.085	30.633	41.211	C
HETATM	125	H	0	27.056	31.227	40.308	H
HETATM	126	C	0	24.062	37.650	37.529	C
HETATM	127	H	0	23.760	38.612	37.172	H
HETATM	128	C	0	24.743	36.853	36.388	C
HETATM	129	H	0	23.984	36.458	35.701	H
HETATM	130	H	0	25.348	37.554	35.796	H
HETATM	131	C	0	25.636	35.725	36.870	C
HETATM	132	C	0	25.392	34.383	36.554	C
HETATM	133	H	0	24.517	34.130	35.957	H
HETATM	134	C	0	26.239	33.356	36.977	C
HETATM	135	H	0	26.028	32.318	36.741	H
HETATM	136	C	0	27.378	33.661	37.739	C
HETATM	137	O	0	28.244	32.724	38.191	O
HETATM	138	H	0	28.198	31.885	37.656	H
HETATM	139	C	0	27.632	35.001	38.072	C
HETATM	140	H	0	28.521	35.229	38.651	H
HETATM	141	C	0	26.776	36.007	37.640	C
HETATM	142	H	0	27.010	37.040	37.892	H
HETATM	143	C	0	22.425	31.109	38.386	C
HETATM	144	H	0	21.606	31.057	37.699	H
HETATM	145	H	0	23.347	31.024	37.849	H
HETATM	146	C	0	22.196	29.912	39.312	C
HETATM	147	O	0	21.288	29.938	40.158	O
HETATM	148	N	0	22.959	28.829	39.043	N
HETATM	149	H	0	23.802	28.923	38.475	H
HETATM	150	C	0	22.867	27.502	39.668	C
HETATM	151	H	0	23.173	27.558	40.725	H
HETATM	152	C	0	21.458	26.885	39.667	C
HETATM	153	H	0	21.004	27.050	38.672	H
HETATM	154	H	0	21.585	25.807	39.798	H
HETATM	155	O	0	20.633	27.347	40.715	O
HETATM	156	H	0	20.687	28.325	40.675	H
HETATM	157	C	0	23.924	26.596	38.957	C
HETATM	158	O	0	23.830	25.346	39.086	O
HETATM	159	O	0	24.827	27.210	38.325	O
HETATM	160	O	0	28.766	26.357	38.622	O
HETATM	161	C	0	28.736	27.583	38.260	C
HETATM	162	N	0	27.490	28.229	38.186	N
HETATM	163	H	0	26.655	27.764	38.548	H
HETATM	164	C	0	27.248	29.500	37.638	C
HETATM	165	O	0	26.116	29.980	37.709	O
HETATM	166	N	0	28.301	30.199	37.074	N
HETATM	167	C	0	29.438	29.566	36.876	C
HETATM	168	N	0	30.588	30.125	36.502	N
HETATM	169	C	0	31.575	29.164	36.659	C
HETATM	170	O	0	32.784	29.320	36.465	O
HETATM	171	N	0	31.028	27.985	37.191	N
HETATM	172	H	0	31.543	27.124	37.056	H
HETATM	173	C	0	29.605	28.049	37.078	C
HETATM	174	O	0	29.184	27.291	35.940	O
HETATM	175	H	0	28.251	27.519	35.649	H
HETATM	176	H	0	30.601	32.725	42.803	H
HETATM	177	H	0	32.313	33.020	42.618	H
HETATM	178	H	0	34.674	29.586	45.533	H
HETATM	179	H	0	33.079	29.743	46.229	H

HETATM	180	H	0	23.796	32.355	42.350	H
HETATM	181	H	0	23.160	31.108	43.396	H
HETATM	182	H	0	34.106	35.542	31.116	H
HETATM	183	H	0	35.491	34.831	31.910	H
HETATM	184	H	0	35.560	28.591	31.733	H
HETATM	185	H	0	36.001	26.904	31.844	H
HETATM	186	H	0	25.202	23.563	30.220	H
HETATM	187	H	0	24.717	24.898	31.238	H
HETATM	188	H	0	28.320	28.736	30.685	H
HETATM	189	H	0	26.593	28.864	30.916	H
HETATM	190	H	0	22.398	32.045	38.903	H
HETATM	191	H	0	36.436	30.487	34.771	H
HETATM	192	O	0	29.607	28.494	39.640	O
HETATM	193	H	0	30.805	29.225	39.717	H
HETATM	194	H	0	28.935	29.071	40.042	H
HETATM	195	H	0	24.782	37.770	38.312	H
HETATM	196	H	0	23.209	37.126	37.906	H
HETATM	197	H	0	26.129	28.984	43.734	H

INT1b

HETATM	1	C	0	27.476	28.332	31.203	C
HETATM	2	H	0	27.366	27.297	30.953	H
HETATM	3	C	0	27.743	28.499	32.708	C
HETATM	4	H	0	28.604	27.892	33.007	H
HETATM	5	H	0	28.040	29.536	32.915	H
HETATM	6	C	0	26.581	28.141	33.582	C
HETATM	7	N	0	26.766	27.529	34.810	N
HETATM	8	C	0	25.575	27.448	35.379	C
HETATM	9	H	0	25.358	27.093	36.378	H
HETATM	10	N	0	24.621	27.949	34.556	N
HETATM	11	H	0	23.649	28.063	34.795	H
HETATM	12	C	0	25.244	28.402	33.406	C
HETATM	13	H	0	24.697	28.873	32.606	H
HETATM	14	C	0	25.497	24.195	31.031	C
HETATM	15	H	0	26.391	24.721	30.767	H
HETATM	16	C	0	25.719	23.350	32.312	C
HETATM	17	H	0	24.917	22.602	32.388	H
HETATM	18	H	0	26.653	22.779	32.223	H
HETATM	19	C	0	25.734	24.176	33.614	C
HETATM	20	H	0	26.501	24.959	33.568	H
HETATM	21	H	0	24.778	24.705	33.719	H
HETATM	22	C	0	25.952	23.296	34.862	C
HETATM	23	H	0	25.241	22.461	34.842	H
HETATM	24	H	0	26.950	22.845	34.844	H
HETATM	25	N	0	25.782	23.962	36.158	N
HETATM	26	H	0	24.860	23.984	36.572	H
HETATM	27	C	0	26.774	24.439	36.946	C
HETATM	28	N	0	28.037	24.539	36.475	N
HETATM	29	H	0	28.124	24.710	35.484	H
HETATM	30	H	0	28.625	25.172	37.041	H
HETATM	31	N	0	26.537	24.759	38.211	N
HETATM	32	H	0	25.561	24.873	38.555	H
HETATM	33	H	0	27.267	25.349	38.633	H
HETATM	34	C	0	35.188	27.598	31.880	C
HETATM	35	H	0	34.484	27.363	31.109	H
HETATM	36	C	0	34.485	27.529	33.250	C
HETATM	37	H	0	35.140	27.070	33.996	H

HETATM	38	H	0	34.298	28.548	33.611		H
HETATM	39	C	0	33.157	26.827	33.258		C
HETATM	40	N	0	32.301	26.840	32.169		N
HETATM	41	C	0	31.171	26.330	32.610		C
HETATM	42	H	0	30.270	26.197	32.025		H
HETATM	43	N	0	31.254	25.941	33.918		N
HETATM	44	H	0	30.469	25.877	34.556		H
HETATM	45	C	0	32.528	26.270	34.342		C
HETATM	46	H	0	32.869	26.085	35.351		H
HETATM	47	C	0	34.819	35.664	31.905		C
HETATM	48	H	0	35.371	36.567	31.750		H
HETATM	49	C	0	34.075	35.792	33.253		C
HETATM	50	H	0	34.808	35.798	34.070		H
HETATM	51	H	0	33.584	36.774	33.279		H
HETATM	52	C	0	33.046	34.715	33.498		C
HETATM	53	C	0	33.299	33.631	34.345		C
HETATM	54	H	0	34.262	33.564	34.849		H
HETATM	55	C	0	32.353	32.633	34.565		C
HETATM	56	H	0	32.570	31.798	35.223		H
HETATM	57	C	0	31.100	32.706	33.933		C
HETATM	58	O	0	30.135	31.784	34.118		O
HETATM	59	H	0	30.394	31.176	34.862		H
HETATM	60	C	0	30.834	33.783	33.072		C
HETATM	61	H	0	29.860	33.836	32.595		H
HETATM	62	C	0	31.795	34.766	32.865		C
HETATM	63	H	0	31.564	35.602	32.207		H
HETATM	64	C	0	36.058	30.045	35.669		C
HETATM	65	H	0	36.506	30.520	36.517		H
HETATM	66	H	0	34.996	30.171	35.709		H
HETATM	67	C	0	36.465	28.594	35.886		C
HETATM	68	O	0	37.566	28.169	35.512		O
HETATM	69	N	0	35.590	27.903	36.667		N
HETATM	70	H	0	34.629	28.251	36.662		H
HETATM	71	C	0	35.797	26.546	37.212		C
HETATM	72	H	0	35.682	25.798	36.417		H
HETATM	73	C	0	37.183	26.330	37.855		C
HETATM	74	H	0	37.443	27.227	38.441		H
HETATM	75	H	0	37.088	25.507	38.569		H
HETATM	76	O	0	38.188	25.967	36.936		O
HETATM	77	H	0	38.176	26.681	36.264		H
HETATM	78	C	0	34.652	26.325	38.236		C
HETATM	79	O	0	33.527	26.002	37.747		O
HETATM	80	O	0	34.896	26.519	39.461		O
HETATM	81	C	0	31.541	32.283	42.546		C
HETATM	82	H	0	31.753	31.478	43.218		H
HETATM	83	C	0	31.401	31.788	41.087		C
HETATM	84	H	0	30.416	31.333	40.947		H
HETATM	85	H	0	31.397	32.659	40.419		H
HETATM	86	C	0	32.403	30.781	40.602		C
HETATM	87	N	0	31.983	29.727	39.805		N
HETATM	88	C	0	33.066	29.044	39.491		C
HETATM	89	H	0	33.090	28.162	38.876		H
HETATM	90	N	0	34.170	29.591	40.048		N
HETATM	91	H	0	35.093	29.188	39.963		H
HETATM	92	C	0	33.769	30.705	40.763		C
HETATM	93	H	0	34.471	31.335	41.287		H
HETATM	94	C	0	33.624	29.523	45.335		C

HETATM	95	H	0	33.362	30.228	44.575	H
HETATM	96	C	0	33.233	28.106	44.823	C
HETATM	97	H	0	33.856	27.347	45.313	H
HETATM	98	H	0	32.203	27.885	45.135	H
HETATM	99	C	0	33.311	27.936	43.291	C
HETATM	100	H	0	32.619	28.637	42.808	H
HETATM	101	H	0	34.309	28.208	42.925	H
HETATM	102	C	0	32.981	26.498	42.832	C
HETATM	103	H	0	33.794	25.827	43.135	H
HETATM	104	H	0	32.085	26.136	43.347	H
HETATM	105	N	0	32.780	26.325	41.394	N
HETATM	106	H	0	33.602	26.312	40.757	H
HETATM	107	C	0	31.588	26.158	40.797	C
HETATM	108	N	0	30.417	26.325	41.497	N
HETATM	109	H	0	30.444	27.051	42.199	H
HETATM	110	H	0	29.621	26.419	40.867	H
HETATM	111	N	0	31.494	25.765	39.540	N
HETATM	112	H	0	32.342	25.726	38.929	H
HETATM	113	H	0	30.567	25.888	39.087	H
HETATM	114	C	0	24.099	31.418	42.825	C
HETATM	115	H	0	24.869	31.623	43.538	H
HETATM	116	C	0	24.519	30.257	41.909	C
HETATM	117	H	0	24.210	29.312	42.375	H
HETATM	118	H	0	23.988	30.297	40.960	H
HETATM	119	C	0	25.972	30.164	41.617	C
HETATM	120	N	0	26.955	30.163	42.601	N
HETATM	121	C	0	28.148	29.906	42.030	C
HETATM	122	H	0	29.090	29.847	42.550	H
HETATM	123	N	0	27.964	29.752	40.728	N
HETATM	124	C	0	26.626	29.911	40.446	C
HETATM	125	H	0	26.235	29.834	39.438	H
HETATM	126	C	0	24.062	37.650	37.529	C
HETATM	127	H	0	23.760	38.612	37.172	H
HETATM	128	C	0	24.723	36.860	36.370	C
HETATM	129	H	0	23.948	36.495	35.685	H
HETATM	130	H	0	25.330	37.565	35.784	H
HETATM	131	C	0	25.611	35.702	36.791	C
HETATM	132	C	0	25.369	34.383	36.386	C
HETATM	133	H	0	24.481	34.166	35.795	H
HETATM	134	C	0	26.238	33.335	36.703	C
HETATM	135	H	0	26.028	32.315	36.393	H
HETATM	136	C	0	27.403	33.592	37.443	C
HETATM	137	O	0	28.306	32.647	37.778	O
HETATM	138	H	0	28.230	31.822	37.229	H
HETATM	139	C	0	27.647	34.906	37.879	C
HETATM	140	H	0	28.553	35.101	38.444	H
HETATM	141	C	0	26.768	35.933	37.553	C
HETATM	142	H	0	27.004	36.946	37.875	H
HETATM	143	C	0	22.425	31.109	38.386	C
HETATM	144	H	0	21.606	31.057	37.699	H
HETATM	145	H	0	23.347	31.024	37.849	H
HETATM	146	C	0	22.205	29.911	39.309	C
HETATM	147	O	0	21.334	29.940	40.194	O
HETATM	148	N	0	22.951	28.823	39.014	N
HETATM	149	H	0	23.756	28.898	38.390	H
HETATM	150	C	0	22.891	27.503	39.658	C
HETATM	151	H	0	23.180	27.589	40.717	H

HETATM	152	C	0	21.500	26.849	39.649	C
HETATM	153	H	0	21.056	26.984	38.646	H
HETATM	154	H	0	21.651	25.778	39.804	H
HETATM	155	O	0	20.650	27.314	40.678	O
HETATM	156	H	0	20.703	28.290	40.647	H
HETATM	157	C	0	23.990	26.624	38.972	C
HETATM	158	O	0	23.927	25.375	39.105	O
HETATM	159	O	0	24.881	27.270	38.355	O
HETATM	160	O	0	28.911	26.352	38.574	O
HETATM	161	C	0	28.941	27.629	38.199	C
HETATM	162	N	0	27.612	28.191	37.970	N
HETATM	163	H	0	26.794	27.695	38.319	H
HETATM	164	C	0	27.346	29.392	37.326	C
HETATM	165	O	0	26.201	29.875	37.377	O
HETATM	166	N	0	28.373	30.073	36.700	N
HETATM	167	C	0	29.529	29.452	36.564	C
HETATM	168	N	0	30.660	30.009	36.144	N
HETATM	169	C	0	31.681	29.102	36.393	C
HETATM	170	O	0	32.881	29.277	36.183	O
HETATM	171	N	0	31.167	27.958	37.024	N
HETATM	172	H	0	31.727	27.114	37.003	H
HETATM	173	C	0	29.741	27.962	36.913	C
HETATM	174	O	0	29.311	27.105	35.860	O
HETATM	175	H	0	28.378	27.320	35.577	H
HETATM	176	H	0	30.601	32.725	42.803	H
HETATM	177	H	0	32.313	33.020	42.618	H
HETATM	178	H	0	34.674	29.586	45.533	H
HETATM	179	H	0	33.079	29.743	46.229	H
HETATM	180	H	0	23.862	32.312	42.288	H
HETATM	181	H	0	23.226	31.066	43.333	H
HETATM	182	H	0	34.106	35.542	31.116	H
HETATM	183	H	0	35.491	34.831	31.910	H
HETATM	184	H	0	35.560	28.591	31.733	H
HETATM	185	H	0	36.001	26.904	31.844	H
HETATM	186	H	0	25.202	23.563	30.220	H
HETATM	187	H	0	24.717	24.898	31.238	H
HETATM	188	H	0	28.320	28.736	30.685	H
HETATM	189	H	0	26.593	28.864	30.916	H
HETATM	190	H	0	22.398	32.045	38.903	H
HETATM	191	H	0	36.436	30.487	34.771	H
HETATM	192	O	0	29.549	28.458	39.310	O
HETATM	193	H	0	30.447	28.855	39.165	H
HETATM	194	H	0	28.714	29.279	40.017	H
HETATM	195	H	0	24.782	37.770	38.312	H
HETATM	196	H	0	23.209	37.126	37.906	H
HETATM	197	H	0	26.805	30.334	43.584	H

TS2b

HETATM	1	C	0	-1.883	-0.719	-6.261	C
HETATM	2	H	0	-1.993	-1.753	-6.511	H
HETATM	3	C	0	-1.616	-0.531	-4.758	C
HETATM	4	H	0	-0.701	-1.061	-4.474	H
HETATM	5	H	0	-1.397	0.527	-4.561	H
HETATM	6	C	0	-2.713	-0.975	-3.838	C
HETATM	7	N	0	-2.415	-1.364	-2.544	N
HETATM	8	C	0	-3.573	-1.590	-1.947	C
HETATM	9	H	0	-3.702	-1.872	-0.917	H

HETATM	10	N	0	-4.606	-1.396	-2.787	N
HETATM	11	H	0	-5.566	-1.556	-2.475	H
HETATM	12	C	0	-4.080	-1.002	-4.003	C
HETATM	13	H	0	-4.707	-0.759	-4.846	H
HETATM	14	C	0	-3.862	-4.856	-6.433	C
HETATM	15	H	0	-2.968	-4.330	-6.697	H
HETATM	16	C	0	-3.677	-5.685	-5.140	C
HETATM	17	H	0	-4.505	-6.404	-5.066	H
HETATM	18	H	0	-2.760	-6.286	-5.193	H
HETATM	19	C	0	-3.673	-4.823	-3.862	C
HETATM	20	H	0	-2.802	-4.155	-3.852	H
HETATM	21	H	0	-4.547	-4.162	-3.864	H
HETATM	22	C	0	-3.714	-5.671	-2.577	C
HETATM	23	H	0	-4.581	-6.340	-2.617	H
HETATM	24	H	0	-2.833	-6.320	-2.510	H
HETATM	25	N	0	-3.829	-4.917	-1.328	N
HETATM	26	H	0	-4.749	-4.603	-0.980	H
HETATM	27	C	0	-2.799	-4.518	-0.572	C
HETATM	28	N	0	-1.513	-4.682	-0.969	N
HETATM	29	H	0	-1.363	-4.699	-1.967	H
HETATM	30	H	0	-0.848	-4.079	-0.480	H
HETATM	31	N	0	-3.039	-4.002	0.635	N
HETATM	32	H	0	-4.022	-3.827	0.882	H
HETATM	33	H	0	-2.271	-3.539	1.114	H
HETATM	34	C	0	5.829	-1.453	-5.584	C
HETATM	35	H	0	5.125	-1.687	-6.355	H
HETATM	36	C	0	5.146	-1.520	-4.199	C
HETATM	37	H	0	5.788	-2.044	-3.484	H
HETATM	38	H	0	5.043	-0.500	-3.806	H
HETATM	39	C	0	3.771	-2.126	-4.162	C
HETATM	40	N	0	2.897	-2.065	-5.232	N
HETATM	41	C	0	1.744	-2.499	-4.767	C
HETATM	42	H	0	0.829	-2.582	-5.337	H
HETATM	43	N	0	1.823	-2.876	-3.456	N
HETATM	44	H	0	1.042	-2.933	-2.808	H
HETATM	45	C	0	3.125	-2.633	-3.060	C
HETATM	46	H	0	3.482	-2.860	-2.067	H
HETATM	47	C	0	5.460	6.613	-5.559	C
HETATM	48	H	0	6.012	7.517	-5.714	H
HETATM	49	C	0	4.731	6.723	-4.198	C
HETATM	50	H	0	5.482	6.782	-3.400	H
HETATM	51	H	0	4.195	7.682	-4.176	H
HETATM	52	C	0	3.754	5.609	-3.881	C
HETATM	53	C	0	4.022	4.642	-2.903	C
HETATM	54	H	0	4.976	4.669	-2.379	H
HETATM	55	C	0	3.099	3.649	-2.569	C
HETATM	56	H	0	3.330	2.906	-1.811	H
HETATM	57	C	0	1.852	3.605	-3.215	C
HETATM	58	O	0	0.897	2.699	-2.935	O
HETATM	59	H	0	1.110	2.160	-2.127	H
HETATM	60	C	0	1.578	4.556	-4.214	C
HETATM	61	H	0	0.612	4.519	-4.708	H
HETATM	62	C	0	2.513	5.535	-4.532	C
HETATM	63	H	0	2.263	6.275	-5.291	H
HETATM	64	C	0	6.699	0.994	-1.795	C
HETATM	65	H	0	7.147	1.470	-0.947	H
HETATM	66	H	0	5.637	1.120	-1.755	H

HETATM	67	C	0	7.094	-0.457	-1.582	C
HETATM	68	O	0	8.161	-0.914	-2.008	O
HETATM	69	N	0	6.246	-1.119	-0.743	N
HETATM	70	H	0	5.298	-0.744	-0.691	H
HETATM	71	C	0	6.443	-2.492	-0.244	C
HETATM	72	H	0	6.283	-3.218	-1.049	H
HETATM	73	C	0	7.849	-2.753	0.349	C
HETATM	74	H	0	8.142	-1.872	0.944	H
HETATM	75	H	0	7.764	-3.592	1.049	H
HETATM	76	O	0	8.814	-3.120	-0.605	O
HETATM	77	H	0	8.788	-2.404	-1.274	H
HETATM	78	C	0	5.364	-2.741	0.833	C
HETATM	79	O	0	4.365	-3.429	0.485	O
HETATM	80	O	0	5.545	-2.239	1.989	O
HETATM	81	C	0	2.182	3.232	5.082	C
HETATM	82	H	0	2.394	2.427	5.754	H
HETATM	83	C	0	1.973	2.718	3.642	C
HETATM	84	H	0	0.971	2.279	3.602	H
HETATM	85	H	0	1.970	3.560	2.939	H
HETATM	86	C	0	2.922	1.670	3.151	C
HETATM	87	N	0	2.431	0.544	2.505	N
HETATM	88	C	0	3.434	-0.241	2.129	C
HETATM	89	H	0	3.333	-1.163	1.590	H
HETATM	90	N	0	4.572	0.324	2.519	N
HETATM	91	H	0	5.452	-0.165	2.357	H
HETATM	92	C	0	4.287	1.523	3.157	C
HETATM	93	H	0	5.060	2.167	3.542	H
HETATM	94	C	0	4.265	0.472	7.871	C
HETATM	95	H	0	4.003	1.178	7.111	H
HETATM	96	C	0	3.891	-0.954	7.393	C
HETATM	97	H	0	4.502	-1.693	7.928	H
HETATM	98	H	0	2.852	-1.170	7.676	H
HETATM	99	C	0	4.024	-1.169	5.875	C
HETATM	100	H	0	3.293	-0.537	5.356	H
HETATM	101	H	0	5.012	-0.842	5.526	H
HETATM	102	C	0	3.808	-2.642	5.475	C
HETATM	103	H	0	4.673	-3.235	5.792	H
HETATM	104	H	0	2.946	-3.055	6.010	H
HETATM	105	N	0	3.607	-2.881	4.047	N
HETATM	106	H	0	4.406	-2.795	3.393	H
HETATM	107	C	0	2.421	-3.163	3.480	C
HETATM	108	N	0	1.255	-3.029	4.180	N
HETATM	109	H	0	1.240	-2.275	4.853	H
HETATM	110	H	0	0.434	-3.001	3.577	H
HETATM	111	N	0	2.371	-3.630	2.242	N
HETATM	112	H	0	3.227	-3.653	1.623	H
HETATM	113	H	0	1.460	-3.614	1.786	H
HETATM	114	C	0	-5.326	2.409	5.423	C
HETATM	115	H	0	-4.555	2.615	6.137	H
HETATM	116	C	0	-4.726	1.350	4.441	C
HETATM	117	H	0	-5.234	0.387	4.561	H
HETATM	118	H	0	-4.913	1.644	3.409	H
HETATM	119	C	0	-3.248	1.177	4.610	C
HETATM	120	N	0	-2.704	0.472	5.674	N
HETATM	121	C	0	-1.344	0.537	5.573	C
HETATM	122	H	0	-0.687	0.065	6.290	H
HETATM	123	N	0	-0.975	1.238	4.522	N

HETATM	124	C	0	-2.153	1.641	3.917	C
HETATM	125	H	0	-2.139	2.237	3.014	H
HETATM	126	C	0	-5.297	8.599	0.065	C
HETATM	127	H	0	-5.599	9.562	-0.292	H
HETATM	128	C	0	-4.616	7.787	-1.068	C
HETATM	129	H	0	-5.374	7.403	-1.761	H
HETATM	130	H	0	-3.995	8.478	-1.657	H
HETATM	131	C	0	-3.742	6.642	-0.582	C
HETATM	132	C	0	-3.984	5.307	-0.930	C
HETATM	133	H	0	-4.850	5.069	-1.546	H
HETATM	134	C	0	-3.148	4.265	-0.519	C
HETATM	135	H	0	-3.358	3.232	-0.784	H
HETATM	136	C	0	-2.019	4.547	0.266	C
HETATM	137	O	0	-1.158	3.602	0.715	O
HETATM	138	H	0	-1.206	2.752	0.198	H
HETATM	139	C	0	-1.767	5.879	0.633	C
HETATM	140	H	0	-0.886	6.092	1.232	H
HETATM	141	C	0	-2.612	6.900	0.211	C
HETATM	142	H	0	-2.375	7.925	0.490	H
HETATM	143	C	0	-6.934	2.058	0.922	C
HETATM	144	H	0	-7.753	2.007	0.235	H
HETATM	145	H	0	-6.012	1.973	0.385	H
HETATM	146	C	0	-7.049	0.796	1.773	C
HETATM	147	O	0	-7.331	0.796	2.981	O
HETATM	148	N	0	-6.834	-0.316	1.031	N
HETATM	149	H	0	-6.658	-0.207	0.038	H
HETATM	150	C	0	-6.553	-1.686	1.454	C
HETATM	151	H	0	-5.632	-1.707	2.054	H
HETATM	152	C	0	-7.645	-2.328	2.330	C
HETATM	153	H	0	-8.620	-2.185	1.832	H
HETATM	154	H	0	-7.441	-3.402	2.371	H
HETATM	155	O	0	-7.644	-1.847	3.657	O
HETATM	156	H	0	-7.640	-0.867	3.592	H
HETATM	157	C	0	-6.275	-2.513	0.158	C
HETATM	158	O	0	-5.746	-3.655	0.340	O
HETATM	159	O	0	-6.580	-1.989	-0.938	O
HETATM	160	O	0	-0.337	-2.979	1.305	O
HETATM	161	C	0	-0.181	-1.754	1.123	C
HETATM	162	N	0	-2.069	-0.643	1.134	N
HETATM	163	H	0	-2.981	-0.992	1.426	H
HETATM	164	C	0	-2.256	0.390	0.262	C
HETATM	165	O	0	-3.357	0.873	-0.041	O
HETATM	166	N	0	-1.078	0.995	-0.250	N
HETATM	167	C	0	0.033	0.345	-0.419	C
HETATM	168	N	0	1.195	0.939	-0.802	N
HETATM	169	C	0	2.207	0.035	-0.708	C
HETATM	170	O	0	3.415	0.219	-0.916	O
HETATM	171	N	0	1.730	-1.201	-0.196	N
HETATM	172	H	0	2.211	-2.028	-0.527	H
HETATM	173	C	0	0.278	-1.202	-0.234	C
HETATM	174	O	0	-0.187	-2.005	-1.286	O
HETATM	175	H	0	-1.027	-1.638	-1.737	H
HETATM	176	H	0	1.242	3.674	5.339	H
HETATM	177	H	0	2.954	3.969	5.154	H
HETATM	178	H	0	5.315	0.535	8.069	H
HETATM	179	H	0	3.720	0.692	8.765	H
HETATM	180	H	0	-5.562	3.304	4.886	H

HETATM	181	H	0	-6.199	2.057	5.932	H
HETATM	182	H	0	4.747	6.491	-6.347	H
HETATM	183	H	0	6.132	5.781	-5.554	H
HETATM	184	H	0	6.201	-0.460	-5.731	H
HETATM	185	H	0	6.642	-2.147	-5.620	H
HETATM	186	H	0	-4.157	-5.488	-7.244	H
HETATM	187	H	0	-4.641	-4.153	-6.226	H
HETATM	188	H	0	-1.038	-0.314	-6.779	H
HETATM	189	H	0	-2.766	-0.187	-6.548	H
HETATM	190	H	0	-6.961	2.995	1.439	H
HETATM	191	H	0	7.078	1.437	-2.693	H
HETATM	192	O	0	0.067	-0.943	2.210	O
HETATM	193	H	0	1.446	0.273	2.346	H
HETATM	194	H	0	-0.880	-0.480	2.129	H
HETATM	195	H	0	-4.577	8.719	0.848	H
HETATM	196	H	0	-6.150	8.075	0.442	H
HETATM	197	H	0	-3.232	-0.024	6.375	H

PROb

HETATM	1	C	0	-1.891	-0.657	-6.256	C
HETATM	2	H	0	-2.001	-1.691	-6.506	H
HETATM	3	C	0	-1.625	-0.469	-4.753	C
HETATM	4	H	0	-0.701	-0.984	-4.472	H
HETATM	5	H	0	-1.424	0.592	-4.554	H
HETATM	6	C	0	-2.712	-0.935	-3.833	C
HETATM	7	N	0	-2.402	-1.356	-2.553	N
HETATM	8	C	0	-3.554	-1.601	-1.953	C
HETATM	9	H	0	-3.674	-1.910	-0.929	H
HETATM	10	N	0	-4.595	-1.388	-2.778	N
HETATM	11	H	0	-5.553	-1.539	-2.455	H
HETATM	12	C	0	-4.081	-0.960	-3.987	C
HETATM	13	H	0	-4.714	-0.697	-4.819	H
HETATM	14	C	0	-3.870	-4.794	-6.428	C
HETATM	15	H	0	-2.977	-4.268	-6.692	H
HETATM	16	C	0	-3.677	-5.627	-5.137	C
HETATM	17	H	0	-4.507	-6.343	-5.057	H
HETATM	18	H	0	-2.763	-6.232	-5.206	H
HETATM	19	C	0	-3.650	-4.771	-3.856	C
HETATM	20	H	0	-2.784	-4.098	-3.859	H
HETATM	21	H	0	-4.529	-4.117	-3.837	H
HETATM	22	C	0	-3.658	-5.625	-2.572	C
HETATM	23	H	0	-4.511	-6.313	-2.605	H
HETATM	24	H	0	-2.763	-6.255	-2.516	H
HETATM	25	N	0	-3.773	-4.877	-1.320	N
HETATM	26	H	0	-4.694	-4.557	-0.984	H
HETATM	27	C	0	-2.747	-4.482	-0.553	C
HETATM	28	N	0	-1.459	-4.656	-0.930	N
HETATM	29	H	0	-1.288	-4.661	-1.925	H
HETATM	30	H	0	-0.797	-4.071	-0.405	H
HETATM	31	N	0	-2.994	-3.955	0.648	N
HETATM	32	H	0	-3.975	-3.776	0.890	H
HETATM	33	H	0	-2.224	-3.478	1.115	H
HETATM	34	C	0	5.821	-1.391	-5.579	C
HETATM	35	H	0	5.116	-1.625	-6.350	H
HETATM	36	C	0	5.137	-1.459	-4.194	C
HETATM	37	H	0	5.784	-1.971	-3.476	H
HETATM	38	H	0	5.018	-0.439	-3.808	H

HETATM	39	C	0	3.770	-2.083	-4.158	C
HETATM	40	N	0	2.880	-1.987	-5.213	N
HETATM	41	C	0	1.737	-2.446	-4.749	C
HETATM	42	H	0	0.814	-2.512	-5.308	H
HETATM	43	N	0	1.836	-2.874	-3.455	N
HETATM	44	H	0	1.063	-2.946	-2.800	H
HETATM	45	C	0	3.143	-2.638	-3.068	C
HETATM	46	H	0	3.516	-2.905	-2.090	H
HETATM	47	C	0	5.452	6.675	-5.554	C
HETATM	48	H	0	6.004	7.579	-5.709	H
HETATM	49	C	0	4.731	6.795	-4.189	C
HETATM	50	H	0	5.489	6.860	-3.399	H
HETATM	51	H	0	4.200	7.758	-4.172	H
HETATM	52	C	0	3.750	5.693	-3.845	C
HETATM	53	C	0	4.035	4.719	-2.880	C
HETATM	54	H	0	5.009	4.727	-2.391	H
HETATM	55	C	0	3.106	3.744	-2.511	C
HETATM	56	H	0	3.353	2.994	-1.764	H
HETATM	57	C	0	1.835	3.727	-3.107	C
HETATM	58	O	0	0.869	2.844	-2.789	O
HETATM	59	H	0	1.105	2.285	-2.002	H
HETATM	60	C	0	1.542	4.684	-4.095	C
HETATM	61	H	0	0.557	4.666	-4.551	H
HETATM	62	C	0	2.483	5.644	-4.449	C
HETATM	63	H	0	2.220	6.390	-5.197	H
HETATM	64	C	0	6.691	1.056	-1.790	C
HETATM	65	H	0	7.138	1.532	-0.942	H
HETATM	66	H	0	5.629	1.182	-1.750	H
HETATM	67	C	0	7.082	-0.396	-1.578	C
HETATM	68	O	0	8.144	-0.859	-2.010	O
HETATM	69	N	0	6.234	-1.054	-0.738	N
HETATM	70	H	0	5.290	-0.669	-0.677	H
HETATM	71	C	0	6.424	-2.430	-0.243	C
HETATM	72	H	0	6.264	-3.151	-1.052	H
HETATM	73	C	0	7.827	-2.699	0.352	C
HETATM	74	H	0	8.122	-1.822	0.952	H
HETATM	75	H	0	7.738	-3.541	1.047	H
HETATM	76	O	0	8.793	-3.064	-0.603	O
HETATM	77	H	0	8.768	-2.347	-1.270	H
HETATM	78	C	0	5.340	-2.682	0.830	C
HETATM	79	O	0	4.336	-3.358	0.474	O
HETATM	80	O	0	5.527	-2.194	1.992	O
HETATM	81	C	0	2.174	3.294	5.087	C
HETATM	82	H	0	2.385	2.489	5.759	H
HETATM	83	C	0	1.958	2.776	3.647	C
HETATM	84	H	0	0.950	2.354	3.608	H
HETATM	85	H	0	1.971	3.615	2.941	H
HETATM	86	C	0	2.887	1.708	3.161	C
HETATM	87	N	0	2.380	0.575	2.541	N
HETATM	88	C	0	3.375	-0.220	2.166	C
HETATM	89	H	0	3.266	-1.148	1.637	H
HETATM	90	N	0	4.523	0.344	2.531	N
HETATM	91	H	0	5.398	-0.152	2.360	H
HETATM	92	C	0	4.251	1.554	3.152	C
HETATM	93	H	0	5.032	2.200	3.517	H
HETATM	94	C	0	4.257	0.534	7.876	C
HETATM	95	H	0	3.994	1.240	7.115	H

HETATM	96	C	0	3.883	-0.894	7.400	C
HETATM	97	H	0	4.494	-1.631	7.938	H
HETATM	98	H	0	2.844	-1.109	7.684	H
HETATM	99	C	0	4.015	-1.118	5.883	C
HETATM	100	H	0	3.277	-0.496	5.360	H
HETATM	101	H	0	4.999	-0.786	5.529	H
HETATM	102	C	0	3.810	-2.597	5.496	C
HETATM	103	H	0	4.681	-3.178	5.818	H
HETATM	104	H	0	2.954	-3.012	6.039	H
HETATM	105	N	0	3.607	-2.859	4.072	N
HETATM	106	H	0	4.402	-2.773	3.415	H
HETATM	107	C	0	2.419	-3.148	3.510	C
HETATM	108	N	0	1.254	-3.007	4.211	N
HETATM	109	H	0	1.259	-2.259	4.891	H
HETATM	110	H	0	0.446	-2.922	3.592	H
HETATM	111	N	0	2.364	-3.629	2.278	N
HETATM	112	H	0	3.209	-3.635	1.651	H
HETATM	113	H	0	1.445	-3.636	1.829	H
HETATM	114	C	0	-5.334	2.471	5.428	C
HETATM	115	H	0	-4.564	2.677	6.142	H
HETATM	116	C	0	-4.739	1.412	4.445	C
HETATM	117	H	0	-5.258	0.454	4.554	H
HETATM	118	H	0	-4.918	1.719	3.414	H
HETATM	119	C	0	-3.263	1.227	4.627	C
HETATM	120	N	0	-2.738	0.485	5.674	N
HETATM	121	C	0	-1.376	0.570	5.610	C
HETATM	122	H	0	-0.731	0.072	6.322	H
HETATM	123	N	0	-0.989	1.317	4.599	N
HETATM	124	C	0	-2.155	1.731	3.982	C
HETATM	125	H	0	-2.124	2.366	3.106	H
HETATM	126	C	0	-5.305	8.661	0.070	C
HETATM	127	H	0	-5.608	9.624	-0.287	H
HETATM	128	C	0	-4.618	7.860	-1.066	C
HETATM	129	H	0	-5.370	7.478	-1.766	H
HETATM	130	H	0	-3.998	8.559	-1.646	H
HETATM	131	C	0	-3.739	6.715	-0.588	C
HETATM	132	C	0	-3.916	5.399	-1.035	C
HETATM	133	H	0	-4.733	5.178	-1.720	H
HETATM	134	C	0	-3.075	4.357	-0.639	C
HETATM	135	H	0	-3.238	3.341	-0.989	H
HETATM	136	C	0	-2.012	4.618	0.238	C
HETATM	137	O	0	-1.157	3.666	0.694	O
HETATM	138	H	0	-1.158	2.847	0.138	H
HETATM	139	C	0	-1.821	5.929	0.701	C
HETATM	140	H	0	-0.988	6.126	1.368	H
HETATM	141	C	0	-2.668	6.952	0.288	C
HETATM	142	H	0	-2.477	7.963	0.644	H
HETATM	143	C	0	-6.942	2.120	0.927	C
HETATM	144	H	0	-7.761	2.069	0.240	H
HETATM	145	H	0	-6.021	2.036	0.390	H
HETATM	146	C	0	-7.043	0.861	1.786	C
HETATM	147	O	0	-7.378	0.863	2.981	O
HETATM	148	N	0	-6.737	-0.244	1.067	N
HETATM	149	H	0	-6.576	-0.141	0.069	H
HETATM	150	C	0	-6.519	-1.630	1.480	C
HETATM	151	H	0	-5.611	-1.697	2.095	H
HETATM	152	C	0	-7.650	-2.243	2.325	C

HETATM	153	H	0	-8.609	-2.063	1.810	H
HETATM	154	H	0	-7.482	-3.324	2.357	H
HETATM	155	O	0	-7.660	-1.779	3.658	O
HETATM	156	H	0	-7.668	-0.799	3.602	H
HETATM	157	C	0	-6.251	-2.448	0.173	C
HETATM	158	O	0	-5.720	-3.589	0.340	O
HETATM	159	O	0	-6.569	-1.910	-0.913	O
HETATM	160	O	0	-0.285	-3.009	1.238	O
HETATM	161	C	0	0.026	-1.778	1.157	C
HETATM	162	N	0	-2.437	-0.290	1.163	N
HETATM	163	H	0	-3.367	-0.284	1.559	H
HETATM	164	C	0	-2.330	0.592	0.094	C
HETATM	165	O	0	-3.349	1.029	-0.444	O
HETATM	166	N	0	-1.069	1.022	-0.291	N
HETATM	167	C	0	0.057	0.368	-0.394	C
HETATM	168	N	0	1.203	1.008	-0.716	N
HETATM	169	C	0	2.243	0.117	-0.699	C
HETATM	170	O	0	3.442	0.360	-0.901	O
HETATM	171	N	0	1.796	-1.146	-0.298	N
HETATM	172	H	0	2.313	-1.959	-0.604	H
HETATM	173	C	0	0.344	-1.185	-0.249	C
HETATM	174	O	0	-0.161	-1.975	-1.291	O
HETATM	175	H	0	-0.994	-1.602	-1.740	H
HETATM	176	H	0	1.234	3.736	5.344	H
HETATM	177	H	0	2.946	4.031	5.159	H
HETATM	178	H	0	5.306	0.597	8.074	H
HETATM	179	H	0	3.712	0.754	8.770	H
HETATM	180	H	0	-5.571	3.366	4.891	H
HETATM	181	H	0	-6.207	2.119	5.937	H
HETATM	182	H	0	4.739	6.553	-6.343	H
HETATM	183	H	0	6.124	5.843	-5.549	H
HETATM	184	H	0	6.192	-0.398	-5.726	H
HETATM	185	H	0	6.634	-2.085	-5.615	H
HETATM	186	H	0	-4.166	-5.425	-7.240	H
HETATM	187	H	0	-4.650	-4.090	-6.222	H
HETATM	188	H	0	-1.047	-0.252	-6.774	H
HETATM	189	H	0	-2.774	-0.125	-6.543	H
HETATM	190	H	0	-6.969	3.057	1.444	H
HETATM	191	H	0	7.069	1.499	-2.688	H
HETATM	192	O	0	0.214	-1.035	2.161	O
HETATM	193	H	0	1.393	0.288	2.380	H
HETATM	194	H	0	-1.684	-0.285	1.843	H
HETATM	195	H	0	-4.586	8.781	0.853	H
HETATM	196	H	0	-6.158	8.137	0.447	H
HETATM	197	H	0	-3.277	-0.047	6.341	H

Pathway 3

1c

HETATM	1	C	0	29.217	30.257	33.034	C
HETATM	2	H	0	29.026	29.242	32.753	H
HETATM	3	C	0	29.701	30.468	34.474	C
HETATM	4	H	0	30.446	29.704	34.735	H
HETATM	5	H	0	30.239	31.425	34.529	H
HETATM	6	C	0	28.608	30.475	35.502	C
HETATM	7	N	0	28.885	30.622	36.845	N
HETATM	8	C	0	27.709	30.592	37.522	C
HETATM	9	H	0	27.652	30.641	38.602	H

HETATM	10	N	0	26.683	30.439	36.697	N
HETATM	11	C	0	27.237	30.374	35.434	C
HETATM	12	H	0	26.618	30.254	34.557	H
HETATM	13	C	0	27.013	26.543	32.754	C
HETATM	14	H	0	27.900	27.043	32.424	H
HETATM	15	C	0	27.048	25.869	34.131	C
HETATM	16	H	0	26.151	25.237	34.201	H
HETATM	17	H	0	27.899	25.177	34.193	H
HETATM	18	C	0	27.061	26.821	35.337	C
HETATM	19	H	0	28.061	27.246	35.484	H
HETATM	20	H	0	26.408	27.681	35.159	H
HETATM	21	C	0	26.586	26.109	36.621	C
HETATM	22	H	0	25.511	25.917	36.544	H
HETATM	23	H	0	27.065	25.123	36.706	H
HETATM	24	N	0	26.796	26.850	37.854	N
HETATM	25	H	0	26.012	27.407	38.279	H
HETATM	26	C	0	27.956	26.842	38.516	C
HETATM	27	N	0	29.018	26.132	38.049	N
HETATM	28	H	0	29.031	25.936	37.062	H
HETATM	29	H	0	29.928	26.367	38.463	H
HETATM	30	N	0	28.052	27.466	39.691	N
HETATM	31	H	0	27.333	28.185	39.978	H
HETATM	32	H	0	28.981	27.548	40.077	H
HETATM	33	C	0	37.354	29.581	33.459	C
HETATM	34	H	0	36.635	29.321	32.710	H
HETATM	35	C	0	37.096	28.860	34.797	C
HETATM	36	H	0	36.943	27.791	34.598	H
HETATM	37	H	0	37.993	28.945	35.416	H
HETATM	38	C	0	35.966	29.387	35.643	C
HETATM	39	N	0	35.033	30.327	35.243	N
HETATM	40	C	0	34.248	30.650	36.310	C
HETATM	41	H	0	33.447	31.375	36.259	H
HETATM	42	N	0	34.620	29.961	37.376	N
HETATM	43	C	0	35.675	29.171	36.968	C
HETATM	44	H	0	36.195	28.523	37.657	H
HETATM	45	C	0	37.258	37.692	33.598	C
HETATM	46	H	0	37.620	38.485	32.978	H
HETATM	47	C	0	35.758	37.916	33.892	C
HETATM	48	H	0	35.651	38.789	34.546	H
HETATM	49	H	0	35.269	38.193	32.947	H
HETATM	50	C	0	34.995	36.749	34.497	C
HETATM	51	C	0	34.392	36.833	35.759	C
HETATM	52	H	0	34.525	37.735	36.352	H
HETATM	53	C	0	33.598	35.806	36.276	C
HETATM	54	H	0	33.126	35.916	37.249	H
HETATM	55	C	0	33.385	34.644	35.521	C
HETATM	56	O	0	32.604	33.610	35.924	O
HETATM	57	H	0	32.198	33.759	36.810	H
HETATM	58	C	0	33.999	34.534	34.262	C
HETATM	59	H	0	33.819	33.639	33.674	H
HETATM	60	C	0	34.783	35.570	33.766	C
HETATM	61	H	0	35.221	35.468	32.774	H
HETATM	62	C	0	37.317	32.875	37.255	C
HETATM	63	H	0	37.323	33.683	37.956	H
HETATM	64	H	0	36.308	32.647	36.982	H
HETATM	65	C	0	38.089	31.662	37.756	C
HETATM	66	O	0	38.392	30.723	37.020	O

HETATM	67	N	0	38.427	31.695	39.072	N
HETATM	68	H	0	38.202	32.488	39.661	H
HETATM	69	C	0	38.899	30.539	39.812	C
HETATM	70	H	0	38.333	29.654	39.502	H
HETATM	71	C	0	40.393	30.204	39.594	C
HETATM	72	H	0	40.566	30.071	38.522	H
HETATM	73	H	0	41.000	31.058	39.940	H
HETATM	74	O	0	40.747	28.999	40.250	O
HETATM	75	H	0	40.234	28.975	41.077	H
HETATM	76	C	0	38.664	30.803	41.314	C
HETATM	77	O	0	38.576	31.999	41.679	O
HETATM	78	O	0	38.611	29.765	42.054	O
HETATM	79	C	0	33.808	34.737	45.813	C
HETATM	80	H	0	34.060	34.156	46.675	H
HETATM	81	C	0	33.303	33.987	44.569	C
HETATM	82	H	0	32.689	33.124	44.862	H
HETATM	83	H	0	32.624	34.641	44.011	H
HETATM	84	C	0	34.385	33.550	43.626	C
HETATM	85	N	0	34.108	33.141	42.334	N
HETATM	86	C	0	35.281	32.827	41.807	C
HETATM	87	H	0	35.427	32.432	40.812	H
HETATM	88	N	0	36.300	33.021	42.675	N
HETATM	89	C	0	35.743	33.483	43.845	C
HETATM	90	H	0	36.347	33.735	44.702	H
HETATM	91	C	0	35.807	32.077	48.556	C
HETATM	92	H	0	34.919	32.672	48.505	H
HETATM	93	C	0	35.873	30.784	47.730	C
HETATM	94	H	0	36.573	30.103	48.233	H
HETATM	95	H	0	34.902	30.270	47.744	H
HETATM	96	C	0	36.344	30.967	46.281	C
HETATM	97	H	0	35.589	31.499	45.693	H
HETATM	98	H	0	37.232	31.610	46.257	H
HETATM	99	C	0	36.692	29.615	45.618	C
HETATM	100	H	0	37.669	29.272	45.971	H
HETATM	101	H	0	35.966	28.852	45.927	H
HETATM	102	N	0	36.739	29.630	44.162	N
HETATM	103	H	0	37.622	29.745	43.639	H
HETATM	104	C	0	35.632	29.550	43.409	C
HETATM	105	N	0	34.418	29.577	43.991	N
HETATM	106	H	0	34.351	30.042	44.882	H
HETATM	107	H	0	33.582	29.693	43.405	H
HETATM	108	N	0	35.751	29.350	42.093	N
HETATM	109	H	0	36.706	29.282	41.743	H
HETATM	110	H	0	35.011	29.622	41.440	H
HETATM	111	C	0	25.766	33.864	45.918	C
HETATM	112	H	0	25.829	33.552	46.940	H
HETATM	113	C	0	25.645	32.643	44.983	C
HETATM	114	H	0	25.006	31.895	45.473	H
HETATM	115	H	0	25.123	32.902	44.059	H
HETATM	116	C	0	26.938	32.003	44.562	C
HETATM	117	N	0	28.140	32.130	45.244	N
HETATM	118	C	0	29.099	31.449	44.549	C
HETATM	119	H	0	30.136	31.415	44.849	H
HETATM	120	N	0	28.585	30.881	43.479	N
HETATM	121	C	0	27.245	31.215	43.475	C
HETATM	122	H	0	26.587	30.869	42.691	H
HETATM	123	C	0	26.062	40.185	40.587	C

HETATM	124	H	0	25.019	40.417	40.522	H
HETATM	125	C	0	26.338	39.014	39.619	C
HETATM	126	H	0	25.562	38.255	39.778	H
HETATM	127	H	0	26.169	39.380	38.595	H
HETATM	128	C	0	27.687	38.308	39.671	C
HETATM	129	C	0	27.769	36.961	39.283	C
HETATM	130	H	0	26.859	36.434	39.004	H
HETATM	131	C	0	28.979	36.271	39.231	C
HETATM	132	H	0	29.008	35.231	38.916	H
HETATM	133	C	0	30.171	36.921	39.580	C
HETATM	134	O	0	31.390	36.319	39.555	O
HETATM	135	H	0	31.335	35.378	39.289	H
HETATM	136	C	0	30.109	38.264	39.982	C
HETATM	137	H	0	31.032	38.765	40.259	H
HETATM	138	C	0	28.888	38.940	40.021	C
HETATM	139	H	0	28.880	39.982	40.331	H
HETATM	140	C	0	25.801	33.877	40.819	C
HETATM	141	H	0	25.761	34.015	39.759	H
HETATM	142	H	0	26.822	33.864	41.139	H
HETATM	143	C	0	25.043	32.562	41.046	C
HETATM	144	O	0	24.537	32.238	42.125	O
HETATM	145	N	0	24.946	31.816	39.923	N
HETATM	146	H	0	25.277	32.191	39.043	H
HETATM	147	C	0	24.244	30.556	39.760	C
HETATM	148	H	0	23.613	30.408	40.644	H
HETATM	149	C	0	23.373	30.666	38.491	C
HETATM	150	H	0	23.014	29.662	38.239	H
HETATM	151	H	0	22.510	31.311	38.699	H
HETATM	152	O	0	24.069	31.277	37.418	O
HETATM	153	H	0	24.869	30.753	37.175	H
HETATM	154	C	0	25.224	29.341	39.679	C
HETATM	155	O	0	24.830	28.354	38.997	O
HETATM	156	O	0	26.315	29.443	40.304	O
HETATM	157	O	0	32.205	30.243	42.522	O
HETATM	158	C	0	32.102	29.877	41.353	C
HETATM	159	N	0	31.805	28.597	40.993	N
HETATM	160	H	0	31.590	27.936	41.729	H
HETATM	161	C	0	31.478	28.151	39.676	C
HETATM	162	O	0	31.189	26.946	39.602	O
HETATM	163	N	0	31.411	29.027	38.648	N
HETATM	164	C	0	31.735	30.281	38.910	C
HETATM	165	N	0	31.434	31.344	38.175	N
HETATM	166	C	0	31.793	32.465	38.926	C
HETATM	167	O	0	31.646	33.643	38.541	O
HETATM	168	N	0	32.297	32.100	40.156	N
HETATM	169	H	0	32.762	32.719	40.828	H
HETATM	170	C	0	32.544	30.691	40.145	C
HETATM	171	O	0	33.922	30.345	39.996	O
HETATM	172	O	0	29.622	30.656	40.851	O
HETATM	173	H	0	29.770	31.608	40.785	H
HETATM	174	H	0	29.275	30.555	41.768	H
HETATM	175	H	0	34.151	30.211	39.018	H
HETATM	176	H	0	29.808	30.817	37.252	H
HETATM	177	H	0	34.964	30.742	34.327	H
HETATM	178	H	0	28.284	32.668	46.085	H
HETATM	179	H	0	26.788	25.795	31.983	H
HETATM	180	H	0	26.202	27.280	32.719	H

HETATM	181	H	0	29.980	30.623	32.337	H
HETATM	182	H	0	28.311	30.840	32.838	H
HETATM	183	H	0	37.417	36.750	33.062	H
HETATM	184	H	0	37.849	37.652	34.518	H
HETATM	185	H	0	37.807	33.224	36.340	H
HETATM	186	H	0	33.029	35.428	46.158	H
HETATM	187	H	0	34.674	35.358	45.562	H
HETATM	188	H	0	35.921	31.834	49.620	H
HETATM	189	H	0	36.649	32.733	48.305	H
HETATM	190	H	0	37.373	30.668	33.588	H
HETATM	191	H	0	38.334	29.287	33.072	H
HETATM	192	H	0	24.869	34.486	45.835	H
HETATM	193	H	0	26.624	34.499	45.672	H
HETATM	194	H	0	26.291	39.910	41.622	H
HETATM	195	H	0	26.640	41.080	40.337	H
HETATM	196	H	0	25.282	34.690	41.330	H
HETATM	197	H	0	37.273	32.727	42.493	H

TS1c

HETATM	1	C	0	29.217	30.257	33.034	C
HETATM	2	H	0	29.026	29.242	32.753	H
HETATM	3	C	0	29.663	30.479	34.483	C
HETATM	4	H	0	30.389	29.708	34.776	H
HETATM	5	H	0	30.212	31.428	34.544	H
HETATM	6	C	0	28.540	30.512	35.475	C
HETATM	7	N	0	28.781	30.703	36.818	N
HETATM	8	C	0	27.589	30.681	37.463	C
HETATM	9	H	0	27.496	30.757	38.538	H
HETATM	10	N	0	26.584	30.492	36.620	N
HETATM	11	C	0	27.171	30.395	35.373	C
HETATM	12	H	0	26.577	30.242	34.484	H
HETATM	13	C	0	27.013	26.543	32.754	C
HETATM	14	H	0	27.900	27.043	32.424	H
HETATM	15	C	0	27.065	25.877	34.135	C
HETATM	16	H	0	26.159	25.261	34.231	H
HETATM	17	H	0	27.906	25.172	34.182	H
HETATM	18	C	0	27.125	26.837	35.334	C
HETATM	19	H	0	28.133	27.253	35.448	H
HETATM	20	H	0	26.473	27.701	35.173	H
HETATM	21	C	0	26.686	26.135	36.636	C
HETATM	22	H	0	25.616	25.909	36.571	H
HETATM	23	H	0	27.196	25.167	36.733	H
HETATM	24	N	0	26.882	26.899	37.858	N
HETATM	25	H	0	26.089	27.427	38.287	H
HETATM	26	C	0	28.018	26.876	38.562	C
HETATM	27	N	0	29.104	26.190	38.121	N
HETATM	28	H	0	29.159	26.025	37.129	H
HETATM	29	H	0	29.990	26.456	38.579	H
HETATM	30	N	0	28.071	27.467	39.757	N
HETATM	31	H	0	27.366	28.192	40.029	H
HETATM	32	H	0	28.988	27.502	40.181	H
HETATM	33	C	0	37.354	29.581	33.459	C
HETATM	34	H	0	36.635	29.321	32.710	H
HETATM	35	C	0	37.053	28.914	34.820	C
HETATM	36	H	0	36.881	27.842	34.650	H
HETATM	37	H	0	37.944	28.996	35.448	H
HETATM	38	C	0	35.923	29.478	35.649	C

HETATM	39	N	0	34.987	30.404	35.226	N
HETATM	40	C	0	34.193	30.741	36.285	C
HETATM	41	H	0	33.388	31.459	36.210	H
HETATM	42	N	0	34.560	30.080	37.370	N
HETATM	43	C	0	35.623	29.291	36.980	C
HETATM	44	H	0	36.142	28.655	37.682	H
HETATM	45	C	0	37.258	37.692	33.598	C
HETATM	46	H	0	37.620	38.485	32.978	H
HETATM	47	C	0	35.759	37.931	33.890	C
HETATM	48	H	0	35.663	38.784	34.572	H
HETATM	49	H	0	35.284	38.249	32.951	H
HETATM	50	C	0	34.966	36.761	34.446	C
HETATM	51	C	0	34.377	36.802	35.715	C
HETATM	52	H	0	34.546	37.667	36.352	H
HETATM	53	C	0	33.552	35.777	36.187	C
HETATM	54	H	0	33.095	35.851	37.170	H
HETATM	55	C	0	33.291	34.663	35.375	C
HETATM	56	O	0	32.473	33.642	35.735	O
HETATM	57	H	0	32.076	33.776	36.630	H
HETATM	58	C	0	33.894	34.595	34.109	C
HETATM	59	H	0	33.680	33.736	33.479	H
HETATM	60	C	0	34.711	35.627	33.659	C
HETATM	61	H	0	35.140	35.561	32.660	H
HETATM	62	C	0	37.317	32.875	37.255	C
HETATM	63	H	0	37.323	33.683	37.956	H
HETATM	64	H	0	36.308	32.647	36.982	H
HETATM	65	C	0	38.083	31.657	37.755	C
HETATM	66	O	0	38.420	30.740	37.006	O
HETATM	67	N	0	38.366	31.656	39.084	N
HETATM	68	H	0	38.133	32.442	39.678	H
HETATM	69	C	0	38.789	30.479	39.822	C
HETATM	70	H	0	38.200	29.614	39.497	H
HETATM	71	C	0	40.275	30.097	39.634	C
HETATM	72	H	0	40.468	29.964	38.566	H
HETATM	73	H	0	40.902	30.929	39.999	H
HETATM	74	O	0	40.573	28.878	40.292	O
HETATM	75	H	0	40.032	28.864	41.102	H
HETATM	76	C	0	38.532	30.741	41.323	C
HETATM	77	O	0	38.487	31.940	41.691	O
HETATM	78	O	0	38.424	29.704	42.054	O
HETATM	79	C	0	33.808	34.737	45.813	C
HETATM	80	H	0	34.060	34.156	46.675	H
HETATM	81	C	0	33.274	33.998	44.572	C
HETATM	82	H	0	32.699	33.109	44.871	H
HETATM	83	H	0	32.559	34.644	44.050	H
HETATM	84	C	0	34.332	33.607	43.583	C
HETATM	85	N	0	34.044	33.350	42.255	N
HETATM	86	C	0	35.199	33.006	41.711	C
HETATM	87	H	0	35.334	32.694	40.685	H
HETATM	88	N	0	36.219	33.043	42.600	N
HETATM	89	C	0	35.679	33.426	43.804	C
HETATM	90	H	0	36.287	33.548	44.686	H
HETATM	91	C	0	35.807	32.077	48.556	C
HETATM	92	H	0	34.919	32.672	48.505	H
HETATM	93	C	0	35.826	30.758	47.770	C
HETATM	94	H	0	36.577	30.103	48.232	H
HETATM	95	H	0	34.868	30.233	47.884	H

HETATM	96	C	0	36.165	30.901	46.281	C
HETATM	97	H	0	35.367	31.433	45.752	H
HETATM	98	H	0	37.060	31.525	46.162	H
HETATM	99	C	0	36.428	29.535	45.613	C
HETATM	100	H	0	37.381	29.132	45.970	H
HETATM	101	H	0	35.659	28.814	45.921	H
HETATM	102	N	0	36.482	29.553	44.160	N
HETATM	103	H	0	37.373	29.656	43.655	H
HETATM	104	C	0	35.377	29.555	43.389	C
HETATM	105	N	0	34.152	29.610	43.932	N
HETATM	106	H	0	34.098	29.970	44.871	H
HETATM	107	H	0	33.329	29.887	43.324	H
HETATM	108	N	0	35.517	29.418	42.068	N
HETATM	109	H	0	36.471	29.349	41.721	H
HETATM	110	H	0	34.765	29.703	41.435	H
HETATM	111	C	0	25.766	33.864	45.918	C
HETATM	112	H	0	25.829	33.552	46.940	H
HETATM	113	C	0	25.778	32.639	44.975	C
HETATM	114	H	0	25.256	31.810	45.472	H
HETATM	115	H	0	25.204	32.833	44.064	H
HETATM	116	C	0	27.118	32.156	44.494	C
HETATM	117	N	0	28.332	32.389	45.133	N
HETATM	118	C	0	29.337	31.850	44.402	C
HETATM	119	H	0	30.391	31.910	44.624	H
HETATM	120	N	0	28.818	31.265	43.336	N
HETATM	121	C	0	27.450	31.439	43.367	C
HETATM	122	H	0	26.812	31.042	42.590	H
HETATM	123	C	0	26.062	40.185	40.587	C
HETATM	124	H	0	25.019	40.417	40.522	H
HETATM	125	C	0	26.269	38.878	39.795	C
HETATM	126	H	0	25.546	38.142	40.174	H
HETATM	127	H	0	25.958	39.065	38.757	H
HETATM	128	C	0	27.641	38.216	39.772	C
HETATM	129	C	0	27.770	36.966	39.145	C
HETATM	130	H	0	26.891	36.503	38.699	H
HETATM	131	C	0	28.988	36.294	39.066	C
HETATM	132	H	0	29.058	35.331	38.566	H
HETATM	133	C	0	30.141	36.866	39.626	C
HETATM	134	O	0	31.361	36.272	39.590	O
HETATM	135	H	0	31.329	35.378	39.181	H
HETATM	136	C	0	30.031	38.113	40.256	C
HETATM	137	H	0	30.923	38.555	40.690	H
HETATM	138	C	0	28.800	38.773	40.324	C
HETATM	139	H	0	28.753	39.739	40.820	H
HETATM	140	C	0	25.801	33.877	40.819	C
HETATM	141	H	0	25.761	34.015	39.759	H
HETATM	142	H	0	26.822	33.864	41.139	H
HETATM	143	C	0	25.071	32.551	41.061	C
HETATM	144	O	0	24.686	32.172	42.175	O
HETATM	145	N	0	24.871	31.848	39.929	N
HETATM	146	H	0	25.114	32.246	39.030	H
HETATM	147	C	0	24.235	30.550	39.797	C
HETATM	148	H	0	23.608	30.383	40.681	H
HETATM	149	C	0	23.362	30.597	38.523	C
HETATM	150	H	0	23.090	29.568	38.265	H
HETATM	151	H	0	22.448	31.168	38.731	H
HETATM	152	O	0	24.010	31.271	37.458	O

HETATM	153	H	0	24.815	30.780	37.159	H
HETATM	154	C	0	25.273	29.381	39.746	C
HETATM	155	O	0	24.910	28.353	39.113	O
HETATM	156	O	0	26.370	29.552	40.349	O
HETATM	157	O	0	32.078	30.561	42.645	O
HETATM	158	C	0	31.687	30.268	41.465	C
HETATM	159	N	0	31.574	28.871	41.153	N
HETATM	160	H	0	31.481	28.236	41.934	H
HETATM	161	C	0	31.290	28.349	39.904	C
HETATM	162	O	0	31.063	27.122	39.835	O
HETATM	163	N	0	31.184	29.177	38.809	N
HETATM	164	C	0	31.543	30.427	38.979	C
HETATM	165	N	0	31.277	31.449	38.149	N
HETATM	166	C	0	31.656	32.606	38.801	C
HETATM	167	O	0	31.555	33.761	38.325	O
HETATM	168	N	0	32.116	32.340	40.082	N
HETATM	169	H	0	32.649	32.989	40.667	H
HETATM	170	C	0	32.324	30.915	40.203	C
HETATM	171	O	0	33.684	30.526	40.121	O
HETATM	172	O	0	30.127	30.833	41.270	O
HETATM	173	H	0	30.283	31.760	41.015	H
HETATM	174	H	0	29.476	30.934	42.375	H
HETATM	175	H	0	33.944	30.382	39.170	H
HETATM	176	H	0	29.699	30.919	37.238	H
HETATM	177	H	0	34.921	30.803	34.302	H
HETATM	178	H	0	28.451	32.911	45.988	H
HETATM	179	H	0	26.786	25.791	31.988	H
HETATM	180	H	0	26.201	27.279	32.722	H
HETATM	181	H	0	30.001	30.612	32.355	H
HETATM	182	H	0	28.321	30.845	32.808	H
HETATM	183	H	0	37.408	36.751	33.058	H
HETATM	184	H	0	37.850	37.645	34.517	H
HETATM	185	H	0	37.809	33.223	36.340	H
HETATM	186	H	0	33.050	35.447	46.168	H
HETATM	187	H	0	34.684	35.337	45.549	H
HETATM	188	H	0	35.953	31.870	49.623	H
HETATM	189	H	0	36.644	32.716	48.251	H
HETATM	190	H	0	37.407	30.671	33.552	H
HETATM	191	H	0	38.329	29.239	33.099	H
HETATM	192	H	0	24.819	34.399	45.809	H
HETATM	193	H	0	26.566	34.578	45.697	H
HETATM	194	H	0	26.332	40.071	41.642	H
HETATM	195	H	0	26.640	41.018	40.174	H
HETATM	196	H	0	25.281	34.689	41.332	H
HETATM	197	H	0	37.174	32.695	42.415	H

INT1c

HETATM	1	C	0	-2.402	-1.427	-6.532	C
HETATM	2	H	0	-2.593	-2.441	-6.813	H
HETATM	3	C	0	-1.959	-1.204	-5.083	C
HETATM	4	H	0	-1.229	-1.970	-4.788	H
HETATM	5	H	0	-1.413	-0.252	-5.021	H
HETATM	6	C	0	-3.081	-1.176	-4.090	C
HETATM	7	N	0	-2.835	-0.985	-2.748	N
HETATM	8	C	0	-4.024	-1.016	-2.099	C
HETATM	9	H	0	-4.111	-0.940	-1.023	H
HETATM	10	N	0	-5.031	-1.211	-2.937	N

HETATM	11	C	0	-4.449	-1.303	-4.187	C
HETATM	12	H	0	-5.046	-1.459	-5.074	H
HETATM	13	C	0	-4.606	-5.141	-6.812	C
HETATM	14	H	0	-3.719	-4.641	-7.141	H
HETATM	15	C	0	-4.551	-5.808	-5.431	C
HETATM	16	H	0	-5.457	-6.423	-5.332	H
HETATM	17	H	0	-3.711	-6.514	-5.387	H
HETATM	18	C	0	-4.486	-4.848	-4.232	C
HETATM	19	H	0	-3.479	-4.426	-4.128	H
HETATM	20	H	0	-5.145	-3.989	-4.387	H
HETATM	21	C	0	-4.907	-5.553	-2.926	C
HETATM	22	H	0	-5.976	-5.783	-2.977	H
HETATM	23	H	0	-4.392	-6.519	-2.836	H
HETATM	24	N	0	-4.698	-4.789	-1.706	N
HETATM	25	H	0	-5.486	-4.268	-1.264	H
HETATM	26	C	0	-3.551	-4.806	-1.018	C
HETATM	27	N	0	-2.469	-5.483	-1.475	N
HETATM	28	H	0	-2.430	-5.651	-2.467	H
HETATM	29	H	0	-1.574	-5.218	-1.029	H
HETATM	30	N	0	-3.486	-4.218	0.179	N
HETATM	31	H	0	-4.193	-3.509	0.472	H
HETATM	32	H	0	-2.563	-4.178	0.592	H
HETATM	33	C	0	5.735	-2.103	-6.107	C
HETATM	34	H	0	5.016	-2.363	-6.855	H
HETATM	35	C	0	5.433	-2.767	-4.744	C
HETATM	36	H	0	5.258	-3.839	-4.913	H
HETATM	37	H	0	6.324	-2.686	-4.116	H
HETATM	38	C	0	4.305	-2.198	-3.917	C
HETATM	39	N	0	3.361	-1.282	-4.347	N
HETATM	40	C	0	2.570	-0.938	-3.287	C
HETATM	41	H	0	1.759	-0.227	-3.367	H
HETATM	42	N	0	2.946	-1.584	-2.196	N
HETATM	43	C	0	4.012	-2.371	-2.583	C
HETATM	44	H	0	4.538	-2.996	-1.875	H
HETATM	45	C	0	5.639	6.008	-5.968	C
HETATM	46	H	0	6.001	6.802	-6.588	H
HETATM	47	C	0	4.142	6.252	-5.671	C
HETATM	48	H	0	4.051	7.099	-4.981	H
HETATM	49	H	0	3.668	6.582	-6.606	H
HETATM	50	C	0	3.343	5.081	-5.126	C
HETATM	51	C	0	2.753	5.113	-3.857	C
HETATM	52	H	0	2.927	5.971	-3.211	H
HETATM	53	C	0	1.923	4.088	-3.395	C
HETATM	54	H	0	1.466	4.155	-2.412	H
HETATM	55	C	0	1.656	2.984	-4.217	C
HETATM	56	O	0	0.832	1.965	-3.868	O
HETATM	57	H	0	0.433	2.095	-2.972	H
HETATM	58	C	0	2.260	2.924	-5.484	C
HETATM	59	H	0	2.042	2.072	-6.122	H
HETATM	60	C	0	3.082	3.956	-5.924	C
HETATM	61	H	0	3.512	3.896	-6.923	H
HETATM	62	C	0	5.698	1.191	-2.311	C
HETATM	63	H	0	5.705	2.000	-1.610	H
HETATM	64	H	0	4.689	0.964	-2.583	H
HETATM	65	C	0	6.462	-0.029	-1.812	C
HETATM	66	O	0	6.813	-0.936	-2.567	O
HETATM	67	N	0	6.723	-0.045	-0.479	N

HETATM	68	H	0	6.480	0.734	0.120	H
HETATM	69	C	0	7.130	-1.229	0.257	C
HETATM	70	H	0	6.540	-2.089	-0.079	H
HETATM	71	C	0	8.616	-1.619	0.082	C
HETATM	72	H	0	8.820	-1.748	-0.984	H
HETATM	73	H	0	9.244	-0.793	0.458	H
HETATM	74	O	0	8.900	-2.843	0.738	O
HETATM	75	H	0	8.349	-2.857	1.542	H
HETATM	76	C	0	6.860	-0.974	1.756	C
HETATM	77	O	0	6.814	0.224	2.129	O
HETATM	78	O	0	6.744	-2.013	2.483	O
HETATM	79	C	0	2.189	3.053	6.247	C
HETATM	80	H	0	2.441	2.472	7.110	H
HETATM	81	C	0	1.632	2.318	5.015	C
HETATM	82	H	0	1.057	1.432	5.324	H
HETATM	83	H	0	0.913	2.968	4.502	H
HETATM	84	C	0	2.676	1.923	4.013	C
HETATM	85	N	0	2.374	1.678	2.687	N
HETATM	86	C	0	3.521	1.324	2.131	C
HETATM	87	H	0	3.644	1.016	1.103	H
HETATM	88	N	0	4.547	1.343	3.013	N
HETATM	89	C	0	4.022	1.724	4.223	C
HETATM	90	H	0	4.638	1.830	5.102	H
HETATM	91	C	0	4.188	0.393	8.990	C
HETATM	92	H	0	3.300	0.988	8.940	H
HETATM	93	C	0	4.188	-0.929	8.212	C
HETATM	94	H	0	4.955	-1.580	8.653	H
HETATM	95	H	0	3.235	-1.456	8.359	H
HETATM	96	C	0	4.478	-0.792	6.712	C
HETATM	97	H	0	3.671	-0.246	6.211	H
HETATM	98	H	0	5.380	-0.184	6.563	H
HETATM	99	C	0	4.693	-2.161	6.036	C
HETATM	100	H	0	5.627	-2.602	6.399	H
HETATM	101	H	0	3.893	-2.853	6.333	H
HETATM	102	N	0	4.760	-2.137	4.584	N
HETATM	103	H	0	5.659	-2.055	4.091	H
HETATM	104	C	0	3.664	-2.118	3.796	C
HETATM	105	N	0	2.433	-2.017	4.311	N
HETATM	106	H	0	2.377	-1.651	5.248	H
HETATM	107	H	0	1.604	-1.730	3.684	H
HETATM	108	N	0	3.827	-2.285	2.480	N
HETATM	109	H	0	4.784	-2.373	2.150	H
HETATM	110	H	0	3.090	-1.986	1.834	H
HETATM	111	C	0	-5.853	2.180	6.352	C
HETATM	112	H	0	-5.790	1.869	7.374	H
HETATM	113	C	0	-5.862	0.957	5.408	C
HETATM	114	H	0	-6.391	0.133	5.905	H
HETATM	115	H	0	-6.438	1.159	4.501	H
HETATM	116	C	0	-4.535	0.450	4.910	C
HETATM	117	N	0	-3.299	0.707	5.500	N
HETATM	118	C	0	-2.314	0.148	4.766	C
HETATM	119	H	0	-1.249	0.211	4.930	H
HETATM	120	N	0	-2.879	-0.477	3.745	N
HETATM	121	C	0	-4.247	-0.309	3.800	C
HETATM	122	H	0	-4.899	-0.730	3.047	H
HETATM	123	C	0	-5.557	8.501	1.021	C
HETATM	124	H	0	-6.599	8.733	0.957	H

HETATM	125	C	0	-5.359	7.181	0.250	C
HETATM	126	H	0	-6.077	6.452	0.651	H
HETATM	127	H	0	-5.685	7.351	-0.787	H
HETATM	128	C	0	-3.988	6.518	0.216	C
HETATM	129	C	0	-3.860	5.278	-0.433	C
HETATM	130	H	0	-4.739	4.829	-0.892	H
HETATM	131	C	0	-2.646	4.603	-0.519	C
HETATM	132	H	0	-2.577	3.649	-1.036	H
HETATM	133	C	0	-1.492	5.159	0.058	C
HETATM	134	O	0	-0.276	4.561	0.017	O
HETATM	135	H	0	-0.308	3.675	-0.411	H
HETATM	136	C	0	-1.601	6.396	0.709	C
HETATM	137	H	0	-0.709	6.826	1.154	H
HETATM	138	C	0	-2.829	7.059	0.783	C
HETATM	139	H	0	-2.874	8.017	1.295	H
HETATM	140	C	0	-5.818	2.193	1.253	C
HETATM	141	H	0	-5.858	2.332	0.193	H
HETATM	142	H	0	-4.797	2.180	1.573	H
HETATM	143	C	0	-6.542	0.867	1.503	C
HETATM	144	O	0	-6.930	0.498	2.621	O
HETATM	145	N	0	-6.729	0.148	0.381	N
HETATM	146	H	0	-6.493	0.538	-0.523	H
HETATM	147	C	0	-7.354	-1.157	0.266	C
HETATM	148	H	0	-7.980	-1.317	1.151	H
HETATM	149	C	0	-8.228	-1.131	-1.009	C
HETATM	150	H	0	-8.482	-2.166	-1.261	H
HETATM	151	H	0	-9.152	-0.576	-0.802	H
HETATM	152	O	0	-7.594	-0.450	-2.077	O
HETATM	153	H	0	-6.786	-0.935	-2.385	H
HETATM	154	C	0	-6.305	-2.318	0.233	C
HETATM	155	O	0	-6.652	-3.350	-0.401	O
HETATM	156	O	0	-5.217	-2.137	0.850	O
HETATM	157	O	0	0.356	-1.119	3.052	O
HETATM	158	C	0	-0.029	-1.397	1.844	C
HETATM	159	N	0	-0.059	-2.817	1.526	N
HETATM	160	H	0	-0.121	-3.456	2.306	H
HETATM	161	C	0	-0.295	-3.341	0.275	C
HETATM	162	O	0	-0.482	-4.576	0.186	O
HETATM	163	N	0	-0.404	-2.507	-0.819	N
HETATM	164	C	0	-0.068	-1.254	-0.638	C
HETATM	165	N	0	-0.338	-0.231	-1.470	N
HETATM	166	C	0	0.012	0.928	-0.809	C
HETATM	167	O	0	-0.097	2.084	-1.286	O
HETATM	168	N	0	0.454	0.668	0.478	N
HETATM	169	H	0	0.972	1.323	1.069	H
HETATM	170	C	0	0.675	-0.756	0.607	C
HETATM	171	O	0	2.040	-1.127	0.556	O
HETATM	172	O	0	-1.484	-0.921	1.629	O
HETATM	173	H	0	-1.373	0.019	1.399	H
HETATM	174	H	0	-2.298	-0.842	2.898	H
HETATM	175	H	0	2.317	-1.273	-0.388	H
HETATM	176	H	0	-1.914	-0.760	-2.337	H
HETATM	177	H	0	3.289	-0.895	-5.274	H
HETATM	178	H	0	-3.155	1.261	6.330	H
HETATM	179	H	0	-4.834	-5.892	-7.578	H
HETATM	180	H	0	-5.417	-4.404	-6.841	H
HETATM	181	H	0	-1.617	-1.072	-7.210	H

HETATM	182	H	0	-3.297	-0.839	-6.760	H
HETATM	183	H	0	5.785	5.068	-6.510	H
HETATM	184	H	0	6.234	5.958	-5.051	H
HETATM	185	H	0	6.191	1.539	-3.225	H
HETATM	186	H	0	1.447	3.777	6.608	H
HETATM	187	H	0	3.072	3.639	5.972	H
HETATM	188	H	0	4.344	0.194	10.058	H
HETATM	189	H	0	5.024	1.027	8.670	H
HETATM	190	H	0	5.790	-1.013	-6.015	H
HETATM	191	H	0	6.710	-2.446	-6.466	H
HETATM	192	H	0	-6.793	2.727	6.245	H
HETATM	193	H	0	-5.046	2.887	6.129	H
HETATM	194	H	0	-5.282	8.405	2.077	H
HETATM	195	H	0	-4.978	9.325	0.591	H
HETATM	196	H	0	-6.337	3.006	1.766	H
HETATM	197	H	0	5.498	0.982	2.823	H

TS2c

HETATM	1	C	0	-2.402	-1.427	-6.532	C
HETATM	2	H	0	-2.593	-2.441	-6.813	H
HETATM	3	C	0	-1.973	-1.200	-5.078	C
HETATM	4	H	0	-1.263	-1.976	-4.761	H
HETATM	5	H	0	-1.412	-0.258	-5.016	H
HETATM	6	C	0	-3.106	-1.137	-4.100	C
HETATM	7	N	0	-2.880	-0.822	-2.781	N
HETATM	8	C	0	-4.067	-0.871	-2.133	C
HETATM	9	H	0	-4.163	-0.716	-1.067	H
HETATM	10	N	0	-5.059	-1.190	-2.953	N
HETATM	11	C	0	-4.465	-1.348	-4.190	C
HETATM	12	H	0	-5.047	-1.604	-5.064	H
HETATM	13	C	0	-4.606	-5.141	-6.812	C
HETATM	14	H	0	-3.719	-4.641	-7.141	H
HETATM	15	C	0	-4.543	-5.852	-5.454	C
HETATM	16	H	0	-5.448	-6.472	-5.371	H
HETATM	17	H	0	-3.701	-6.558	-5.437	H
HETATM	18	C	0	-4.473	-4.933	-4.224	C
HETATM	19	H	0	-3.466	-4.514	-4.110	H
HETATM	20	H	0	-5.133	-4.069	-4.349	H
HETATM	21	C	0	-4.890	-5.675	-2.936	C
HETATM	22	H	0	-5.961	-5.895	-2.983	H
HETATM	23	H	0	-4.385	-6.649	-2.878	H
HETATM	24	N	0	-4.660	-4.945	-1.701	N
HETATM	25	H	0	-5.416	-4.372	-1.271	H
HETATM	26	C	0	-3.482	-4.919	-1.062	C
HETATM	27	N	0	-2.424	-5.619	-1.540	N
HETATM	28	H	0	-2.449	-5.855	-2.518	H
HETATM	29	H	0	-1.474	-5.375	-1.209	H
HETATM	30	N	0	-3.382	-4.270	0.100	N
HETATM	31	H	0	-4.121	-3.601	0.388	H
HETATM	32	H	0	-2.441	-4.055	0.443	H
HETATM	33	C	0	5.735	-2.103	-6.107	C
HETATM	34	H	0	5.016	-2.363	-6.855	H
HETATM	35	C	0	5.457	-2.793	-4.754	C
HETATM	36	H	0	5.292	-3.864	-4.937	H
HETATM	37	H	0	6.355	-2.712	-4.136	H
HETATM	38	C	0	4.332	-2.252	-3.905	C
HETATM	39	N	0	3.375	-1.335	-4.305	N

HETATM	40	C	0	2.590	-1.025	-3.231	C
HETATM	41	H	0	1.763	-0.328	-3.283	H
HETATM	42	N	0	2.985	-1.692	-2.160	N
HETATM	43	C	0	4.055	-2.459	-2.573	C
HETATM	44	H	0	4.588	-3.100	-1.887	H
HETATM	45	C	0	5.639	6.008	-5.968	C
HETATM	46	H	0	6.001	6.802	-6.588	H
HETATM	47	C	0	4.172	6.299	-5.581	C
HETATM	48	H	0	4.151	7.125	-4.859	H
HETATM	49	H	0	3.660	6.678	-6.477	H
HETATM	50	C	0	3.364	5.137	-5.030	C
HETATM	51	C	0	2.811	5.160	-3.744	C
HETATM	52	H	0	3.020	6.005	-3.091	H
HETATM	53	C	0	1.976	4.143	-3.274	C
HETATM	54	H	0	1.550	4.196	-2.276	H
HETATM	55	C	0	1.665	3.056	-4.105	C
HETATM	56	O	0	0.837	2.047	-3.749	O
HETATM	57	H	0	0.441	2.181	-2.851	H
HETATM	58	C	0	2.232	3.006	-5.390	C
HETATM	59	H	0	1.982	2.166	-6.033	H
HETATM	60	C	0	3.060	4.029	-5.837	C
HETATM	61	H	0	3.460	3.978	-6.848	H
HETATM	62	C	0	5.698	1.191	-2.311	C
HETATM	63	H	0	5.705	2.000	-1.610	H
HETATM	64	H	0	4.689	0.964	-2.583	H
HETATM	65	C	0	6.470	-0.024	-1.815	C
HETATM	66	O	0	6.836	-0.925	-2.570	O
HETATM	67	N	0	6.731	-0.039	-0.481	N
HETATM	68	H	0	6.456	0.728	0.121	H
HETATM	69	C	0	7.166	-1.217	0.246	C
HETATM	70	H	0	6.610	-2.092	-0.108	H
HETATM	71	C	0	8.667	-1.554	0.084	C
HETATM	72	H	0	8.884	-1.661	-0.982	H
HETATM	73	H	0	9.262	-0.712	0.477	H
HETATM	74	O	0	8.988	-2.776	0.725	O
HETATM	75	H	0	8.438	-2.819	1.527	H
HETATM	76	C	0	6.867	-0.991	1.744	C
HETATM	77	O	0	6.750	0.197	2.132	O
HETATM	78	O	0	6.796	-2.042	2.461	O
HETATM	79	C	0	2.189	3.053	6.247	C
HETATM	80	H	0	2.441	2.472	7.110	H
HETATM	81	C	0	1.583	2.307	5.050	C
HETATM	82	H	0	0.968	1.464	5.405	H
HETATM	83	H	0	0.890	2.972	4.521	H
HETATM	84	C	0	2.597	1.824	4.057	C
HETATM	85	N	0	2.237	1.407	2.791	N
HETATM	86	C	0	3.370	1.053	2.206	C
HETATM	87	H	0	3.449	0.642	1.209	H
HETATM	88	N	0	4.440	1.228	3.016	N
HETATM	89	C	0	3.962	1.721	4.205	C
HETATM	90	H	0	4.620	1.959	5.026	H
HETATM	91	C	0	4.188	0.393	8.990	C
HETATM	92	H	0	3.300	0.988	8.940	H
HETATM	93	C	0	4.209	-0.906	8.170	C
HETATM	94	H	0	4.972	-1.565	8.606	H
HETATM	95	H	0	3.256	-1.441	8.285	H
HETATM	96	C	0	4.525	-0.730	6.677	C

HETATM	97	H	0	3.724	-0.172	6.178	H
HETATM	98	H	0	5.425	-0.115	6.557	H
HETATM	99	C	0	4.754	-2.086	5.978	C
HETATM	100	H	0	5.685	-2.528	6.346	H
HETATM	101	H	0	3.951	-2.783	6.252	H
HETATM	102	N	0	4.842	-2.047	4.523	N
HETATM	103	H	0	5.748	-2.003	4.032	H
HETATM	104	C	0	3.763	-2.103	3.724	C
HETATM	105	N	0	2.524	-1.981	4.238	N
HETATM	106	H	0	2.446	-1.449	5.090	H
HETATM	107	H	0	1.743	-1.827	3.582	H
HETATM	108	N	0	3.914	-2.355	2.425	N
HETATM	109	H	0	4.866	-2.493	2.092	H
HETATM	110	H	0	3.156	-2.142	1.767	H
HETATM	111	C	0	-5.853	2.180	6.352	C
HETATM	112	H	0	-5.790	1.869	7.374	H
HETATM	113	C	0	-5.860	0.969	5.394	C
HETATM	114	H	0	-6.351	0.123	5.891	H
HETATM	115	H	0	-6.465	1.168	4.503	H
HETATM	116	C	0	-4.535	0.509	4.852	C
HETATM	117	N	0	-3.294	0.856	5.376	N
HETATM	118	C	0	-2.309	0.331	4.622	C
HETATM	119	H	0	-1.247	0.461	4.759	H
HETATM	120	N	0	-2.881	-0.356	3.645	N
HETATM	121	C	0	-4.252	-0.268	3.752	C
HETATM	122	H	0	-4.903	-0.747	3.033	H
HETATM	123	C	0	-5.557	8.501	1.021	C
HETATM	124	H	0	-6.599	8.733	0.957	H
HETATM	125	C	0	-5.409	7.005	0.672	C
HETATM	126	H	0	-5.980	6.435	1.422	H
HETATM	127	H	0	-5.937	6.828	-0.274	H
HETATM	128	C	0	-4.020	6.394	0.548	C
HETATM	129	C	0	-3.850	5.243	-0.239	C
HETATM	130	H	0	-4.700	4.852	-0.796	H
HETATM	131	C	0	-2.630	4.575	-0.334	C
HETATM	132	H	0	-2.528	3.688	-0.954	H
HETATM	133	C	0	-1.511	5.054	0.367	C
HETATM	134	O	0	-0.297	4.459	0.331	O
HETATM	135	H	0	-0.301	3.616	-0.185	H
HETATM	136	C	0	-1.659	6.215	1.143	C
HETATM	137	H	0	-0.791	6.593	1.675	H
HETATM	138	C	0	-2.891	6.865	1.231	C
HETATM	139	H	0	-2.966	7.758	1.848	H
HETATM	140	C	0	-5.818	2.193	1.253	C
HETATM	141	H	0	-5.858	2.332	0.193	H
HETATM	142	H	0	-4.797	2.180	1.573	H
HETATM	143	C	0	-6.526	0.860	1.508	C
HETATM	144	O	0	-6.919	0.498	2.628	O
HETATM	145	N	0	-6.679	0.116	0.398	N
HETATM	146	H	0	-6.467	0.508	-0.512	H
HETATM	147	C	0	-7.273	-1.206	0.301	C
HETATM	148	H	0	-7.873	-1.375	1.202	H
HETATM	149	C	0	-8.178	-1.209	-0.952	C
HETATM	150	H	0	-8.399	-2.252	-1.201	H
HETATM	151	H	0	-9.117	-0.691	-0.718	H
HETATM	152	O	0	-7.600	-0.501	-2.033	O
HETATM	153	H	0	-6.789	-0.960	-2.374	H

HETATM	154	C	0	-6.192	-2.340	0.257	C
HETATM	155	O	0	-6.522	-3.380	-0.373	O
HETATM	156	O	0	-5.106	-2.139	0.871	O
HETATM	157	O	0	0.273	-1.327	2.831	O
HETATM	158	C	0	-0.156	-1.291	1.685	C
HETATM	159	N	0	-0.620	-3.355	0.840	N
HETATM	160	H	0	-0.377	-4.178	1.391	H
HETATM	161	C	0	-0.213	-3.555	-0.444	C
HETATM	162	O	0	0.139	-4.662	-0.912	O
HETATM	163	N	0	-0.372	-2.455	-1.306	N
HETATM	164	C	0	-0.093	-1.267	-0.888	C
HETATM	165	N	0	-0.378	-0.126	-1.576	N
HETATM	166	C	0	0.033	0.933	-0.828	C
HETATM	167	O	0	-0.091	2.144	-1.162	O
HETATM	168	N	0	0.642	0.531	0.341	N
HETATM	169	H	0	0.960	1.137	1.096	H
HETATM	170	C	0	0.654	-0.914	0.438	C
HETATM	171	O	0	1.942	-1.481	0.481	O
HETATM	172	O	0	-1.530	-1.112	1.496	O
HETATM	173	H	0	-1.702	-2.023	1.097	H
HETATM	174	H	0	-2.345	-0.782	2.832	H
HETATM	175	H	0	2.292	-1.559	-0.455	H
HETATM	176	H	0	-1.962	-0.542	-2.382	H
HETATM	177	H	0	3.284	-0.934	-5.225	H
HETATM	178	H	0	-3.150	1.447	6.182	H
HETATM	179	H	0	-4.854	-5.865	-7.597	H
HETATM	180	H	0	-5.410	-4.395	-6.808	H
HETATM	181	H	0	-1.609	-1.078	-7.204	H
HETATM	182	H	0	-3.293	-0.837	-6.770	H
HETATM	183	H	0	5.718	5.078	-6.540	H
HETATM	184	H	0	6.281	5.910	-5.087	H
HETATM	185	H	0	6.189	1.541	-3.225	H
HETATM	186	H	0	1.476	3.803	6.612	H
HETATM	187	H	0	3.080	3.609	5.941	H
HETATM	188	H	0	4.321	0.158	10.053	H
HETATM	189	H	0	5.030	1.038	8.710	H
HETATM	190	H	0	5.772	-1.014	-5.998	H
HETATM	191	H	0	6.713	-2.423	-6.479	H
HETATM	192	H	0	-6.794	2.727	6.249	H
HETATM	193	H	0	-5.052	2.896	6.134	H
HETATM	194	H	0	-5.206	8.730	2.033	H
HETATM	195	H	0	-5.008	9.137	0.319	H
HETATM	196	H	0	-6.340	3.003	1.767	H
HETATM	197	H	0	5.401	0.907	2.804	H

PROc

HETATM	1	C	0	-2.397	-1.386	-6.506	C
HETATM	2	H	0	-2.588	-2.401	-6.787	H
HETATM	3	C	0	-1.998	-1.156	-5.045	C
HETATM	4	H	0	-1.321	-1.950	-4.703	H
HETATM	5	H	0	-1.409	-0.231	-4.981	H
HETATM	6	C	0	-3.156	-1.041	-4.101	C
HETATM	7	N	0	-2.967	-0.692	-2.783	N
HETATM	8	C	0	-4.177	-0.687	-2.177	C
HETATM	9	H	0	-4.300	-0.506	-1.119	H
HETATM	10	N	0	-5.147	-1.006	-3.020	N
HETATM	11	C	0	-4.516	-1.220	-4.230	C

HETATM	12	H	0	-5.075	-1.487	-5.116	H
HETATM	13	C	0	-4.601	-5.100	-6.786	C
HETATM	14	H	0	-3.714	-4.601	-7.116	H
HETATM	15	C	0	-4.552	-5.786	-5.415	C
HETATM	16	H	0	-5.471	-6.382	-5.319	H
HETATM	17	H	0	-3.728	-6.512	-5.385	H
HETATM	18	C	0	-4.456	-4.843	-4.205	C
HETATM	19	H	0	-3.432	-4.469	-4.090	H
HETATM	20	H	0	-5.075	-3.953	-4.356	H
HETATM	21	C	0	-4.917	-5.536	-2.905	C
HETATM	22	H	0	-6.002	-5.672	-2.937	H
HETATM	23	H	0	-4.493	-6.550	-2.842	H
HETATM	24	N	0	-4.626	-4.805	-1.682	N
HETATM	25	H	0	-5.382	-4.239	-1.215	H
HETATM	26	C	0	-3.418	-4.777	-1.110	C
HETATM	27	N	0	-2.411	-5.562	-1.590	N
HETATM	28	H	0	-2.551	-5.943	-2.511	H
HETATM	29	H	0	-1.444	-5.257	-1.441	H
HETATM	30	N	0	-3.226	-4.040	-0.018	N
HETATM	31	H	0	-3.926	-3.306	0.252	H
HETATM	32	H	0	-2.287	-3.989	0.369	H
HETATM	33	C	0	5.740	-2.062	-6.081	C
HETATM	34	H	0	5.021	-2.322	-6.830	H
HETATM	35	C	0	5.468	-2.765	-4.736	C
HETATM	36	H	0	5.310	-3.836	-4.924	H
HETATM	37	H	0	6.363	-2.679	-4.114	H
HETATM	38	C	0	4.335	-2.231	-3.895	C
HETATM	39	N	0	3.442	-1.245	-4.273	N
HETATM	40	C	0	2.648	-0.939	-3.207	C
HETATM	41	H	0	1.868	-0.190	-3.252	H
HETATM	42	N	0	2.977	-1.679	-2.161	N
HETATM	43	C	0	4.012	-2.487	-2.583	C
HETATM	44	H	0	4.491	-3.186	-1.914	H
HETATM	45	C	0	5.644	6.049	-5.942	C
HETATM	46	H	0	6.006	6.842	-6.562	H
HETATM	47	C	0	4.141	6.288	-5.681	C
HETATM	48	H	0	4.031	7.161	-5.026	H
HETATM	49	H	0	3.679	6.576	-6.636	H
HETATM	50	C	0	3.347	5.133	-5.102	C
HETATM	51	C	0	2.728	5.218	-3.849	C
HETATM	52	H	0	2.875	6.110	-3.243	H
HETATM	53	C	0	1.902	4.205	-3.356	C
HETATM	54	H	0	1.422	4.310	-2.387	H
HETATM	55	C	0	1.671	3.060	-4.131	C
HETATM	56	O	0	0.852	2.048	-3.748	O
HETATM	57	H	0	0.409	2.248	-2.889	H
HETATM	58	C	0	2.304	2.945	-5.379	C
HETATM	59	H	0	2.108	2.062	-5.981	H
HETATM	60	C	0	3.122	3.967	-5.850	C
HETATM	61	H	0	3.574	3.867	-6.835	H
HETATM	62	C	0	5.703	1.232	-2.285	C
HETATM	63	H	0	5.709	2.040	-1.584	H
HETATM	64	H	0	4.694	1.004	-2.558	H
HETATM	65	C	0	6.451	0.003	-1.786	C
HETATM	66	O	0	6.779	-0.915	-2.537	O
HETATM	67	N	0	6.725	-0.012	-0.454	N
HETATM	68	H	0	6.500	0.774	0.143	H

HETATM	69	C	0	7.126	-1.203	0.274	C
HETATM	70	H	0	6.525	-2.055	-0.062	H
HETATM	71	C	0	8.607	-1.606	0.085	C
HETATM	72	H	0	8.800	-1.728	-0.984	H
HETATM	73	H	0	9.246	-0.789	0.463	H
HETATM	74	O	0	8.885	-2.839	0.726	O
HETATM	75	H	0	8.355	-2.852	1.543	H
HETATM	76	C	0	6.872	-0.952	1.775	C
HETATM	77	O	0	6.861	0.242	2.159	O
HETATM	78	O	0	6.732	-1.993	2.499	O
HETATM	79	C	0	2.194	3.094	6.273	C
HETATM	80	H	0	2.445	2.512	7.135	H
HETATM	81	C	0	1.653	2.321	5.061	C
HETATM	82	H	0	1.030	1.481	5.399	H
HETATM	83	H	0	0.977	2.968	4.490	H
HETATM	84	C	0	2.711	1.821	4.124	C
HETATM	85	N	0	2.396	1.300	2.882	N
HETATM	86	C	0	3.559	0.975	2.338	C
HETATM	87	H	0	3.683	0.521	1.365	H
HETATM	88	N	0	4.603	1.260	3.151	N
HETATM	89	C	0	4.077	1.802	4.299	C
HETATM	90	H	0	4.704	2.125	5.115	H
HETATM	91	C	0	4.193	0.434	9.016	C
HETATM	92	H	0	3.305	1.029	8.965	H
HETATM	93	C	0	4.201	-0.880	8.219	C
HETATM	94	H	0	4.986	-1.522	8.642	H
HETATM	95	H	0	3.259	-1.423	8.376	H
HETATM	96	C	0	4.467	-0.727	6.715	C
HETATM	97	H	0	3.633	-0.209	6.226	H
HETATM	98	H	0	5.340	-0.083	6.554	H
HETATM	99	C	0	4.728	-2.088	6.040	C
HETATM	100	H	0	5.675	-2.497	6.405	H
HETATM	101	H	0	3.949	-2.805	6.331	H
HETATM	102	N	0	4.802	-2.066	4.584	N
HETATM	103	H	0	5.701	-1.970	4.087	H
HETATM	104	C	0	3.725	-2.182	3.792	C
HETATM	105	N	0	2.483	-2.145	4.313	N
HETATM	106	H	0	2.359	-1.611	5.159	H
HETATM	107	H	0	1.699	-2.091	3.654	H
HETATM	108	N	0	3.887	-2.422	2.489	N
HETATM	109	H	0	4.852	-2.476	2.161	H
HETATM	110	H	0	3.143	-2.175	1.829	H
HETATM	111	C	0	-5.848	2.221	6.378	C
HETATM	112	H	0	-5.785	1.909	7.399	H
HETATM	113	C	0	-5.674	1.016	5.430	C
HETATM	114	H	0	-6.142	0.135	5.891	H
HETATM	115	H	0	-6.210	1.162	4.490	H
HETATM	116	C	0	-4.257	0.697	5.057	C
HETATM	117	N	0	-3.165	0.967	5.868	N
HETATM	118	C	0	-2.036	0.593	5.212	C
HETATM	119	H	0	-1.043	0.724	5.613	H
HETATM	120	N	0	-2.337	0.084	4.033	N
HETATM	121	C	0	-3.714	0.139	3.922	C
HETATM	122	H	0	-4.233	-0.237	3.052	H
HETATM	123	C	0	-5.552	8.542	1.047	C
HETATM	124	H	0	-6.595	8.773	0.982	H
HETATM	125	C	0	-5.268	7.385	0.069	C

HETATM	126	H	0	-6.031	6.612	0.228	H
HETATM	127	H	0	-5.450	7.755	-0.952	H
HETATM	128	C	0	-3.906	6.707	0.112	C
HETATM	129	C	0	-3.775	5.406	-0.401	C
HETATM	130	H	0	-4.658	4.897	-0.783	H
HETATM	131	C	0	-2.552	4.742	-0.448	C
HETATM	132	H	0	-2.478	3.739	-0.857	H
HETATM	133	C	0	-1.395	5.374	0.036	C
HETATM	134	O	0	-0.171	4.789	0.025	O
HETATM	135	H	0	-0.209	3.873	-0.333	H
HETATM	136	C	0	-1.507	6.669	0.561	C
HETATM	137	H	0	-0.612	7.154	0.940	H
HETATM	138	C	0	-2.742	7.320	0.593	C
HETATM	139	H	0	-2.791	8.326	1.004	H
HETATM	140	C	0	-5.813	2.234	1.279	C
HETATM	141	H	0	-5.853	2.372	0.218	H
HETATM	142	H	0	-4.792	2.220	1.598	H
HETATM	143	C	0	-6.464	0.864	1.521	C
HETATM	144	O	0	-6.736	0.427	2.645	O
HETATM	145	N	0	-6.703	0.179	0.383	N
HETATM	146	H	0	-6.573	0.623	-0.518	H
HETATM	147	C	0	-7.253	-1.159	0.263	C
HETATM	148	H	0	-7.807	-1.386	1.181	H
HETATM	149	C	0	-8.214	-1.151	-0.948	C
HETATM	150	H	0	-8.423	-2.192	-1.216	H
HETATM	151	H	0	-9.154	-0.663	-0.658	H
HETATM	152	O	0	-7.705	-0.398	-2.035	O
HETATM	153	H	0	-6.890	-0.818	-2.413	H
HETATM	154	C	0	-6.152	-2.264	0.124	C
HETATM	155	O	0	-6.530	-3.332	-0.435	O
HETATM	156	O	0	-5.006	-2.017	0.588	O
HETATM	157	O	0	0.363	-1.746	2.572	O
HETATM	158	C	0	0.122	-0.830	1.778	C
HETATM	159	N	0	-0.173	-3.852	0.628	N
HETATM	160	H	0	0.225	-4.761	0.833	H
HETATM	161	C	0	0.000	-3.529	-0.719	C
HETATM	162	O	0	0.260	-4.452	-1.506	O
HETATM	163	N	0	-0.356	-2.289	-1.181	N
HETATM	164	C	0	-0.085	-1.098	-0.755	C
HETATM	165	N	0	-0.541	0.013	-1.372	N
HETATM	166	C	0	0.079	1.094	-0.824	C
HETATM	167	O	0	-0.114	2.285	-1.170	O
HETATM	168	N	0	1.036	0.726	0.114	N
HETATM	169	H	0	1.257	1.361	0.878	H
HETATM	170	C	0	0.868	-0.678	0.425	C
HETATM	171	O	0	2.045	-1.433	0.444	O
HETATM	172	O	0	-0.816	0.052	1.934	O
HETATM	173	H	0	0.031	-3.154	1.338	H
HETATM	174	H	0	-1.372	-0.059	2.817	H
HETATM	175	H	0	2.383	-1.516	-0.504	H
HETATM	176	H	0	-2.069	-0.430	-2.341	H
HETATM	177	H	0	3.399	-0.796	-5.175	H
HETATM	178	H	0	-3.205	1.405	6.775	H
HETATM	179	H	0	-4.839	-5.840	-7.561	H
HETATM	180	H	0	-5.408	-4.358	-6.804	H
HETATM	181	H	0	-1.587	-1.047	-7.163	H
HETATM	182	H	0	-3.279	-0.791	-6.766	H

HETATM	183	H	0	5.808	5.105	-6.472	H
HETATM	184	H	0	6.220	6.015	-5.013	H
HETATM	185	H	0	6.199	1.575	-3.199	H
HETATM	186	H	0	1.436	3.807	6.619	H
HETATM	187	H	0	3.069	3.689	5.995	H
HETATM	188	H	0	4.340	0.217	10.081	H
HETATM	189	H	0	5.031	1.071	8.712	H
HETATM	190	H	0	5.771	-0.974	-5.965	H
HETATM	191	H	0	6.719	-2.372	-6.459	H
HETATM	192	H	0	-6.844	2.656	6.247	H
HETATM	193	H	0	-5.120	3.015	6.187	H
HETATM	194	H	0	-5.324	8.258	2.080	H
HETATM	195	H	0	-4.975	9.441	0.808	H
HETATM	196	H	0	-6.354	3.022	1.806	H
HETATM	197	H	0	5.577	0.975	2.954	H
